# Oxidative Release of Natural Glycans: Unraveling the Mechanism for Rapid N-Glycan Glycomics Analysis

**DOI:** 10.1021/acs.analchem.4c03246

**Published:** 2024-10-10

**Authors:** Qing Zhang, Yi Lasanajak, Xuezheng Song

**Affiliations:** † Department of Biochemistry, Emory University School of Medicine, Atlanta, Georgia 30322, United States; ‡ Emory Glycomics and Molecular Interactions Core, Emory University School of Medicine, Atlanta, Georgia 30322, United States

## Abstract

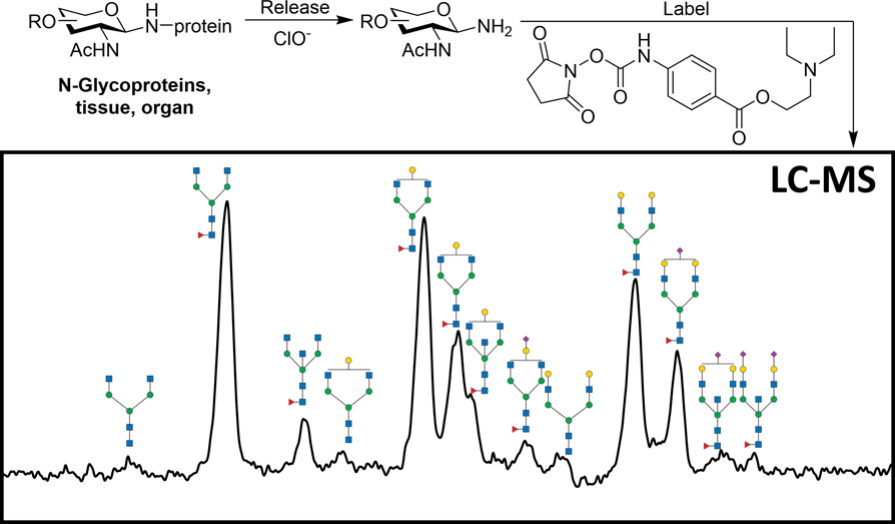

N-glycosylation is a critical post-translational modification involved in various biosynthetic pathways and disease mechanisms. In this study, we present an optimized oxidative release of natural glycans (ORNG) method using household bleach that enables the rapid and efficient release of N-glycans from biological samples. We thoroughly investigated the ORNG mechanism, identifying key intermediates and side products and providing valuable insights into the oxidative release process. The method is highly efficient, releasing a wide range of N-glycans, including high-mannose, hybrid, and complex structures, with minimal sample processing. Our ORNG-based specific N-glycan profiling approach has demonstrated high sensitivity and efficiency, particularly in releasing N-glycans resistant to enzymatic digestion, such as core α3-fucosylated N-glycans from soy protein. Validation through mass spectrometry confirmed the method’s ability to accurately profile N-glycans from complex biological samples, including human serum, with results comparable to traditional PNGase F digestion. The ORNG-based method’s scalability, versatility, and use of low-cost reagents make it especially suited for large-scale glycomics studies. Furthermore, the mass spectrometry data revealed that the ORNG-based method achieves high sensitivity and specificity, positioning it as a robust alternative for comprehensive glycan profiling and functional studies. Our findings highlight ORNG’s potential to advance N-glycomics, offering promising improvements in speed, efficiency, and breadth of glycan analysis.

## Introduction

Glycomics has emerged as a vital field of biomedical research, attracting considerable attention in recent years due to its critical role in understanding biological processes and disease mechanisms.^1,2^ Glycans, complex carbohydrates attached to proteins and lipids, play essential roles in cell–cell communication, immune response, and pathogen recognition.^3^ Despite its potential, glycomics lags behind genomics and proteomics primarily due to the intricate challenges associated with glycan analysis and preparation. The complexity and diversity of glycan structures, along with their nontemplate-driven biosynthesis, pose significant challenges for researchers.

Glycomics is broadly divided into analytical glycomics, which focuses on the profiling of glycan expression and structural elucidation of glycans, and functional glycomics, which investigates the interactions between glycans and glycan-binding proteins, such as lectins. These interactions occur whether the glycans are free or bound to cells, bacteria, viruses, or as part of the immune response through antibody binding.^3,4^ Understanding these interactions is crucial for the development of therapeutic interventions and diagnostic tools.

Comprehensive structural and functional analyses of glycans require significant quantities of glycans and employ advanced techniques including nuclear magnetic resonance (NMR), crystallography, high-performance liquid chromatography (HPLC),^5−7^ mass spectrometry (MS),^8−15^ and glycan microarrays.^16−21^ However, the field is constrained by the lack of efficient methods to produce a diverse repertoire of complex biomedically relevant complex glycans. While natural glycans directly from biological materials represent a logical source to address this problem, release and isolation of these natural glycans often are hampered by a complicated process, expensive reagents such as special enzymes, and/or harsh reaction conditions.^22−26^ These methods, while historically useful for detailed structural studies, are not suitable for the large-scale production needed for functional analyses. This limitation hampers the effort to fully represent the diversity of glycan structures in human and animal glycomes for both analytical and functional studies.

Unlike nucleic acids and proteins, glycans do not have a genetic template for amplification, posing unique challenges for their synthesis. Recent advances in chemical and enzymatic glycan synthesis have made progress,^27−31^ yet replicating the complex structures of natural glycans precisely remains a daunting task. Furthermore, the chemical synthesis required to produce glycans in quantities adequate for detailed studies is not only technically demanding but also cost-prohibitive.

Our previous studies introduced the ORNG method for both analytical and preparative N-glycan release from biological samples.^32,33^ Following AEAB conjugation, N-glycans are separated and purified using two-dimensional HPLC, allowing for further characterization and investigation of their biological activities. The use of an inexpensive reagent, household bleach, coupled with a rapid reaction process, makes this method exceptionally suitable for large-scale glycan production—from animal and plant glycoconjugates—yielding up to 100 mg of pure glycans.^34^ However, the yield of free reducing N-glycan released is lower than that achieved by traditional PNGase F digestion. Notably, a substantial portion of glycans without a free reducing end is released, thereby preventing their conjugation with tags through reductive amination. This observation has led us to further refine and explore the oxidative release of the N-glycan process in detail, aiming to further understand the reaction mechanism and side products, enhance the yield of released glycans, utilize the unconjugated glycans more effectively, and widen the application of the ORNG process in glycomics analysis.

## Experimental Section

### Oxidative Release of N-Glycan from Sialylglycopeptide (SGP)

To release N-glycans from SGP, we prepared aqueous solutions of SGP with a concentration of 50 mg/mL and calcium hypochlorite (Ca(ClO)_2_) with a concentration of 50 mg/mL. The Ca(ClO)_2_ solution was centrifuged (1 min, 10000 rpm) to remove any insoluble material. We then mixed 50 μL of each solution, a ratio determined to be optimal for N-glycan release based on preliminary experiments (Figure S1). The mixture was allowed to react for 15 min. Postreaction, acetic acid was added to acidify the solution and halt the reaction. The acidified mixture was subsequently centrifuged to precipitate and remove partially oxidized nonglycan materials. The clear supernatant, containing the released glycans, was first subjected to size exclusion chromatography using a Bio-Gel P-2 column with deionized (DI) water as the eluent. Following this, the glycans were further purified using solid-phase extraction (SPE) on a reversed-phase C18 cartridge, also eluted with DI water. To ensure minimal loss of glycans during the purification process, each step was monitored by using thin-layer chromatography (TLC) staining with orcinol. The final fraction containing N-glycans was then analyzed using liquid chromatography–mass spectrometry (LC-MS). During the chromatographic process, mobile phase A was incrementally increased from 18% to 60% over 50 min.

### N-Glycan Oxidative Release and Specific Labeling of Glycoproteins

We prepared aqueous solutions of the glycoproteins at a concentration of 5 mg/mL and of Ca(ClO)_2_ at 10 mg/mL. The Ca(ClO)_2_ solution was centrifuged at 10,000 rpm for 1 min to remove any insoluble material. Subsequently, 10 μL of the glycoprotein solution was mixed with 5 μL of the Ca(ClO)_2_ solution. This mixture was vortexed at room temperature for 1 min to react. To quench the reaction, 5 μL of a 10 mg/mL sodium sulfite (Na_2_SO_3_) aqueous solution was added. Following this, 20 μL of NHS-NH-(diethylamino)ethyl benzoate dissolved in DMSO at a concentration of 50 mg/mL was added to the mixture. The reaction mixture was then heated at 50 °C for 5 min. After heating, the mixture was centrifuged at 10,000 rpm for 3 min to remove any precipitated material. The resulting supernatant was purified using a Hypercarb 96-well plate and eluted with a solution of 50% ACN containing/0.1% trifluoroacetic acid (TFA). The eluate was then lyophilized and reconstituted in 100 μL of water. The sample is ready for LC-MS analysis with mobile phase A incrementally increased from 18 to 46% over 50 min.

### N-Glycan Oxidative Release and Specific Labeling of Human Lung Cancer Serum

10 μL of the human serum sample is mixed with 10 μL of Ca(ClO)_2_ solution (50 mg/mL, centrifuged) and vortex at room temperature for 1 min. 5 μL of Na_2_SO_3_ aqueous solution (100 mg/mL) is added to quench the reaction. Then, 20 μL of NHS-NH-(diethylamino)ethyl benzoate DMSO solution (50 mg/mL) is added to the reaction mixture and heated at 50 °C for 5 min. Then the reaction mixture is centrifuged at 10,000 rpm for 3 min. The supernatant is purified on a Hypercarb 96-well plate and eluted with 50% ACN 1% TFA solution. After lyophilization and dissolving in 100 μL of water, the sample is ready for LC–MS analysis with mobile phase A incrementally increased from 18% to 38% over 50 min. Abundant extracted spectrum ion counts for a panel of glycans were collected, and relative abundance was calculated (Table S1−S5). A one-way ANOVA (Analysis of Variance) test was conducted to compare the differences in relative abundance among normal human serum and 4 stages human lung cancer serums [*n*(normal) = 3, *n*(stage1) = 3, *n*(stage2) = 3, *n*(stage3) = 5, *n*(stage4) = 6]. No significant difference was found among groups for the following glycan (Figure 6 #57, #60, #62–66). A two-sided Student’s *t* test was performed to compare normal serum to different stage lung cancer serum with a significant *p* value equal or below 0.05 for these selected glycans.

## Results and Discussion

### Bleach Releases N-Glycan of Sialylglycopeptide

Sialylglycopeptide (SGP), sourced from egg yolk powder (∼8 mg/yolk),^35^ is a well-identified N-glycopeptide, and it is economically available in large quantities. While as a glycopeptide SGP may not be an ideal model for optimizing reactions between glycoproteins and bleach, it serves as an excellent substrate for studying the intricate details of N-glycan-related product formation during bleach reactions. We subjected SGP with calcium hypochlorite (Ca(ClO)_2_) treatment to release N-glycan. Ca(ClO)_2_ was chosen over sodium hypochlorite (NaClO) because it functions similarly to NaClO but offers the advantage of being a stable solid that is easy to store and convenient to adjust to the desired concentration. Additionally, the concentration of NaClO can vary in commercial sources, making Ca(ClO)_2_ a more reliable choice. The reaction mixture was quenched by acetic acid and purified by precipitation, Bio-Gel P-2 size exclusion chromatography (SEC) and a reversed C18 SPE cartridge. After purification, we analyzed the sample on LC–MS with a hydrophilic interaction liquid chromatography (HILIC) column and acquired the liquid chromatography quadrupole time-of-flight MS profile, as shown in Figure S2. The molecular weight of the free reducing glycan of egg yolk glycopeptide (EYG, Hex_5_HexNAc_4_NeuAc_2_) is 2222.8 (*M*_EYG_). In positive ionization mode, five major products**1**–**5** are detected with 0, −3, +16, +109, and −5 Da mass shift from M_EYG_. Elucidation of the structures of these five major products will help us understand more about the reaction mechanism. Based on the common fragments on the MS/MS profile of five major products, especially the fragment of Hex_5_HexNAc_3_NeuAc_2_, we can tell that the oxidation reaction only occurs at the reducing end of the released N-glycan (Figure S3). The product **1** (*M*_EYG_) is assigned as the free reducing glycan (Glycan-OH), which is presumably hydrolyzed from the corresponding glycosylamine (Glycan-NH_2_). When bleach-treated SGP samples are immediately analyzed using LC–MS after bleach treatment, the glycosylamine peak is prominently detected (Figure S4). Additionally, treatment of the SGP bleach reaction mixture with benzaldehyde and sodium cyanoborohydride results in the formation of a reductive amination product (Figure S4). These results collectively confirm that glycosylamine is generated early in the reaction process. According to the MS data and former report,^34^**2** (M_EYG_-3 Da) is characterized as glycan-nitrile. Product **3** (M_EYG_+16 Da) matches glycan carboxylic acid (Glycan-COOH) from overoxidation at the anomeric position, which is consistent with the oxidative nature of bleach.

**Figure 1:**
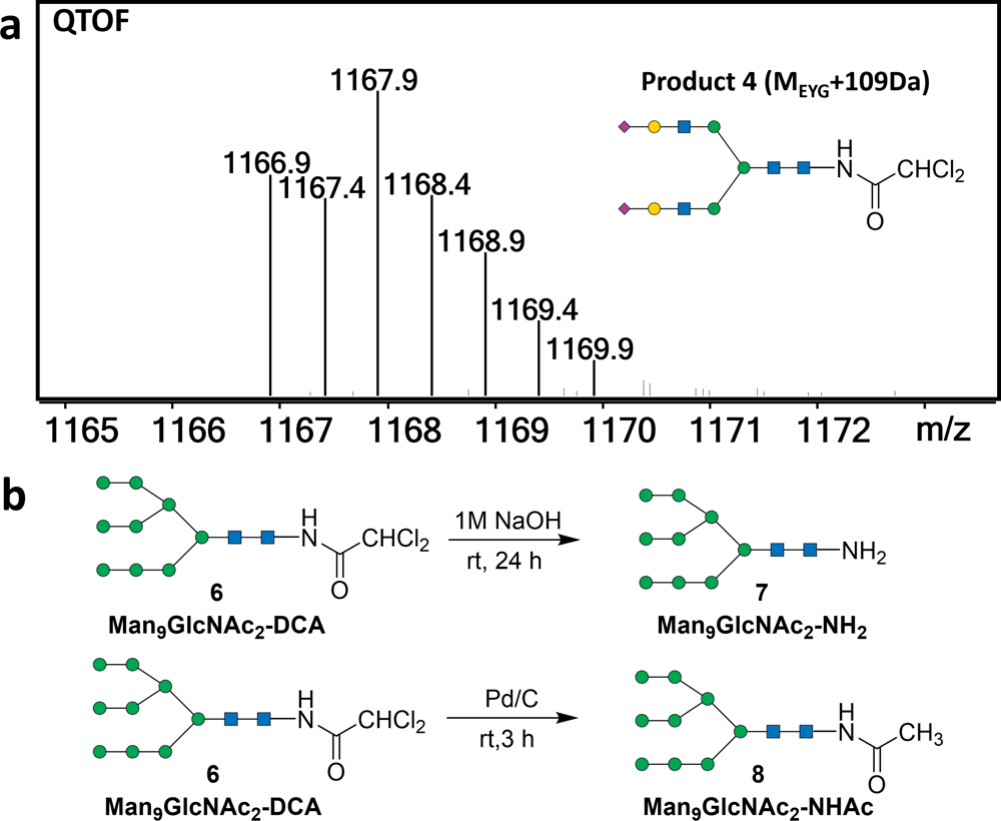
(a) MS isotope pattern of product **4**; (b) reaction scheme of Man_9_GlcNAc_2_-DCA treated with 1 M NaOH solution and Man_9_GlcNAc_2_-DCA treated with Pd/C.

**Scheme 1:**
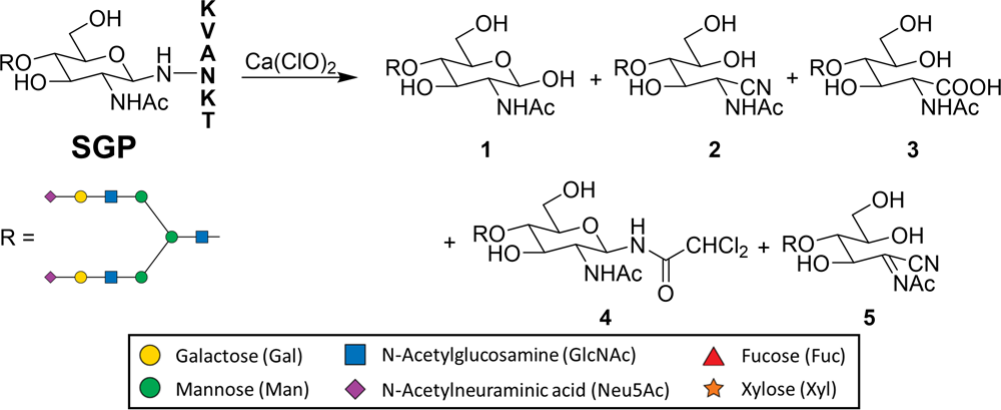
Reaction Scheme of SGP Treated with Bleach

We then sought to identify products **4** (M_EYG_+109 Da) and **5** (M_EYG_-5 Da). Based on the special MS isotope pattern of a chlorine atom, product **4** is inferred to possess a dichlorinated structure (Figure 1a). Isolating pure and significant amounts of product **4** from the reaction mixture is a challenging task. However, we successfully identified and purified a side product from the ORNG product from lima bean^36^ with a similar MS isotope pattern matching a composition of Man_9_GlcNAc_2_ + 109 Da (compound **6**), apparently a derivative of Man9 containing the same aglycon moiety as product **4** (M_EYG_ + 109 Da). This is consistent with the fact that Man9 is the dominant major N-glycan from Lima bean. The characterization of compound **6** using ^1^H, ^13^C, and HSQC NMR spectroscopy revealed distinctive chemical shifts at δ 6.20 (^1^H) and δ 65.71 (^13^C) (Figures S5a and S34–S36), which were consistent with the presence of a dichloroacetic amide group. Therefore, this side product is deduced to be glycosylamine dichloroacetamide (glycan-DCA). In order to further confirm this structure, we subjected the Man_9_GlcNAc_2_-DCA with 1 M NaOH solution at room temperature for 24 h, yielding Man_9_GlcNAc_2_–NH_2_ (Figures 1b and S5b). Additionally, reduction of Man_9_GlcNAc_2_-DCA by palladium on carbon (Pd/C) yielded the N-acetamide of Man_9_GlcNAc_2_–NH_2_ (Man_9_GlcNAc_2_–NHAc). These findings confirmed the glycan-DCA structure, as the dichloroacetamide is expected to hydrolyze under basic conditions and be reduced to Glycan-NHAc by Pd/C. Consequently, the product **4** (M_EYG_ + 109 Da) is identified as a glycan-DCA structure (Scheme 1).

**Figure 2:**
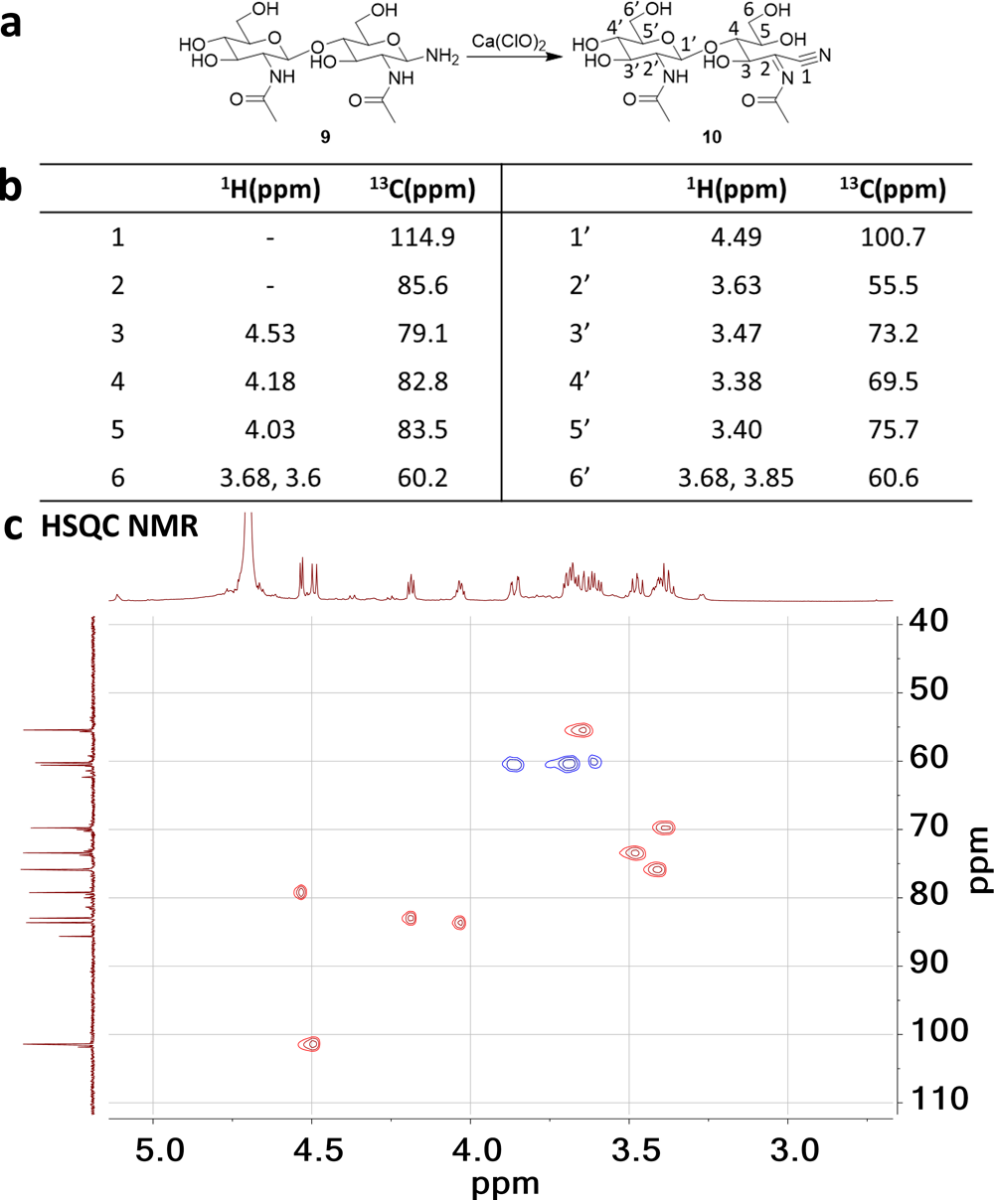
(a) Reaction scheme of chitinbiose amine treated with Ca(ClO)_2_; (b) proton and carbon assignment of compound **10**; and (c) HSQC NMR data of compound **10**.

In our subsequent experiments, we aimed to elucidate the structure of product **5** (M_EYG_-5 Da). This is apparently an overoxidation product, likely from glycan-NH_2_ and glycan-nitrile. We treated the SGP bleach reaction mixture with Pd/C in 5% ammonium formate aqueous solution (Figure S6). Three major products were observed after reduction, matching glycan-NAc, glycan-alditol, and glycan-COOH, respectively. It is known that glycan–OH and glycan-nitrile can be reduced to glycan-alditol. Glycan-COOH is expected to be resistant toward Pd/C reduction and glycan-NAc is likely from the reduction of glycan-DCA. It becomes reasonable to propose that product **5** could also be reduced to glycan–alditol. These reaction outcomes imply a structural relationship between products **2** and **5**. Given the challenges associated with isolating pure product **5** from the SGP mixture, coupled with its relatively large molecular size, which limits detailed NMR analysis, we opted to use chitinbiose as a smaller model compound. From treating chitinbiose amine (**9**) with Ca(ClO)_2_, we were able to isolate a pure compound **10** with a 5 Da mass shift from chitinbiose (*M*_chitinbiose_-5 Da) by silica gel purification. Subsequent analysis involving ^1^H, ^13^C, HSQC, and COSEY NMR (Figures S37–S40) confirmed the structure with a nitrile group and a C=N double bond at the reducing end of chitinbiose, as depicted in Figure 2. Thus, the structure of product **5** (M_EYG_-5 Da) is defined and presented in Scheme 1.

### Proposed Mechanism of Bleach Reaction

**Figure 3:**
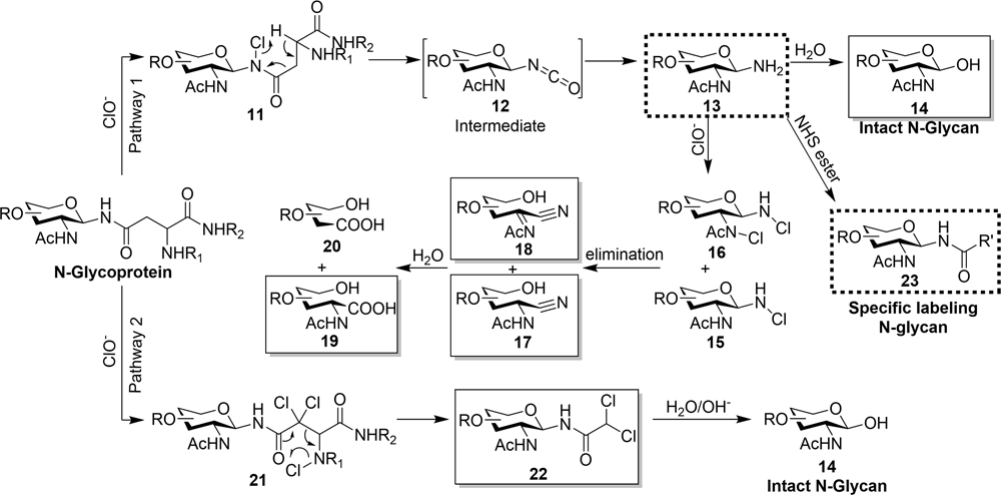
Proposed mechanism of the bleach reaction.

Based on the five major products identified, we propose a more detailed ORNG reaction mechanism divided into two pathways, each determined by the specific amide group position undergoing chlorination (Figure 3). In pathway 1, the initial step involves the chlorination of the amide nitrogen linking N-acetylglucosamine to protein-bound asparagine **11**. This chlorinated species then undergoes pericyclic rearrangement to break the carbon–carbon bond to release N-glycan from the protein backbone in the form of major intermediate glycosyl isocyanate **12**, which, upon hydrolysis, yields glycosylamine **13**. The glycosylamine is gradually hydrolyzed to produce free reducing glycan **14**. In the presence of excess hypochlorite, the glycosylamine undergoes further chlorination **15**&**16** and is eliminated into glycan nitriles **17**&**18**. Ultimately, glycan carboxylic acids **19**&**20** are formed through the hydrolysis of glycan nitriles. In pathway 2, chlorination occurs at the asparagine amide bond and backbone of **21**. This is followed by a rearrangement, leading to the cleavage of a C–C bond and the formation of glycan-DCA compound **22**. Glycan-DCA remains stable under acidic and neutral conditions and can be further hydrolyzed into intact N-glycan **14** under basic conditions, as we previously demonstrated.

A better understanding of the ORNG mechanism on N-glycan release is essential for further optimization of the N-glycan production procedure and the utilization of released glycans not in the form of free reducing glycan. Furthermore, it may imply more advanced applications based on ORNG, such as MALDI-imaging of N-glycans. In this mechanism, we noticed that glycosylamine is a unique intermediate that provides specific reactivity that might be adopted for a specific N-glycan analysis. When glycomics analysis is transitioned from simple glycopeptides or glycoprotein to a more complex biological specimen, the reaction environment becomes considerably more complex and glycan products could become very complicated. For N-glycan analysis, PNGase F or PNGase A is widely used so that specific N-glycomics can be achieved. ORNG has the advantage of releasing all N, O- and glycosphingolipid-glycan, which, on the other hand, brings in complications in glycomics analysis. Glycosylamine, exclusively formed from N-glycans during bleach reactions, is a unique and crucial intermediate. Before hydrolyzing to yield free reducing glycans, glycosylamine can be trapped and tagged specifically via amidation reaction with activated carboxylic acid such as NHS ester or acyl chloride. After this labeling process, the modified N-glycans **23** are amenable to selective purification and efficient profiling through HPLC and/or mass spectrometry, facilitating their differentiation from both other types of glycan and N-glycans released in other forms.

**Scheme 2:**
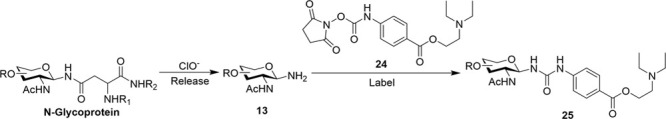
Glycoprotein N-Glycan Oxidative Release and Specific Labeling Reaction

Here, we have developed a protocol for the oxidative release and specific labeling of N-glycans (Scheme 2). Our proposed mechanism emphasizes the critical need to promptly quench the bleach reaction to prevent further chlorination and degradation of the glycosylamines. To achieve this, reductant sodium sulfite (Na_2_SO_3_) is employed to neutralize the excess bleach and/or chloroamine and chloroamides of glycans, with slightly basic conditions (pH 8–9) provided by the Na_2_SO_3_ solution aiding in the stabilization of glycosylamines. These stabilized glycosylamines **13** are then labeled with an NHS ester tag, specifically procaine carbonyl NHS ester (Pro-NHS) **24,** which is used in commercially available AdvanceBio InstantPC Labeling kit.^37^ This procaine derivative **25** not only facilitates detection via HPLC-UV or fluorometric methods but also enhances the mass spectral signals of the labeled N-glycans through its tertiary amine group. We further demonstrated the robustness of our method by successfully labeling glycosylamines from Ca(ClO)_2_-treated human IgG with Fmoc-Cl (Figure S7), confirming the method’s versatility and effectiveness. Given the higher sensitivity in MS of the tertiary amine on the procaine tag, we opted for Pro-NHS over Fmoc-Cl for the specific labeling of N-glycans.

### N-Glycan Oxidative Release and Specific Labeling of Glycoproteins

**Figure 4:**
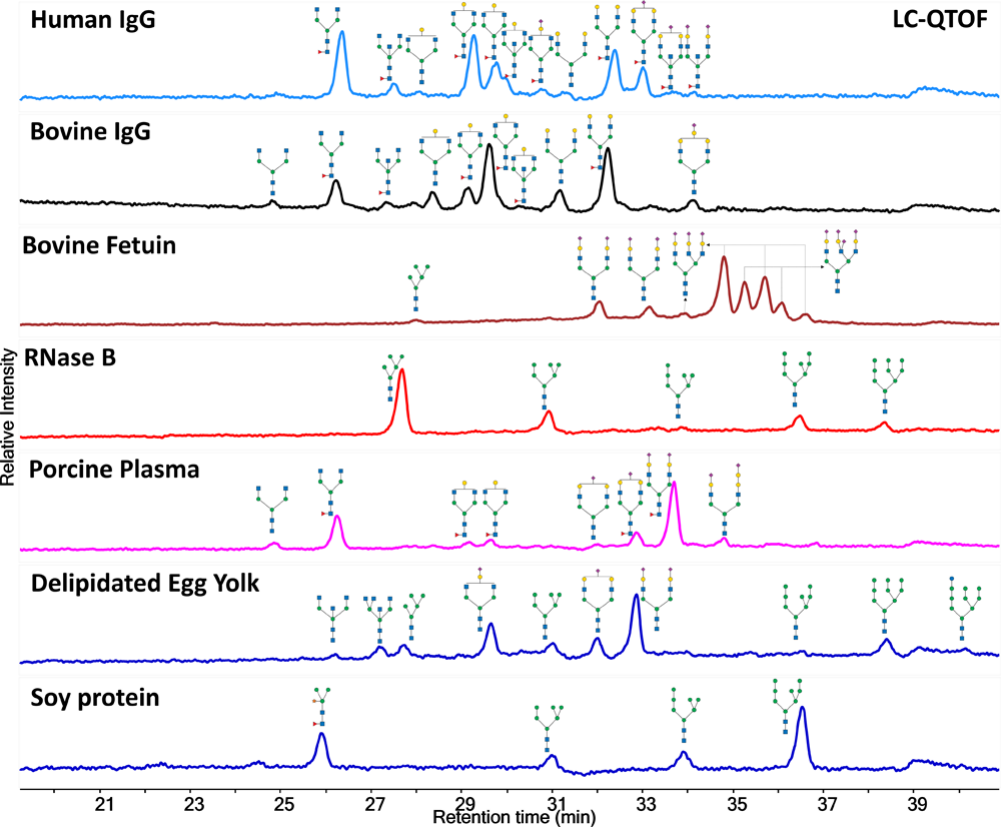
N-Glycan LC–MS profile generated by N-glycan oxidative release and specific labeling protocol of human IgG, bovine IgG, bovine fetuin, RNase B, porcine plasma, delipidated egg yolk, and soy protein.

We applied our oxidative release and specific labeling protocol to comprehensively profile N-glycans of a range of common glycoproteins including human and bovine immunoglobulin G (IgG), bovine fetuin, ribonuclease B (RNase B), porcine plasma powder, delipidated egg yolk powder, and soy protein powder. We also conducted comparative analyses using the commercially available N-glycan sample preparation kit, AdvanceBio Gly-X N-Glycan Prep with InstantPC Kit, which utilizes PNGase F for N-glycan release.^38,39^ The N-glycan compositions and structures were proposed based on mass identification. The TIC chromatograms shown in Figure 4 were labeled with a cartoon of proposed structures(Figures S14–S26). As illustrated in Figure 4, our bleach-based method demonstrated efficient release of all types of N-glycans—high mannose, complex, and hybrid—mirroring the efficacy of the commonly used PNGase F method (Figure S8). Based on the intensity of MS data (Figure S9), the percentage of glycosylamine generated from the bleach reaction is approximately one-third of that released by PNGase F. Notably, our method was able to release core α3-fucosylated N-glycans (structure confirmed with MS/MS Spectra in Figure S10) from soy protein, which are resistant to PNGase F, highlighting the robustness and specificity of our bleaching technique. We also treated human IgG with NaClO to release the N-glycans. After labeling with Pro-NHS and profiling using LC-MS, the NaClO-treated sample produced similar results to those obtained with Ca(ClO)__2__ (Figure S11).

### N-Glycan Profiling and Analysis of Multiple Bean Species

The traditional methods of N-glycosidase digestion require several preprocessing steps such as protein-extraction, denaturing, buffering, etc. to ensure the efficiency of digestion, often making the N-glycan analysis a lengthy process. In contrast, our ORNG-based protocol for N-glycan release and labeling offers a robust, efficient, and general approach that can be applied even to crude materials with minimal processing. In this study, we directly used dry beans as materials to analyze the N-glycan profiles of various bean types. Purchased from a local farmers market, the beans were peeled and ground into powder, and each kind of bean’s powder was suspended separately in water for processing using our N-glycan oxidative release and specific labeling protocol.

**Figure 5:**
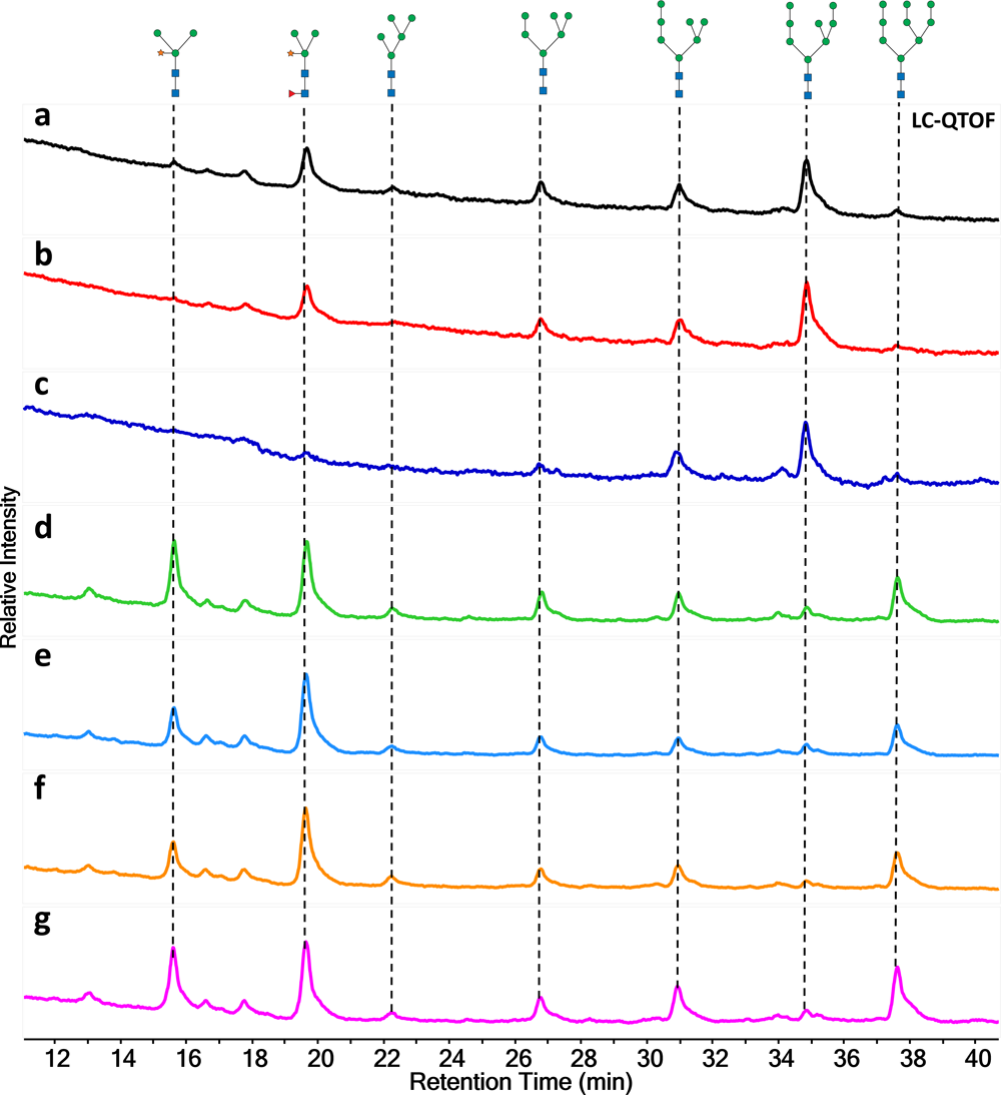
N-Glycan LC–MS profile generated by N-glycan oxidative release and specific labeling protocol of (a) soybean; (b) black bean; (c) red bean; (d) dark red kidney bean; (e) great northern bean; (f) white bean; and (g) pinto bean.

Figure 5 illustrates the LC–MS N-glycan profiles of seven different beans: soybean, black bean, red bean, dark red kidney bean, great northern bean, white bean, and pinto bean (Figures S27–S33). The results reveal consistent structural features across all bean types but notable variations in the relative abundances of specific glycan fractions. For instance, in the profiles of soybean and black bean, the abundance of Man_3_GlcNAc_2_Xyl is much lower compared to Man_3_GlcNAc_2_XylFuc, unlike the profiles observed in dark red kidney bean, great northern bean, white bean, and pinto bean. Additionally, the percentage of Man_8_GlcNAc_2_ in soybean, black bean, and red bean is higher than that of Man_9_GlcNAc_2_, whereas in dark red kidney bean, great northern bean, white bean, and pinto bean, Man_9_GlcNAc_2_ dominates over Man_8_GlcNAc_2_. The diversity in the glycan profiles suggests differing glycosylation patterns that could be linked to various genetic factors or environmental conditions influencing bean cultivation. Interestingly, Man_9_GlcNAc_2_ is scarcely detected in the N-glycan profile of soy protein powder, a protein isolate commonly used as a protein food supplement (Figure 4), whereas it is present in significant quantities in the N-glycan profile of unprocessed soybean. It is well-known that soybean agglutinin, a major glycoprotein isolated from soybean, carries Man_9_GlcNAc_2_ as its major glycosylation.^40,41^ Our results suggest that soybean agglutinin is likely lost during the industrial preparation of soy protein isolate. Therefore, the ability of the ORNG protocol to be directly applied to crude samples with minimal processing may be crucial to acquiring a more accurate depiction of the original N-glycan profile of the biological specimen, avoiding bias related to unnecessary sample processing. This comparative analysis not only underscores the efficacy of our bleach-based method in simplifying the glycan analysis process but also highlights the unique glycan distribution patterns among different bean varieties, potentially opening avenues for further biochemical and nutritional studies.

### Human Lung Cancer Patient Serum N-Glycan Analysis

**Figure 6:**
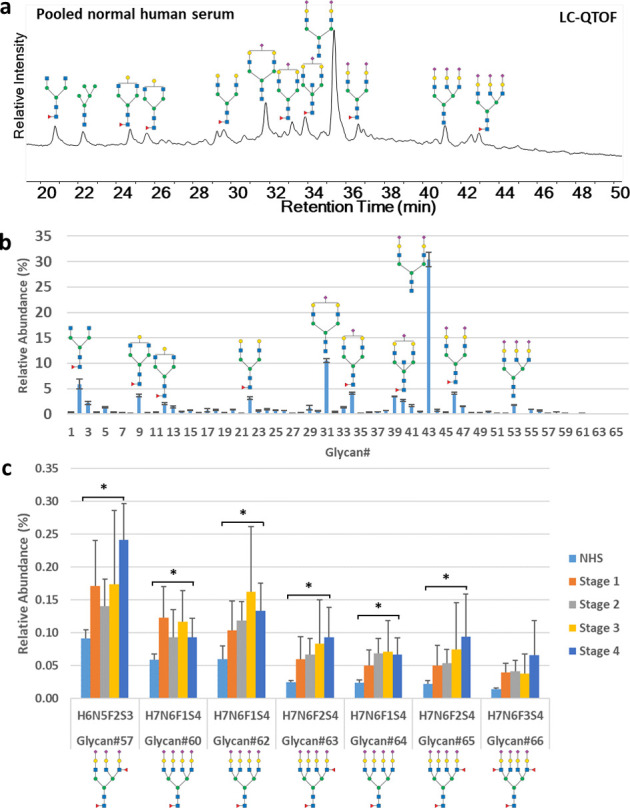
(a) N-glycan LC-MS profile of a normal human serum pooled sample generated by N-glycan oxidative release and specific labeling protocol; (b) EIC N-glycan relative abundance percentage of pooled normal human serum; and (c) comparison of highly branched sialylated and fucosylated N-glycan relative abundance percentage between different human lung cancer stages and normal human serum (NHS). *p < 0.05.

To further validate our novel ORNG-based protocol for N-glycan release and specific labeling, we investigated the N-glycan profiles of a panel of human lung cancer sera. The study encompassed both a pooled normal human serum sample and 17 lung cancer serum samples distributed across different disease stages (three in Stage I, three in Stage II, five in Stage III, and six in Stage IV). These samples underwent treatment using our oxidative N-glycan release and labeling protocol with subsequent profiling by LC-MS (Figures 6a and S12–S14).

We identified 66 N-glycan structures (45 monosaccharide compositions) in the pooled normal human serum sample (Figure 6b, and they are detailed in Table S1). These structures were consistently present across the disease stages and thus were quantitatively monitored throughout the study (Tables S2–S5). All the N-glycan data are analyzed based on EIC abundance. The identified glycans structures include not only common and more abundant glycan compositions such as H3N4F1 (G0F), H4N4F1 (G1F), H5N4F1 (G2F), H5N4F1S2 (G2FS2), etc., but also high charged multiantennary glycans with multiple fucosylation (H6N5F2S3, H7N6F3S4) at low natural abundance, demonstrating the sensitivity of this method. Our findings indicate that specific serum N-glycans, particularly those that are highly branched and rich in sialylated and fucosylated motifs, exhibit significant increases in abundance correlating with the progression of lung cancer (Figure 6c). This finding is consistent with previous reports on the relationship between high levels of sialylation and fucosylation of N-glycans in normal human serum and in the serum of lung cancer patients.^42−45^ This trend is markedly pronounced between the different cancer stages, underscoring the potential of these N-glycans as biomarkers for the early detection of lung cancer.

Early diagnosis is pivotal for improving the 5-year survival rates and mitigating treatment costs associated with lung cancer.^46^ Our study not only reaffirms the utility of the ORNG-based N-glycan profiling approach but also enhances our capability to discern early stage lung cancer in patients compared to healthy controls. This advancement holds significant promise for integrating N-glycan signatures into routine diagnostic protocols, thereby facilitating timely and accurate cancer detection. The further glycomics study of more patient sera, in concert with genetic and pathological studies, may eventually result in high fidelity glycan biomarker development.

## Conclusions

Glycomics analysis is an essential component of glycoscience and has recently become a new avenue for biomedical research. There have been multiple methods developed for glycomics analysis, most of which start at the release of glycans from biological samples. Oxidative release of natural glycans by household bleach represents the most recent development holding great potential owing to its simplicity and scalability. However, the strong oxidant nature of hypochlorites generates overoxidation products, limiting its wide application and adoption, especially in the analytical glycomics. In this study, we carried out a detailed study on the ORNG products to provide an insightful mechanism of ORNG. Analysis of the mechanism enabled the design and development of a general and specific N-glycan profiling method through the trapping of a glycosylamine intermediate.

This method provides significant advancement over traditional enzymatic release methods. This method is not only cost-effective but also capable of high-throughput glycan profiling on various biological samples, including those that might be resistant to commonly used PNGase F enzyme. A comparison of the ORNG method with current N-glycan release methods is provided in Table S6. The labeling chemistry, unlikely more traditionally used reductive amination, is specific toward N-glycans, avoiding potential interference of other classes of glycans and glycoconjugates. The ability to efficiently release and specifically label N-glycans from a variety of biological samples, including glycoproteins, crude plant materials, and human serum, enables detailed glycomics analysis, thus facilitating deeper insights into glycan-mediated biological processes. The successful profiling of N-glycans from these sources underscores the method’s robustness and versatility. The comparative analysis of N-glycan profiles across different bean varieties highlights the method’s potential for agricultural and nutritional research.

In the context of disease diagnostics, our method demonstrated a significant correlation between specific N-glycan structures and the progression of lung cancer. This finding supports the potential of N-glycan profiling as a noninvasive biomarker for early cancer detection, which could be integrated into routine clinical diagnostics to improve patient outcomes. Future studies may explore the integration of this method into routine analytical protocols, potentially enhancing the diagnostic capabilities for diseases in which glycan alterations are pivotal. Additionally, further refinement of the bleach reaction conditions could improve glycan yield and purity, expanding the method’s applicability in various research and industrial settings.

In conclusion, our bleach-based N-glycan release and specific labeling method offer a promising approach for glycomics research, providing a practical, efficient, and scalable solution for the comprehensive analysis of N-glycans from a wide range of biological sources.

## Supporting Information Available

Materials and methods, additional experimental details, copies of mass spectra, and copies of NMR spectra (PDF)
