# Supplementary material for: Oxidative Release of Natural Glycans: Unraveling the Mechanism for Rapid N-Glycan Glycomics Analysis
Source: Anal Chem. 2024 Oct 10;96(42):16750–7. doi: 10.1021/acs.analchem.4c03246 (PMC11503514; doi:10.1021/acs.analchem.4c03246)

Supporting Information

Oxidative Release of Natural Glycans (ORNG): Unraveling the Mechanism for Rapid N-Glycan Glycomics Analysis

Qing Zhang[a], Yi Lasanajak[b] and Xuezheng Song[ab]*

[a]*Department of Biochemistry, Emory University School of Medicine, Atlanta, GA 30322, USA*

[b]*Emory Glycomics and Molecular Interactions Core, Emory University School of Medicine, Atlanta, GA 30322, USA*

*Email: xsong2@emory.edu

Table of Contents

I. Materials and methods 2

II. Supplementary Figure 4

III. Supplementary Table 18

IV. Mass Spectra 24

V. NMR Spectra 43

I. Materials and methods

Materials. All chemicals and HPLC solvents were purchased from Sigma-Aldrich, Acros, Oakwood chemicals or Fisher Scientific. The AdvanceBio Gly-X N-Glycan Prep with InstantPC kit was purchased from Agilent Technologies. Milli-Q water was used to prepare all aqueous solutions. Calcium hypochlorite ($Ca(ClO)_2$) was obtained from Drytec®, containing 68% $Ca(ClO)_2$ and a minimum of 65% available chlorine. Any commercial source of $Ca(ClO)_2$ could potentially be used for this method but should be validated for the $Ca(ClO)_2$ percentage. The flash C18 column was from Agela Technologies. The hypercarb 96 well plate was from Thermal Scientific. All the dry beans were purchased from a local supermarket. The pooled normal human serum was purchased from Seracare life sciences and human lung cancer serum samples were purchased from Innovative Research. An Agilent AdvanceBio 6545XT LC/Q-TOF were used for mass spectrometry analysis. ^{1}H and ^{13}C NMR spectra were recorded on a Bruker NEO 400 (400 MHz), and Bruker AVANCE 600 (600 MHz) spectrometer at 25 ^{o}C.

LC-MS Analyses. An Agilent 1290 Infinity II LC system was used and was coupled with an Agilent 6545XT AdvanceBio LC/Q-TOF. An Agilent AdvanceBio Glycan Mapping column (2.1 × 150 mm, 1.8 µm) was used at 40 ◦C to prompt efficient separation. The flow rate of the nanopump was set to 0.4 mL/min. Mobile phase A consisted of 50 mM ammonium formate aqueous solution (pH=4.4), while mobile phase B is 100% ACN. In positive ion mode, the ion source was set to a dry gas flow of 9 L/min, drying temperature of 150 °C, and a nebulizer pressure of 35 psi. An additional in-source CID voltage of 15 eV was applied for MS/MS. The time for recording a spectrum was set to 0.5 s with aspectral width of m/z 500-3200. Raw files from LC-MS were analyzed and quantified using Agilent MassHunter Qualitative Analysis (version 10.0).

N-glycan oxidative release and Fmoc labeling of Human IgG for analysis. We prepared aqueous solutions of the Human IgG at a concentration of 50 mg/mL and of $Ca(ClO)_2$ at 100 mg/mL. The $Ca(ClO)_2$ solution was centrifuged at 10,000 rpm for 1 minute to remove any insoluble material. Subsequently, 10 µL of the Human IgG solution was mixed with 5 µL of the $Ca(ClO)_2$ solution. This mixture was vortexed at room temperature for 1 minute to initiate the reaction. To quench the reaction, 5 µL of a 100 mg/mL sodium sulfite (Na_2SO_3) aqueous solution was added. Following this, 20 ul 0.25 M borate buffer (pH=8) and 20 µL of Fmoc-Cl dissolved in ACN at a concentration of 50 mg/mL was added to the mixture. The reaction mixture was then heated at 65°C for 30 minutes. After heating, the mixture was centrifuged at 10,000 rpm for 3 minutes to remove any precipitated material. The resulting supernatant was purified using a hypercarb 96-well plate and eluted with a solution of 50% ACN contained/0.1% trifluoroacetic acid (TFA). The eluate was then lyophilized and reconstituted in 100 µL of water. The sample is ready for LC-MS analysis with mobile phase A incrementally increased from 18% to 46% over 50 minutes.

N-glycan oxidative release and specific labeling of beans for analysis. The dry beans were peeled and ground into powder, and each kind of bean's powder was suspended separately in water with a concentration of 10 mg/ml. 10 µL of beans solution is mixed with 5 µL of $Ca(ClO)_2$ solution (20 mg/mL, centrifuged) and vortex in room temperature for 1 minutes. 5 µL of Na_2SO_3 aqueous solution (20 mg/mL) is added to quench the reaction. Then 20 µL of NHS-NH-(diethylamino)ethyl benzoate DMSO solution (50 mg/mL) is added to the reaction mixture and heated in 50 ◦C for 5 mins. Then the reaction mixture is centrifuged at 10000 rpm for 3 minutes. The supernatant is purified on hypercarb 96-well plate and eluted with 50% ACN/0.1% TFA solution. After lyophilized and dissolved in 100 µL water, the

sample is ready for LC-MS analysis with mobile phase A incrementally increased from 18% to 38% over 50 minutes.

$Man_9GlcNAc_2$-DCA (**6**), ^1H NMR (600 MHz, D_2O) δ 6.20 (s, 1H), 5.32 (d, *J* = 1.7 Hz, 1H), 5.25 (d, *J* = 1.7 Hz, 1H), 5.22 (d, *J* = 1.8 Hz, 1H), 5.06 (d, *J* = 1.8 Hz, 1H), 5.03 (d, *J* = 9.7 Hz, 1H), 4.97 (d, *J* = 1.8 Hz, 1H), 4.96 (d, *J* = 1.8 Hz, 1H), 4.95 (d, *J* = 1.8 Hz, 1H), 4.78 (d, *J* = 1.8 Hz, 1H), 4.68 (s, 1H), 4.52 (d, *J* = 7.7 Hz, 1H), 4.14 (d, *J* = 3.2 Hz, 1H), 4.09 – 3.47 (m, 66H), 1.98 (s, 3H), 1.90 (s, 3H). ^{13}C NMR (151 MHz, D_2O) δ 174.75, 174.49, 167.18, 102.25, 102.21, 102.19, 101.32, 100.82, 100.59, 100.22, 99.60, 97.97, 81.09, 79.07, 78.91, 78.87, 78.63, 78.60, 78.54, 78.45, 76.55, 74.53, 74.14, 73.39, 73.27, 73.26, 73.21, 73.17, 73.16, 72.69, 72.39, 71.89, 71.16, 70.29, 70.21, 70.04, 69.95, 69.93, 69.38, 67.01, 66.94, 66.90, 66.88, 66.85, 66.82, 66.76, 65.71, 65.53, 65.41, 65.07, 64.95, 61.14, 61.11, 61.06, 61.02, 60.99, 60.97, 60.92, 59.96, 59.79, 54.99, 53.86, 41.85, 23.13, 22.16, 21.94, 20.43, 11.79.

Compound 10, ^1H NMR (600 MHz, D_2O) δ 4.54 (d, *J* = 4.5 Hz, 1H), 4.49 (d, *J* = 8.4 Hz, 1H), 4.19 (t, *J* = 5.2 Hz, 1H), 4.06 – 4.01 (m, 1H), 3.86 (dd, *J* = 12.4, 1.7 Hz, 1H), 3.72 – 3.57 (m, 3H), 3.47 (dd, *J* = 10.2, 8.5 Hz, 1H), 3.44 – 3.35 (m, 2H), 1.99 (s, 3H), 1.95 (s, 3H). ^{13}C NMR (151 MHz, D_2O) δ 174.85, 174.57, 114.93, 101.41, 85.63, 83.64, 82.98, 79.23, 75.85, 73.42, 69.77, 60.58, 60.23, 55.44, 22.14, 22.03, 21.99.

II. Supplementary Figure

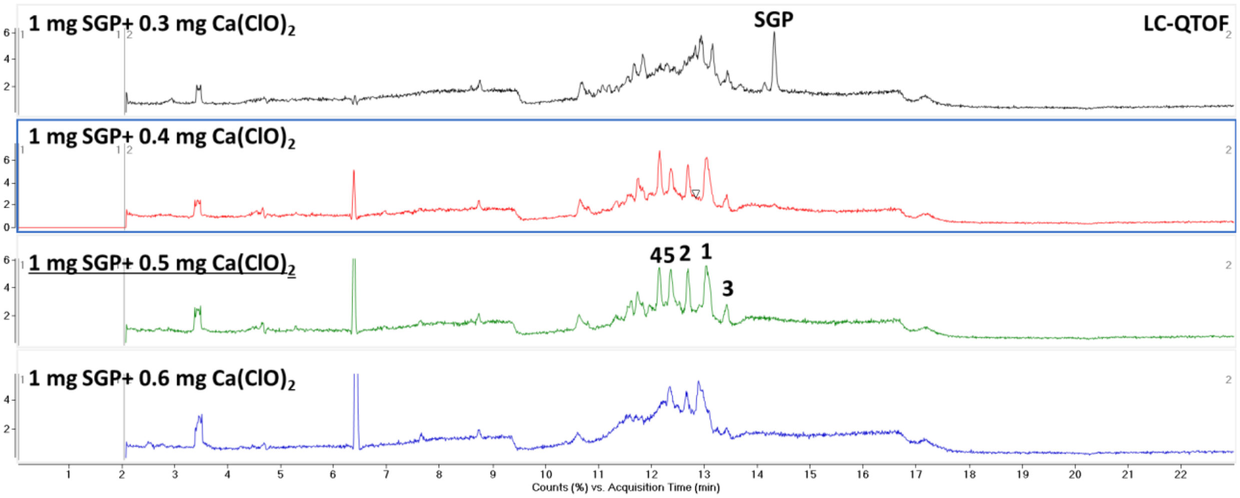

Figure S1. LC-MS profile of SGP reacted with different ratios of $Ca(ClO)_2$.

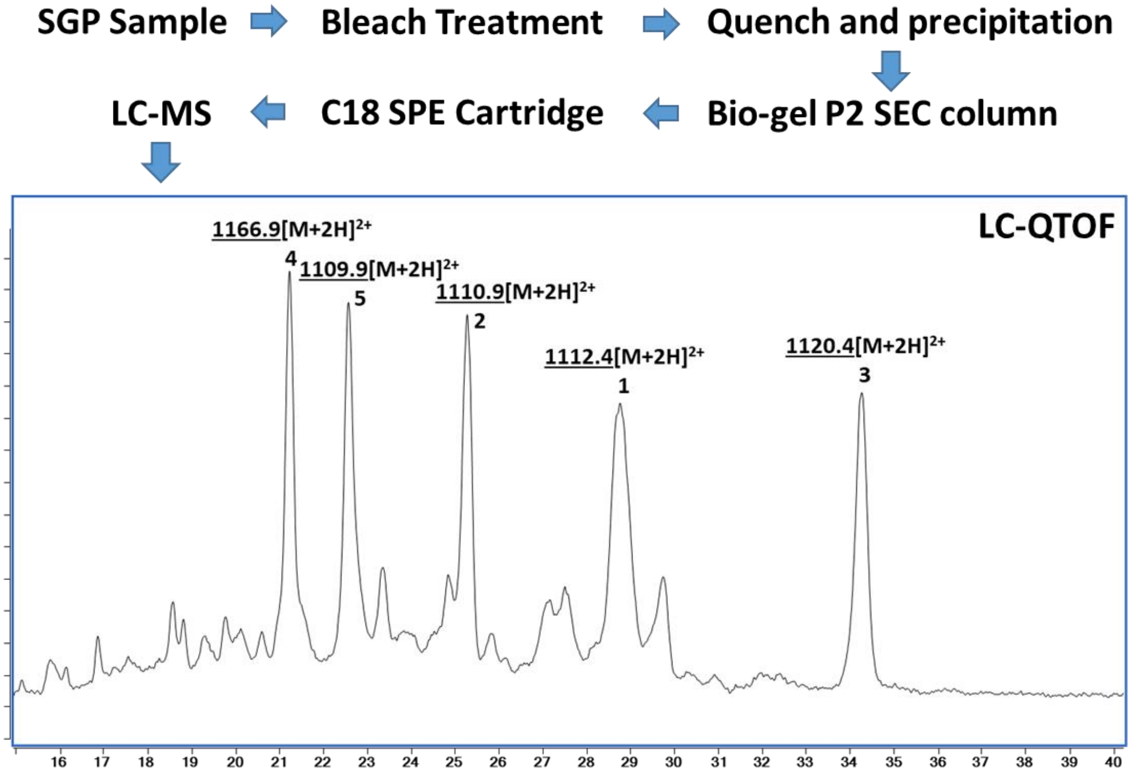

Figure S2. SGP LCMS profile generated by oxidative release.

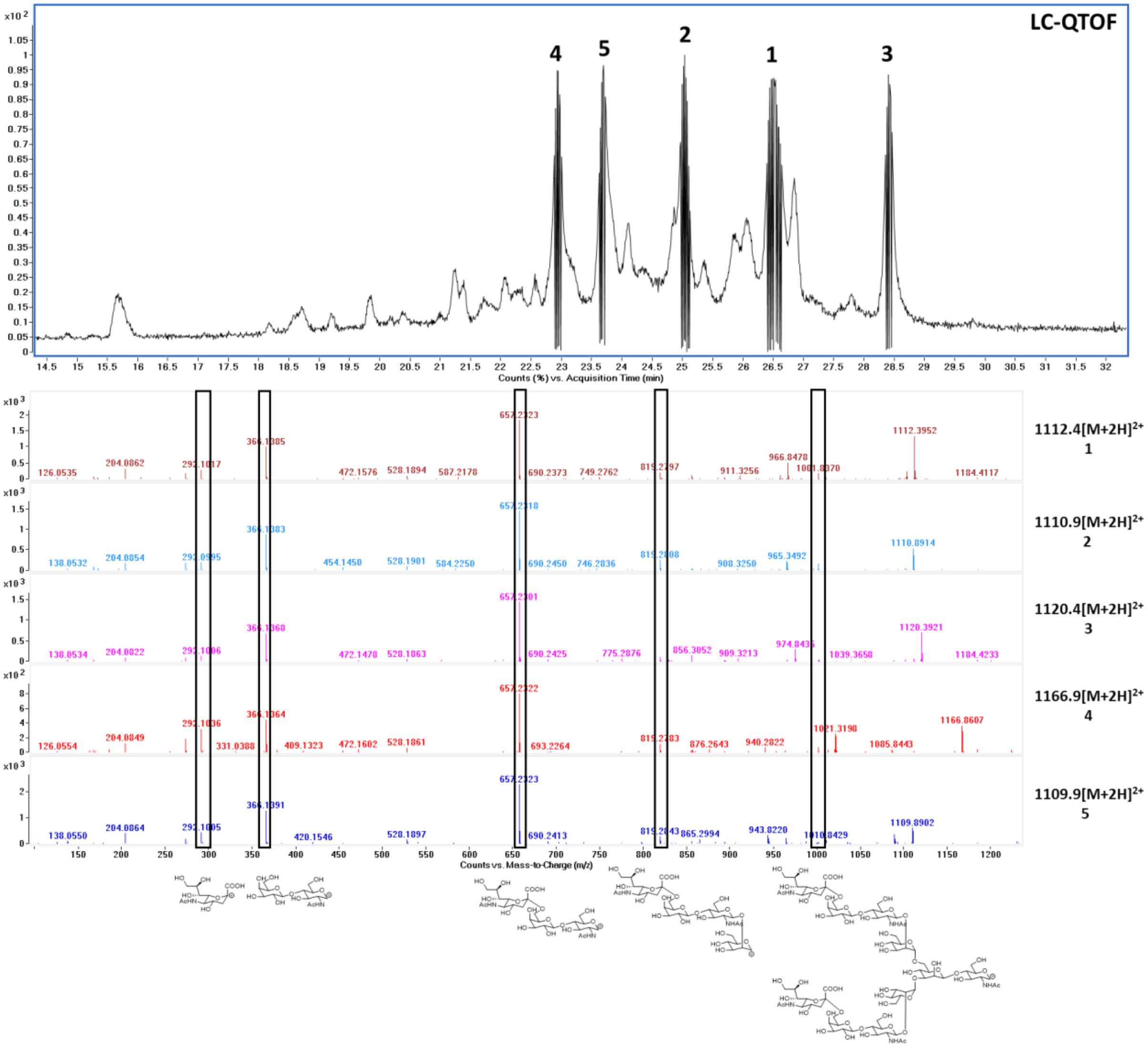

Figure S3. LC-MS/MS profiles of SGP 5 major products generated by bleach reaction.

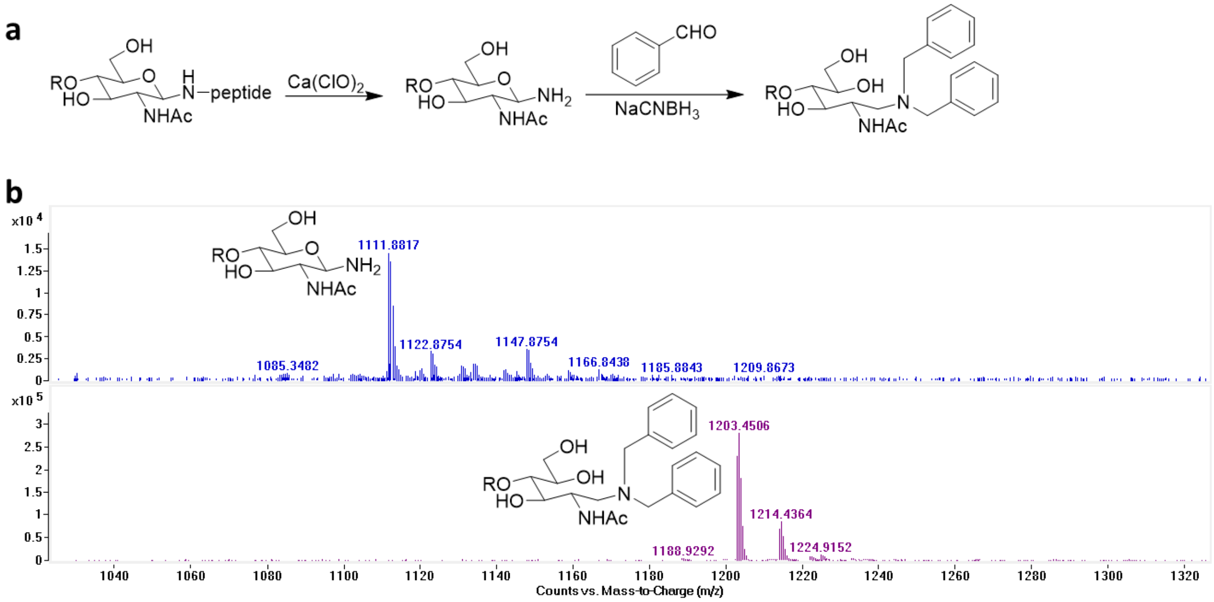

Figure S4. (**a**) Reaction scheme of benzaldehyde reduction amination of SGP bleach reaction mixture;(**b**) Mass spectrum data of glycosylamine before and after benzaldehyde reduction amination.

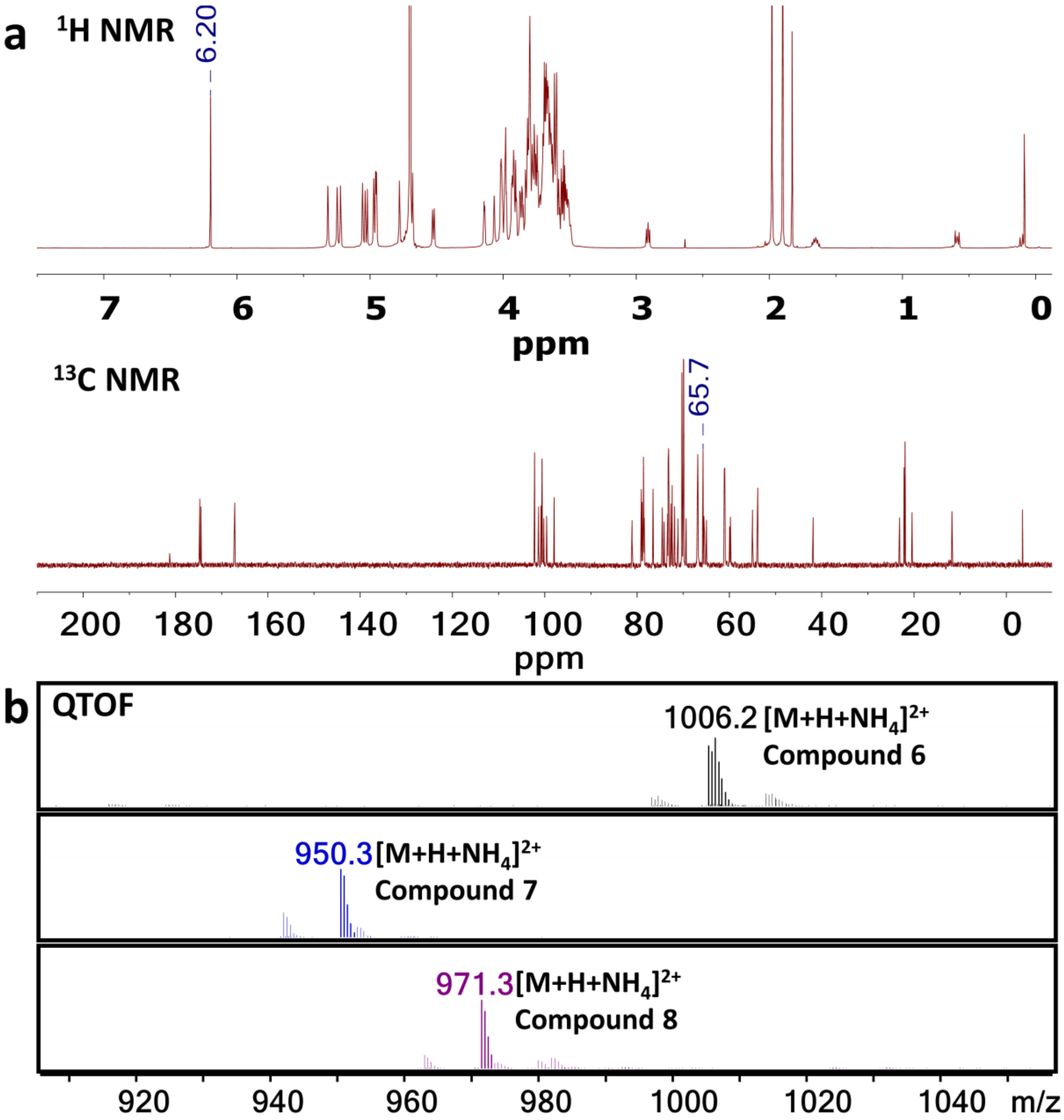

Figure S5. (**a**) ^{1}H and ^{13}C NMR spectroscopy data of $Man_9GlcNAc_2$-DCA; (**b**) MS profile of $Man_9GlcNAc_2$-DCA, $Man_9GlcNAc_2$-NH_2 and $Man_9GlcNAc_2$-NHAc.

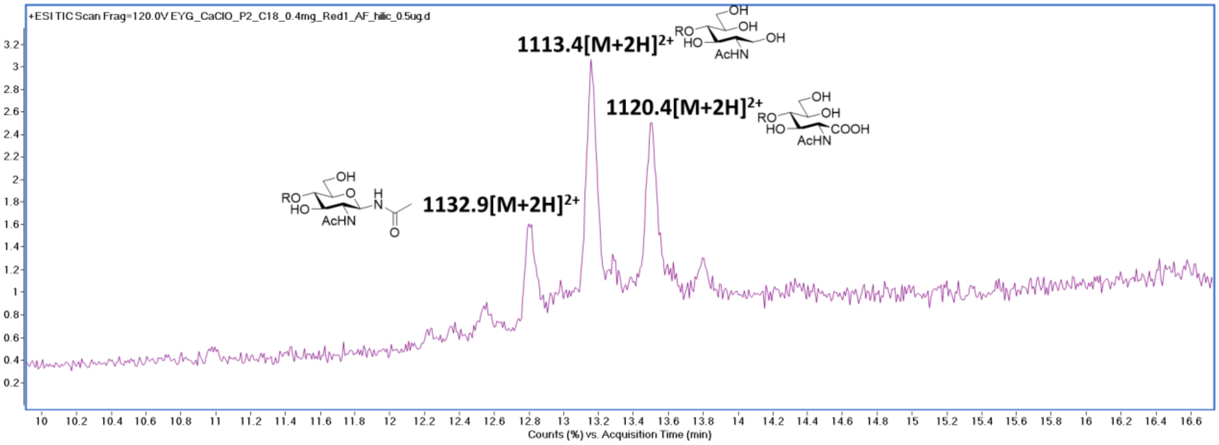

Figure S6. LC-MS profile of SGP Bleach reaction mixture with Pd/C in 5 % ammonium formate aqueous solution.

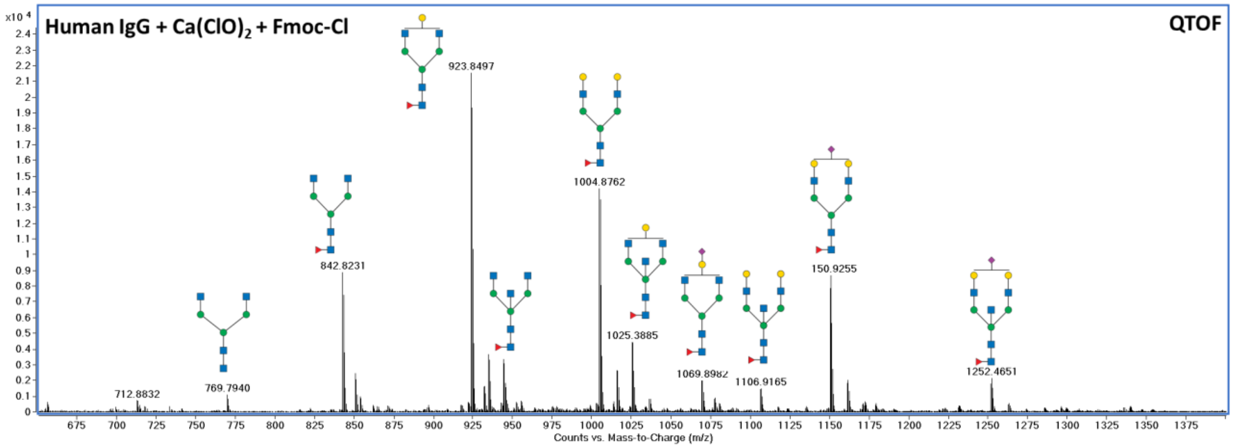

Figure S7. Fmoc-labeling N-glycans MS profile of Human IgG treated with $Ca(ClO)_2$.

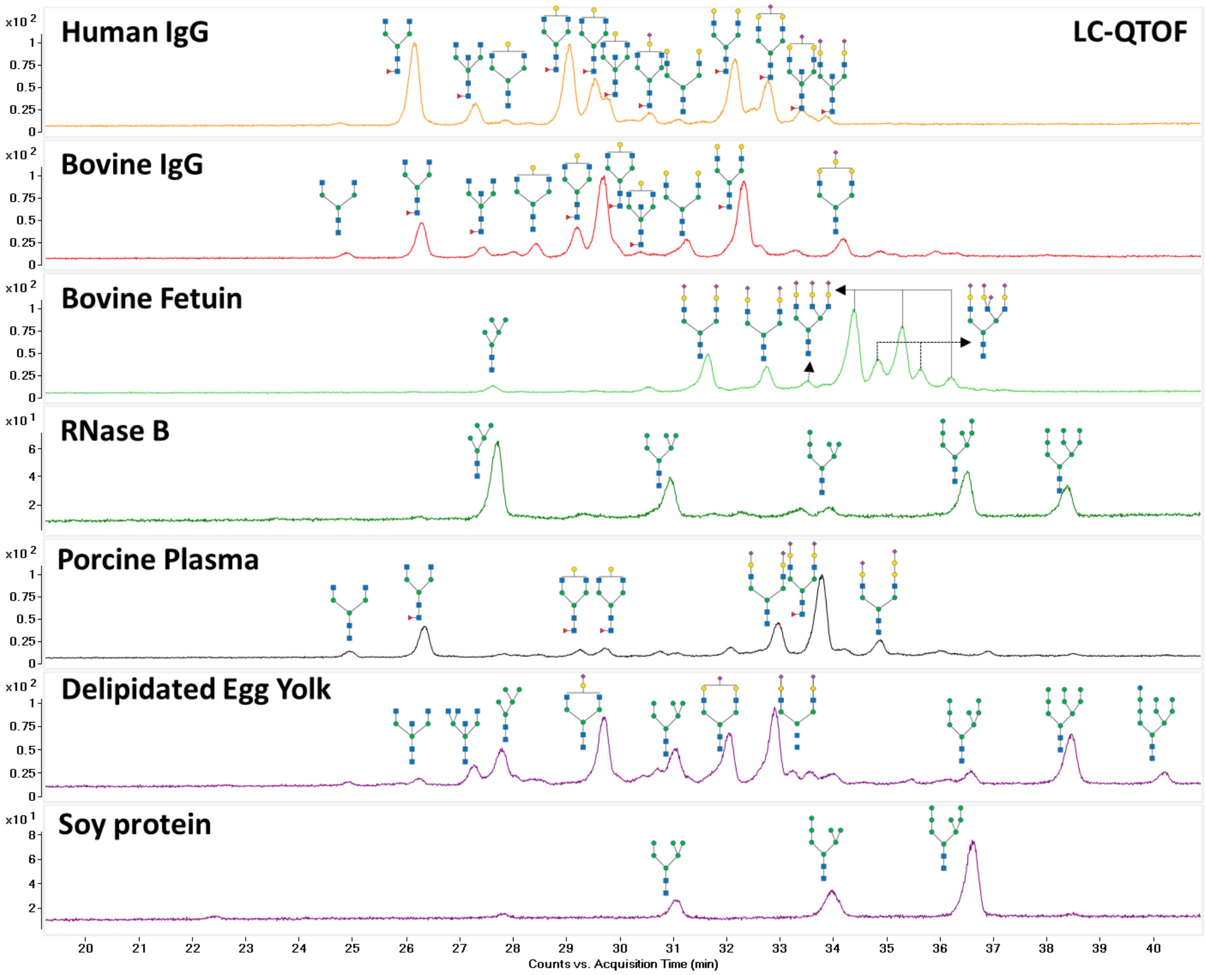

Figure S8. N-glycan LC-MS profile generated by InstantPC protocol of human IgG, bovine IgG, bovine fetuin, RNase B, porcine plasma, delipidated egg yolk and soy protein.

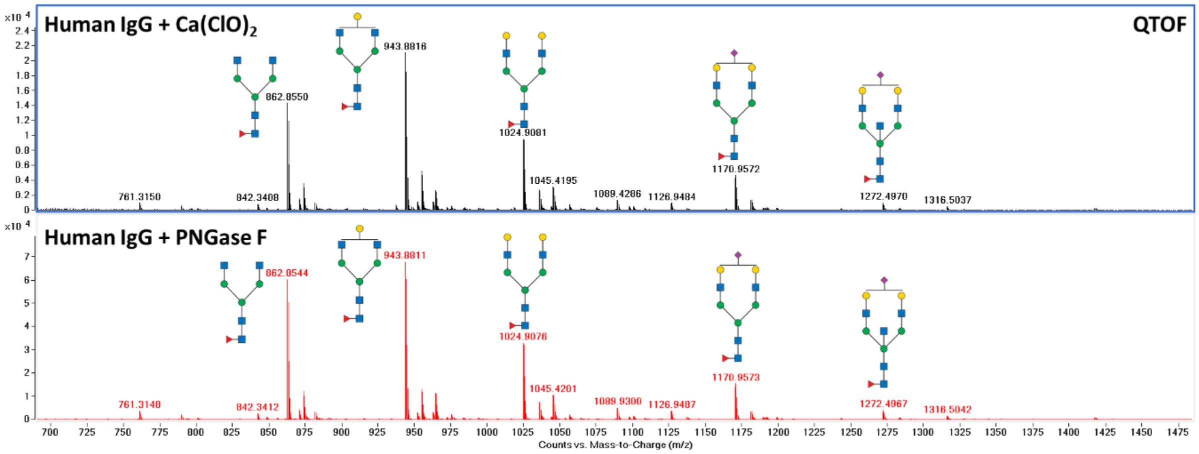

Figure S9. Procaine-labeling N-glycans MS profile of Human IgG treated with $Ca(ClO)_2$ and PNGase.

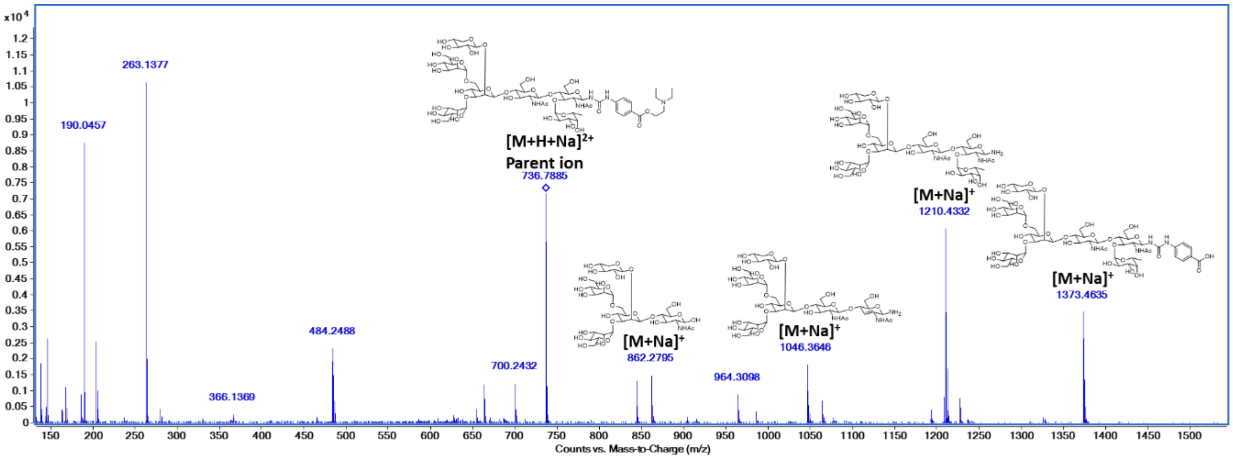

Figure S10. MS/MS profile of core α3-fucosylated N-glycans generated by oxidative release and specific labeling protocol of soy protein.

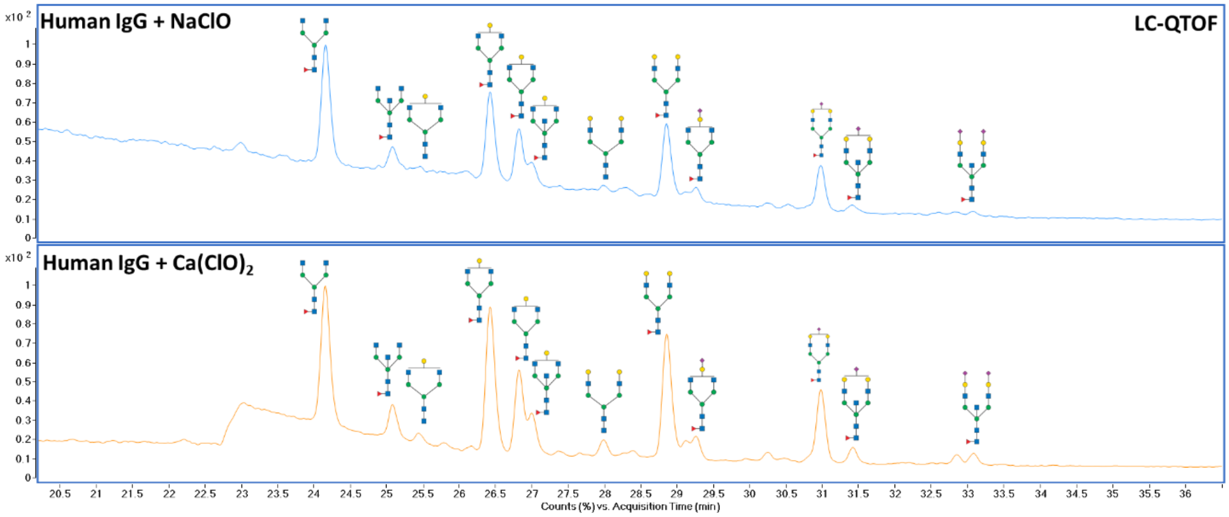

Figure S11. N-glycan LC-MS profile generated by NaClO or $Ca(ClO)_2$ oxidative release and specific labeling protocol of human IgG.

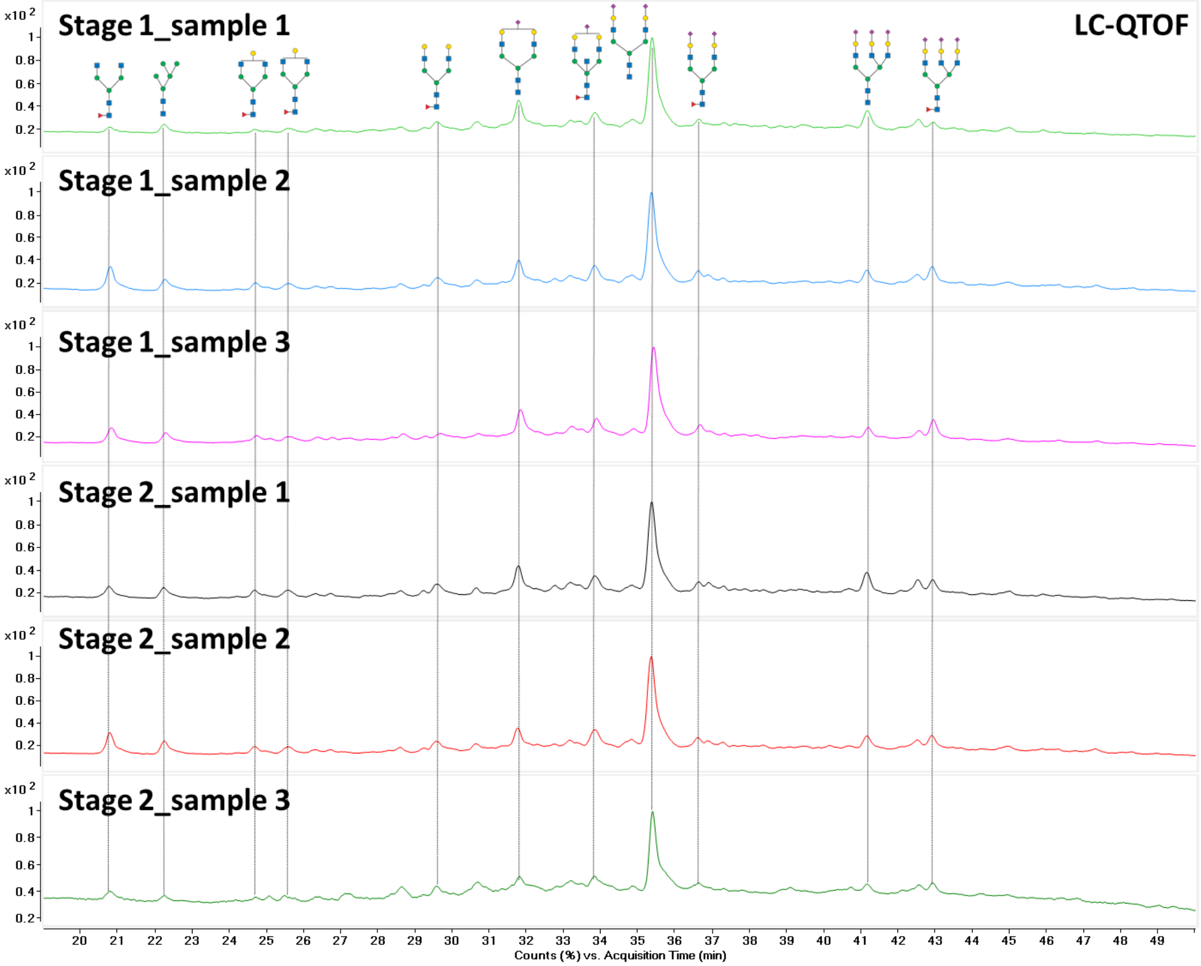

Figure S12. N-glycan LC-MS profile of human lung cancer serum stage 1&2 samples generated by N-glycan oxidative release and specific labeling protocol.

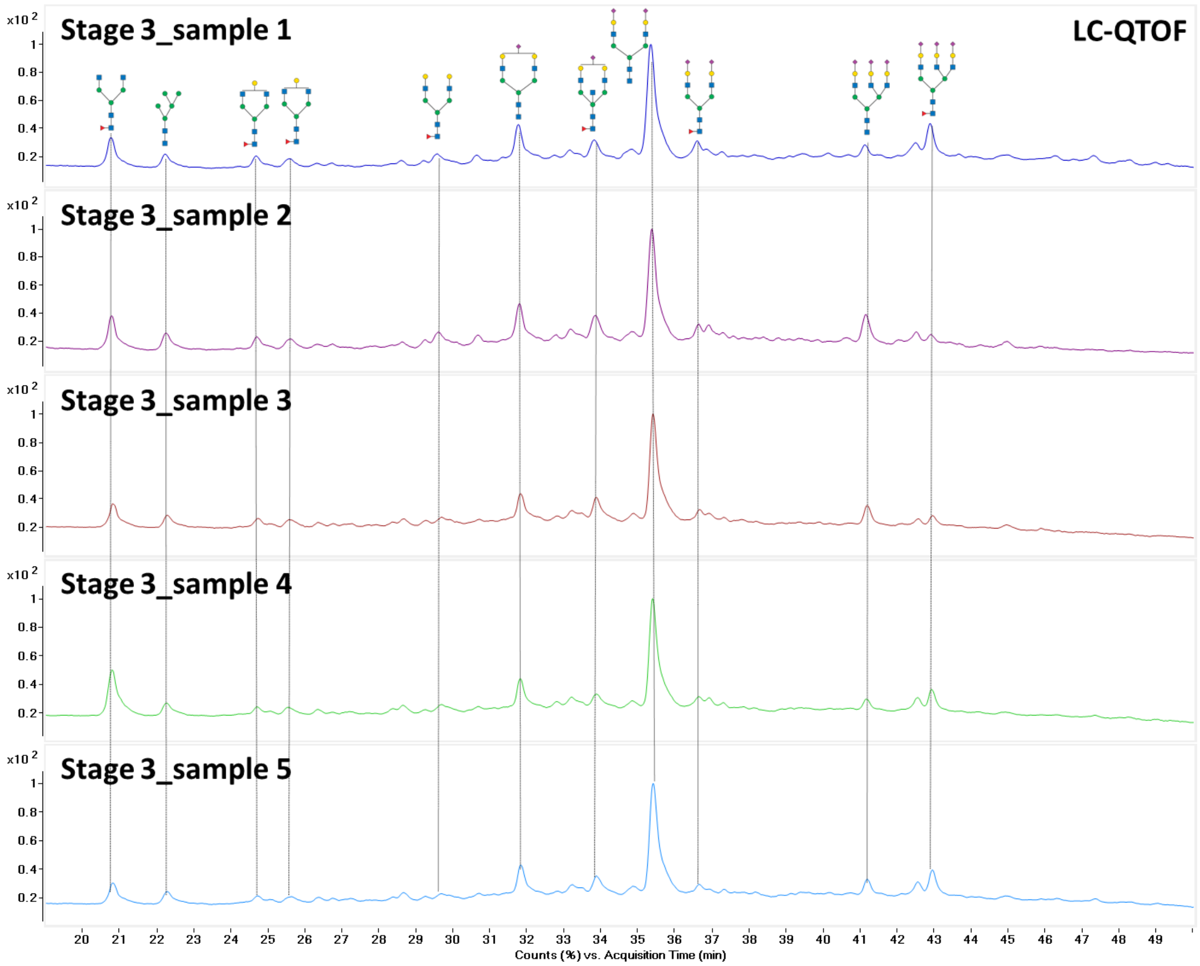

Figure S13. N-glycan LC-MS profile of human lung cancer serum stage 3 samples generated by N-glycan oxidative release and specific labeling protocol.

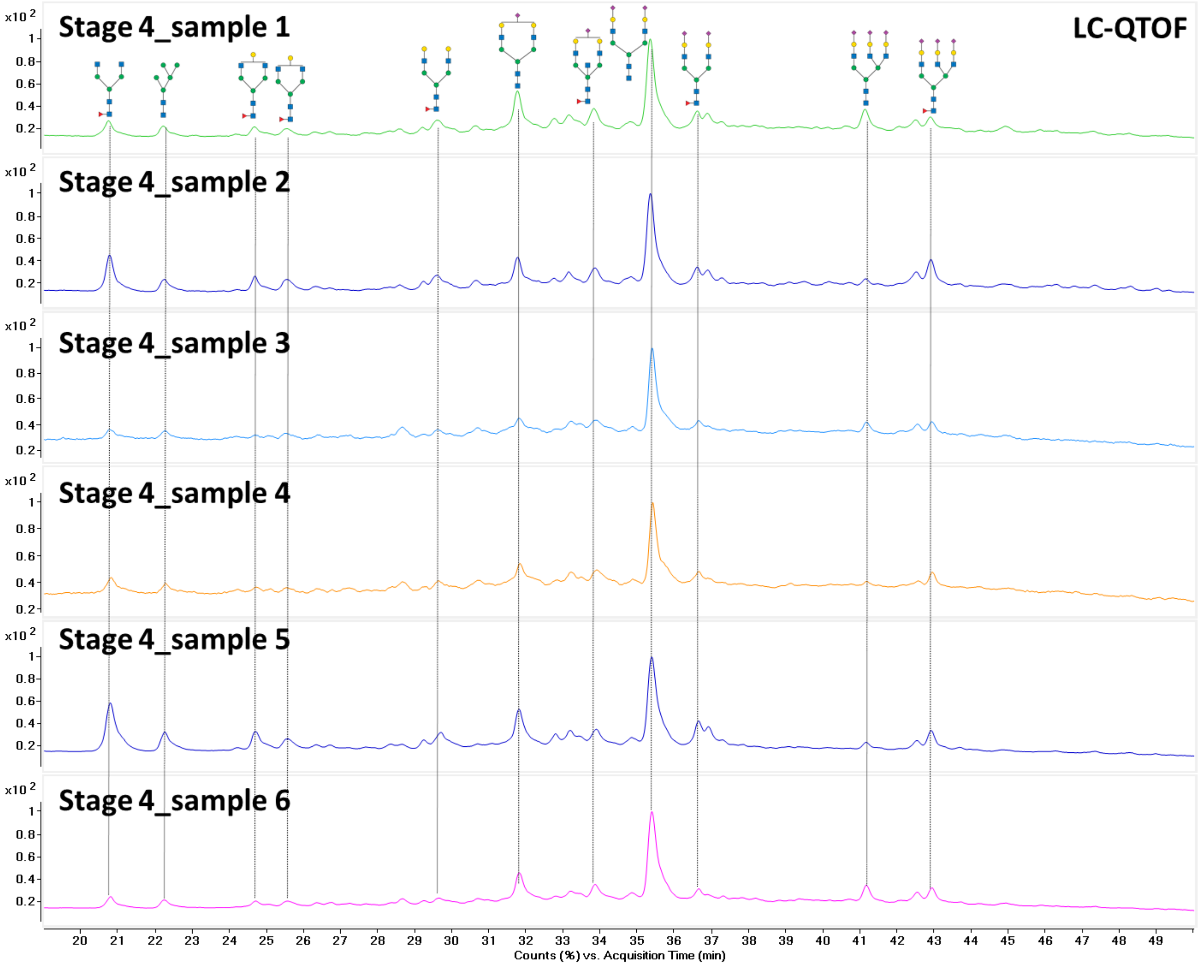

Figure S14. N-glycan LCMS profile of human lung cancer serum stage 4 samples generated by N-glycan bleach release and specific labeling protocol.

III. Supplementary Table

Table S1. Normal human serum pooled samples N-glycan composition and totol ion counts based on LC-MS profile.

					NHS-1		NHS-2		NHS-3		NHS average	
Glycan#	composition	observed Mass [M+2H]2+	Calc. Mass	Retention	Abund	% abund	Abund	% abund	Abund	% abund	% abund	STDEV
Glycan#1	H3N5	891.3651	1780.7	20.689	17950	0.32%	16198	0.34%	17277	0.39%	0.35%	0.03%
Glycan#2	H3N4F1	862.8549	1723.7	20.885	328675	5.95%	328129	6.87%	218327	4.89%	5.90%	0.99%
Glycan#3	H5N2	748.7996	1495.58	22.32	133039	2.41%	112605	2.36%	82213	1.84%	2.20%	0.31%
Glycan#4	H4N2	667.7729	1333.53	22.324	21559	0.39%	14107	0.30%	12052	0.27%	0.32%	0.06%
Glycan#5	H3N5F1	964.394	1926.77	22.355	65428	1.18%	66728	1.40%	58480	1.31%	1.30%	0.11%
Glycan#6	H4N4	870.852	1739.69	23.102	15275	0.28%	15996	0.33%	16366	0.37%	0.33%	0.05%
Glycan#7	H4N5	972.392	1942.77	24.293	12396	0.22%	11738	0.25%	11594	0.26%	0.24%	0.02%
Glycan#8	H5N6F1	1227.9447	2453.87	24.456	12013	0.22%	3431	0.07%	3877	0.09%	0.13%	0.08%
Glycan#9	H4N4F1	943.8816	1885.75	24.773	188220	3.41%	185143	3.87%	164079	3.67%	3.65%	0.23%
Glycan#10	H5N6F1	1227.9443	2453.87	25.083	16984	0.31%	9050	0.19%	7337	0.16%	0.22%	0.08%
Glycan#11	H5N6F1	1227.9445	2453.87	25.488	22141	0.40%	8673	0.18%	9630	0.22%	0.27%	0.12%
Glycan#12	H4N4F1	943.8813	1885.75	25.576	101309	1.83%	106448	2.23%	90972	2.04%	2.03%	0.20%
Glycan#13	H4N5F1	1045.4204	2088.83	25.701	68574	1.24%	74622	1.56%	65909	1.47%	1.43%	0.17%
Glycan#14	H6N2	829.8252	1657.64	26.28	31972	0.58%	25020	0.52%	17627	0.39%	0.50%	0.09%
Glycan#15	H4N3S1	914.8596	1827.7	26.374	45734	0.83%	33632	0.70%	31386	0.70%	0.74%	0.07%
Glycan#16	H4N5F1	1045.4197	2088.82	26.436	13606	0.25%	14473	0.30%	13400	0.30%	0.28%	0.03%
Glycan#17	H6N2	829.8253	1657.63	26.795	60465	1.09%	28247	0.59%	22983	0.51%	0.73%	0.32%
Glycan#18	H5N4	951.8779	1901.74	27.732	37963	0.69%	43593	0.91%	40098	0.90%	0.83%	0.13%
Glycan#19	H4N3F1S1	987.8889	1973.76	28.182	12957	0.23%	11907	0.25%	9371	0.21%	0.23%	0.02%
Glycan#20	H4N4S1	1016.3989	2030.78	28.392	54243	0.98%	41825	0.88%	38271	0.86%	0.90%	0.07%
Glycan#21	H4N5F1S1	1190.9667	2379.92	29.26	8297	0.15%	6324	0.13%	7350	0.16%	0.15%	0.02%
Glycan#22	H5N4F1	1024.9074	2047.8	29.29	156051	2.82%	161498	3.38%	144672	3.24%	3.15%	0.29%
Glycan#23	H5N5F1	1126.446	2250.88	29.626	33307	0.60%	41379	0.87%	32939	0.74%	0.74%	0.13%
Glycan#24	H5N3S1	995.8861	1989.76	29.694	54595	0.99%	47155	0.99%	37230	0.83%	0.94%	0.09%
Glycan#25	H4N5S1	1117.9387	2233.86	29.733	45929	0.83%	32020	0.67%	33385	0.75%	0.75%	0.08%
Glycan#26	H4N4F1S1	1089.4281	2176.84	29.94	35156	0.64%	32139	0.67%	31543	0.71%	0.67%	0.03%
Glycan#27	H7N2	910.8511	1819.69	31.013	9226	0.17%	7248	0.15%	6620	0.15%	0.16%	0.01%
Glycan#28	H4N5F1S1	1190.9684	2379.92	31.126	15205	0.28%	15670	0.33%	13131	0.29%	0.30%	0.03%
Glycan#29	H5N4S1	1097.4254	2192.84	31.35	38028	0.69%	67049	1.40%	68900	1.54%	1.21%	0.46%
Glycan#30	H5N4F1S1	1170.4535	2338.89	31.548	34780	0.63%	30838	0.65%	27274	0.61%	0.63%	0.02%
Glycan#31	H5N4S1	1097.4236	2192.83	31.81	595912	10.78%	480651	10.06%	472959	10.58%	10.48%	0.38%
Glycan#32	H5N6F3	1374.0383	2746.06	31.833	28844	0.52%	16032	0.34%	17256	0.39%	0.41%	0.10%
Glycan#33	H5N5S1	1198.965	2395.92	32.788	82681	1.50%	61335	1.28%	56657	1.27%	1.35%	0.13%
Glycan#34	H5N4F1S1	1170.4553	2338.9	33.196	217728	3.94%	203788	4.26%	184180	4.12%	4.11%	0.16%
Glycan#35	H3N6S1	1138.4108	2274.81	33.217	9475	0.17%	7035	0.15%	8490	0.19%	0.17%	0.02%
Glycan#36	H4N5F2S1	1263.9777	2525.94	33.322	22154	0.40%	8990	0.19%	9699	0.22%	0.27%	0.12%
Glycan#37	H6N3S1	1076.9116	2151.8	33.377	25189	0.46%	19401	0.41%	18526	0.41%	0.43%	0.03%
Glycan#38	H5N4F1S2	1316.0013	2629.9	33.469	39875	0.72%	32267	0.68%	32286	0.72%	0.71%	0.03%
Glycan#39	H5N4S2	1242.9738	2483.93	33.825	194409	3.52%	162227	3.39%	152575	3.41%	3.44%	0.07%
Glycan#40	H5N5F1S1	1271.9944	2541.97	33.918	139364	2.52%	130337	2.73%	126152	2.82%	2.69%	0.15%
Glycan#41	H5N4S2	1242.9733	2483.93	34.79	94458	1.71%	83182	1.74%	61996	1.39%	1.61%	0.20%
Glycan#42	H5N4F1S2	1316.0026	2629.99	34.972	26406	0.48%	26582	0.56%	23643	0.53%	0.52%	0.04%
Glycan#43	H5N4S2	1242.9711	2483.93	35.373	1731549	31.34%	1375655	28.78%	1391789	31.15%	30.42%	1.42%
Glycan#44	H6N5S1	1280.0163	2558.02	35.791	28818	0.52%	38348	0.80%	42206	0.94%	0.76%	0.22%
Glycan#45	H6N5S1	1280.0076	2558	36.295	17139	0.31%	18466	0.39%	19981	0.45%	0.38%	0.07%
Glycan#46	H5N4F1S2	1316.0028	2629.99	36.629	212957	3.85%	205948	4.31%	183437	4.11%	4.09%	0.23%
Glycan#47	H5N5F1S2	1417.5411	2833.07	36.9	82216	1.49%	75383	1.58%	73320	1.64%	1.57%	0.08%
Glycan#48	H6N5S1	1279.9924	2557.97	37.241	17210	0.31%	12354	0.26%	12005	0.27%	0.28%	0.03%
Glycan#49	H9N2	1072.9035	2143.79	37.582	19235	0.35%	13654	0.29%	14562	0.33%	0.32%	0.03%
Glycan#50	H6N5S2	1425.538	2849.06	38.189	21669	0.39%	21443	0.45%	22631	0.51%	0.45%	0.06%
Glycan#51	H6N5S3	1571.084	3140.13	39.822	10601	0.19%	7307	0.15%	8510	0.19%	0.18%	0.02%
Glycan#52	H6N5F1S2	1498.568	2995.12	40.15	3292	0.06%	5438	0.11%	4510	0.10%	0.09%	0.03%
Glycan#53	H6N5S3	1571.0865	3140.15	41.134	97029	1.76%	80445	1.68%	79421	1.78%	1.74%	0.05%
Glycan#54	H6N5F1S2	1498.5671	2995.12	41.475	2037	0.04%	2084	0.04%	2405	0.05%	0.04%	0.01%
Glycan#55	H6N5S3	1571.0866	3140.16	42.49	52608	0.95%	44228	0.93%	39932	0.89%	0.92%	0.03%
Glycan#56	H6N5F1S3	1644.1149	3286.22	42.885	44320	0.80%	27310	0.57%	26755	0.60%	0.66%	0.13%
Glycan#57	H6N5F2S3	1145.1000([M+3H]3+)	3432.27	43.734	4282	0.08%	4504	0.09%	4611	0.10%	0.09%	0.01%
Glycan#58	H6N7F1S1	1556.0836	3110.15	45.02	1563	0.03%	5645	0.12%	17878	0.40%	0.18%	0.19%
Glycan#59	H6N7F1S1	1556.0834	3110.15	46.311	820	0.01%	2754	0.06%	8107	0.18%	0.08%	0.09%
Glycan#60	H7N6F1S4	1315.1621([M+3H]3+)	3942.47	46.342	2805	0.05%	3274	0.07%	2548	0.06%	0.06%	0.01%
Glycan#61	H6N7F2S1	1629.1117	3256.21	46.785	822	0.01%	3875	0.08%	7613	0.17%	0.09%	0.08%
Glycan#62	H7N6F1S4	1315.1621([M+3H]3+)	3942.47	47.173	2646	0.05%	2316	0.05%	3709	0.08%	0.06%	0.02%
Glycan#63	H7N6F2S4	1363.8442([M+3H]3+)	4088.51	47.44	1258	0.02%	1337	0.03%	1005	0.02%	0.02%	0.00%
Glycan#64	H7N6F1S4	1315.1621([M+3H]3+)	3942.47	48.024	1483	0.03%	1210	0.03%	891	0.02%	0.02%	0.00%
Glycan#65	H7N6F2S4	1363.8442([M+3H]3+)	4088.51	48.299	867	0.02%	1063	0.02%	1200	0.03%	0.02%	0.01%
Glycan#66	H7N6F3S4	1412.5293([M+3H]3+)	4234.57	49.02	693	0.01%	681	0.01%	701	0.02%	0.01%	0.00%
	total ion count				5525491	100.00%	4779434	100.00%	4468439	100.00%	100.00%	

Table S2. Human lung cancer serum stage 1 samples N-glycan composition and totol ion counts based on LC-MS profile

					HLCS1-1		HLCS1-2		HLCS1-3		STAGE 1 average	
Glycan#	composition	observed Mass [M+2H]2+	Calc. Mass	Retention	Abund	% abund	Abund	% abund	Abund	% abund	% abund	STDEV
Glycan#1	H3N5	891.3651	1780.7	20.689	5089	0.12%	35469	0.57%	32813	0.40%	0.36%	0.23%
Glycan#2	H3N4F1	862.8549	1723.7	20.885	110681	2.51%	659141	10.59%	584804	7.18%	6.76%	4.06%
Glycan#3	H5N2	748.7996	1495.58	22.32	96198	2.18%	87711	1.41%	185042	2.27%	1.95%	0.47%
Glycan#4	H4N2	667.7729	1333.53	22.324	14905	0.34%	9598	0.15%	28613	0.35%	0.28%	0.11%
Glycan#5	H3N5F1	964.394	1926.77	22.355	59446	1.35%	160438	2.58%	121809	1.49%	1.81%	0.67%
Glycan#6	H4N4	870.852	1739.69	23.102	12827	0.29%	7975	0.13%	22070	0.27%	0.23%	0.09%
Glycan#7	H4N5	972.392	1942.77	24.293	7689	0.17%	16801	0.27%	15113	0.19%	0.21%	0.05%
Glycan#8	H5N6F1	1227.9447	2453.87	24.456	4902	0.11%	5485	0.09%	30255	0.37%	0.19%	0.16%
Glycan#9	H4N4F1	943.8816	1885.75	24.773	66529	1.51%	158134	2.54%	192718	2.36%	2.14%	0.55%
Glycan#10	H5N6F1	1227.9443	2453.87	25.083	10911	0.25%	11515	0.18%	41203	0.51%	0.31%	0.17%
Glycan#11	H5N6F1	1227.9445	2453.87	25.488	12581	0.29%	13235	0.21%	60392	0.74%	0.41%	0.29%
Glycan#12	H4N4F1	943.8813	1885.75	25.576	42554	0.96%	90589	1.46%	87325	1.07%	1.16%	0.26%
Glycan#13	H4N5F1	1045.4204	2088.83	25.701	49800	1.13%	92040	1.48%	91733	1.13%	1.24%	0.20%
Glycan#14	H6N2	829.8252	1657.64	26.28	20419	0.46%	19640	0.32%	34132	0.42%	0.40%	0.08%
Glycan#15	H4N3S1	914.8596	1827.7	26.374	46992	1.07%	49355	0.79%	87262	1.07%	0.98%	0.16%
Glycan#16	H4N5F1	1045.4197	2088.82	26.436	9857	0.22%	22279	0.36%	17643	0.22%	0.27%	0.08%
Glycan#17	H6N2	829.8253	1657.63	26.795	23079	0.52%	30152	0.48%	58483	0.72%	0.58%	0.12%
Glycan#18	H5N4	951.8779	1901.74	27.732	40720	0.92%	31350	0.50%	71379	0.88%	0.77%	0.23%
Glycan#19	H4N3F1S1	987.8889	1973.76	28.182	7698	0.17%	11670	0.19%	35062	0.43%	0.26%	0.14%
Glycan#20	H4N4S1	1016.3989	2030.78	28.392	53375	1.21%	32417	0.52%	92886	1.14%	0.96%	0.38%
Glycan#21	H4N5F1S1	1190.9667	2379.92	29.26	4439	0.10%	12996	0.21%	11436	0.14%	0.15%	0.05%
Glycan#22	H5N4F1	1024.9074	2047.8	29.29	64149	1.45%	66254	1.06%	106327	1.30%	1.27%	0.20%
Glycan#23	H5N5F1	1126.446	2250.88	29.626	25771	0.58%	44463	0.71%	42143	0.52%	0.61%	0.10%
Glycan#24	H5N3S1	995.8861	1989.76	29.694	54351	1.23%	56777	0.91%	57987	0.71%	0.95%	0.26%
Glycan#25	H4N5S1	1117.9387	2233.86	29.733	5682	0.13%	65154	1.05%	60770	0.75%	0.64%	0.47%
Glycan#26	H4N4F1S1	1089.4281	2176.84	29.94	13767	0.31%	25833	0.41%	23277	0.29%	0.34%	0.07%
Glycan#27	H7N2	910.8511	1819.69	31.013	3773	0.09%	4896	0.08%	9704	0.12%	0.09%	0.02%
Glycan#28	H4N5F1S1	1190.9684	2379.92	31.126	6483	0.15%	29672	0.48%	36960	0.45%	0.36%	0.18%
Glycan#29	H5N4S1	1097.4254	2192.84	31.35	65219	1.48%	78501	1.26%	66852	0.82%	1.19%	0.34%
Glycan#30	H5N4F1S1	1170.4535	2338.89	31.548	53685	1.22%	32757	0.53%	90095	1.11%	0.95%	0.37%
Glycan#31	H5N4S1	1097.4236	2192.83	31.81	507015	11.49%	533084	8.56%	811164	9.95%	10.00%	1.47%
Glycan#32	H5N6F3	1374.0383	2746.06	31.833	30446	0.69%	31794	0.51%	62629	0.77%	0.66%	0.13%
Glycan#33	H5N5S1	1198.965	2395.92	32.788	20035	0.45%	81291	1.31%	57231	0.70%	0.82%	0.44%
Glycan#34	H5N4F1S1	1170.4553	2338.9	33.196	138188	3.13%	134905	2.17%	231271	2.84%	2.71%	0.49%
Glycan#35	H3N6S1	1138.4108	2274.81	33.217	4635	0.11%	7533	0.12%	9310	0.11%	0.11%	0.01%
Glycan#36	H4N5F2S1	1263.9777	2525.94	33.322	15251	0.35%	16441	0.26%	43944	0.54%	0.38%	0.14%
Glycan#37	H6N3S1	1076.9116	2151.8	33.377	23870	0.54%	25928	0.42%	24113	0.30%	0.42%	0.12%
Glycan#38	H5N4F1S2	1316.0013	2629.9	33.469	69276	1.57%	38618	0.62%	106824	1.31%	1.17%	0.49%
Glycan#39	H5N4S2	1242.9738	2483.93	33.825	180084	4.08%	299138	4.81%	427850	5.25%	4.71%	0.59%
Glycan#40	H5N5F1S1	1271.9944	2541.97	33.918	80789	1.83%	140727	2.26%	168011	2.06%	2.05%	0.21%
Glycan#41	H5N4S2	1242.9733	2483.93	34.79	101684	2.31%	121087	1.95%	135470	1.66%	1.97%	0.32%
Glycan#42	H5N4F1S2	1316.0026	2629.99	34.972	29011	0.66%	29449	0.47%	42545	0.52%	0.55%	0.10%
Glycan#43	H5N4S2	1242.9711	2483.93	35.373	1675182	37.98%	1936902	31.11%	2590508	31.78%	33.63%	3.78%
Glycan#44	H6N5S1	1280.0163	2558.02	35.791	24008	0.54%	40593	0.65%	70128	0.86%	0.69%	0.16%
Glycan#45	H6N5S1	1280.0076	2558	36.295	23432	0.53%	22060	0.35%	27950	0.34%	0.41%	0.11%
Glycan#46	H5N4F1S2	1316.0028	2629.99	36.629	148790	3.37%	301311	4.84%	383361	4.70%	4.31%	0.81%
Glycan#47	H5N5F1S2	1417.5411	2833.07	36.9	29091	0.66%	88272	1.42%	72274	0.89%	0.99%	0.39%
Glycan#48	H6N5S1	1279.9924	2557.97	37.241	22025	0.50%	21506	0.35%	25104	0.31%	0.38%	0.10%
Glycan#49	H9N2	1072.9035	2143.79	37.582	17376	0.39%	19471	0.31%	28645	0.35%	0.35%	0.04%
Glycan#50	H6N5S2	1425.538	2849.06	38.189	24122	0.55%	26425	0.42%	34031	0.42%	0.46%	0.07%
Glycan#51	H6N5S3	1571.084	3140.13	39.822	8929	0.20%	10996	0.18%	15415	0.19%	0.19%	0.01%
Glycan#52	H6N5F1S2	1498.568	2995.12	40.15	2793	0.06%	8717	0.14%	14978	0.18%	0.13%	0.06%
Glycan#53	H6N5S3	1571.0865	3140.15	41.134	115311	2.61%	106634	1.71%	115648	1.42%	1.92%	0.62%
Glycan#54	H6N5F1S2	1498.5671	2995.12	41.475	1668	0.04%	4518	0.07%	3328	0.04%	0.05%	0.02%
Glycan#55	H6N5S3	1571.0866	3140.16	42.49	65076	1.48%	75063	1.21%	84045	1.03%	1.24%	0.22%
Glycan#56	H6N5F1S3	1644.1149	3286.22	42.885	20150	0.46%	68383	1.10%	99966	1.23%	0.93%	0.41%
Glycan#57	H6N5F2S3	1145.1000([M+3H]3+)	3432.27	43.734	4006	0.09%	13080	0.21%	17331	0.21%	0.17%	0.07%
Glycan#58	H6N7F1S1	1556.0836	3110.15	45.02	15567	0.35%	9616	0.15%	1234	0.02%	0.17%	0.17%
Glycan#59	H6N7F1S1	1556.0834	3110.15	46.311	6676	0.15%	6823	0.11%	1244	0.02%	0.09%	0.07%
Glycan#60	H7N6F1S4	1315.1621([M+3H]3+)	3942.47	46.342	3350	0.08%	7651	0.12%	13897	0.17%	0.12%	0.05%
Glycan#61	H6N7F2S1	1629.1117	3256.21	46.785	3264	0.07%	12939	0.21%	1163	0.01%	0.10%	0.10%
Glycan#62	H7N6F1S4	1315.1621([M+3H]3+)	3942.47	47.173	2547	0.06%	6492	0.10%	12044	0.15%	0.10%	0.05%
Glycan#63	H7N6F2S4	1363.8442([M+3H]3+)	4088.51	47.44	1033	0.02%	4063	0.07%	7375	0.09%	0.06%	0.03%
Glycan#64	H7N6F1S4	1315.1621([M+3H]3+)	3942.47	48.024	1177	0.03%	4639	0.07%	3944	0.05%	0.05%	0.02%
Glycan#65	H7N6F2S4	1363.8442([M+3H]3+)	4088.51	48.299	620	0.01%	3952	0.06%	5820	0.07%	0.05%	0.03%
Glycan#66	H7N6F3S4	1412.5293([M+3H]3+)	4234.57	49.02	1052	0.02%	2586	0.04%	4237	0.05%	0.04%	0.01%
	total ion count				4411100	100.00%	6224984	100.00%	8150320	100.00%	100.00%	

Table S3. Human lung cancer serum stage 2 samples N-glycan composition and totol ion counts based on LC-MS profile.

					HLCS2-1		HLCS2-2		HLCS2-3		STAGE 2 average	
Glycan#	composition	observed Mass [M+2H]2+	Calc. Mass	Retention	Abund	% abund	Abund	% abund	Abund	% abund	% abund	STDEV
Glycan#1	H3N5	891.3651	1780.7	20.689	27953	0.58%	24328	0.34%	6603	0.32%	0.41%	0.15%
Glycan#2	H3N4F1	862.8549	1723.7	20.885	200907	4.17%	639989	8.91%	91983	4.41%	5.83%	2.67%
Glycan#3	H5N2	748.7996	1495.58	22.32	98067	2.04%	130317	1.81%	28339	1.36%	1.74%	0.35%
Glycan#4	H4N2	667.7729	1333.53	22.324	13136	0.27%	17605	0.25%	2442	0.12%	0.21%	0.08%
Glycan#5	H3N5F1	964.394	1926.77	22.355	93744	1.95%	185612	2.58%	29602	1.42%	1.98%	0.58%
Glycan#6	H4N4	870.852	1739.69	23.102	12066	0.25%	9692	0.13%	5783	0.28%	0.22%	0.08%
Glycan#7	H4N5	972.392	1942.77	24.293	20919	0.43%	15201	0.21%	3902	0.19%	0.28%	0.14%
Glycan#8	H5N6F1	1227.9447	2453.87	24.456	3865	0.08%	5906	0.08%	17387	0.83%	0.33%	0.43%
Glycan#9	H4N4F1	943.8816	1885.75	24.773	131656	2.73%	172669	2.40%	43029	2.06%	2.40%	0.34%
Glycan#10	H5N6F1	1227.9443	2453.87	25.083	8847	0.18%	14010	0.20%	33894	1.63%	0.67%	0.83%
Glycan#11	H5N6F1	1227.9445	2453.87	25.488	10481	0.22%	12949	0.18%	48473	2.32%	0.91%	1.23%
Glycan#12	H4N4F1	943.8813	1885.75	25.576	65950	1.37%	91685	1.28%	22764	1.09%	1.25%	0.14%
Glycan#13	H4N5F1	1045.4204	2088.83	25.701	90611	1.88%	116138	1.62%	18102	0.87%	1.46%	0.53%
Glycan#14	H6N2	829.8252	1657.64	26.28	15338	0.32%	32490	0.45%	2069	0.10%	0.29%	0.18%
Glycan#15	H4N3S1	914.8596	1827.7	26.374	51970	1.08%	58485	0.81%	24461	1.17%	1.02%	0.19%
Glycan#16	H4N5F1	1045.4197	2088.82	26.436	15444	0.32%	21537	0.30%	5600	0.27%	0.30%	0.03%
Glycan#17	H6N2	829.8253	1657.63	26.795	20729	0.43%	42114	0.59%	12841	0.62%	0.54%	0.10%
Glycan#18	H5N4	951.8779	1901.74	27.732	47119	0.98%	32594	0.45%	6842	0.33%	0.59%	0.35%
Glycan#19	H4N3F1S1	987.8889	1973.76	28.182	7087	0.15%	25719	0.36%	3837	0.18%	0.23%	0.11%
Glycan#20	H4N4S1	1016.3989	2030.78	28.392	51125	1.06%	43352	0.60%	27056	1.30%	0.99%	0.35%
Glycan#21	H4N5F1S1	1190.9667	2379.92	29.26	11265	0.23%	16149	0.22%	3592	0.17%	0.21%	0.03%
Glycan#22	H5N4F1	1024.9074	2047.8	29.29	91835	1.91%	83822	1.17%	22963	1.10%	1.39%	0.45%
Glycan#23	H5N5F1	1126.446	2250.88	29.626	35284	0.73%	49964	0.70%	6967	0.33%	0.59%	0.22%
Glycan#24	H5N3S1	995.8861	1989.76	29.694	65008	1.35%	64557	0.90%	10585	0.51%	0.92%	0.42%
Glycan#25	H4N5S1	1117.9387	2233.86	29.733	61507	1.28%	36414	0.51%	10317	0.49%	0.76%	0.45%
Glycan#26	H4N4F1S1	1089.4281	2176.84	29.94	21055	0.44%	29326	0.41%	8988	0.43%	0.43%	0.02%
Glycan#27	H7N2	910.8511	1819.69	31.013	6972	0.14%	8187	0.11%	2408	0.12%	0.12%	0.02%
Glycan#28	H4N5F1S1	1190.9684	2379.92	31.126	17574	0.36%	35513	0.49%	5173	0.25%	0.37%	0.12%
Glycan#29	H5N4S1	1097.4254	2192.84	31.35	55453	1.15%	83794	1.17%	20004	0.96%	1.09%	0.12%
Glycan#30	H5N4F1S1	1170.4535	2338.89	31.548	45095	0.94%	35122	0.49%	12761	0.61%	0.68%	0.23%
Glycan#31	H5N4S1	1097.4236	2192.83	31.81	493587	10.25%	475092	6.61%	127146	6.10%	7.65%	2.26%
Glycan#32	H5N6F3	1374.0383	2746.06	31.833	22816	0.47%	35988	0.50%	18368	0.88%	0.62%	0.23%
Glycan#33	H5N5S1	1198.965	2395.92	32.788	106013	2.20%	60627	0.84%	10902	0.52%	1.19%	0.89%
Glycan#34	H5N4F1S1	1170.4553	2338.9	33.196	122064	2.54%	140404	1.95%	41630	2.00%	2.16%	0.32%
Glycan#35	H3N6S1	1138.4108	2274.81	33.217	7123	0.15%	6138	0.09%	2903	0.14%	0.12%	0.03%
Glycan#36	H4N5F2S1	1263.9777	2525.94	33.322	14723	0.31%	19485	0.27%	13021	0.62%	0.40%	0.19%
Glycan#37	H6N3S1	1076.9116	2151.8	33.377	30362	0.63%	28418	0.40%	4929	0.24%	0.42%	0.20%
Glycan#38	H5N4F1S2	1316.0013	2629.9	33.469	60066	1.25%	45035	0.63%	11227	0.54%	0.80%	0.39%
Glycan#39	H5N4S2	1242.9738	2483.93	33.825	143660	2.98%	287848	4.01%	52066	2.50%	3.16%	0.77%
Glycan#40	H5N5F1S1	1271.9944	2541.97	33.918	129875	2.70%	215126	2.99%	22442	1.08%	2.26%	1.03%
Glycan#41	H5N4S2	1242.9733	2483.93	34.79	74846	1.55%	113426	1.58%	40428	1.94%	1.69%	0.22%
Glycan#42	H5N4F1S2	1316.0026	2629.99	34.972	27456	0.57%	27810	0.39%	8645	0.41%	0.46%	0.10%
Glycan#43	H5N4S2	1242.9711	2483.93	35.373	1523993	31.65%	2803393	39.02%	921713	44.20%	38.29%	6.31%
Glycan#44	H6N5S1	1280.0163	2558.02	35.791	34527	0.72%	44937	0.63%	46503	2.23%	1.19%	0.90%
Glycan#45	H6N5S1	1280.0076	2558	36.295	22272	0.46%	26464	0.37%	16668	0.80%	0.54%	0.23%
Glycan#46	H5N4F1S2	1316.0028	2629.99	36.629	175033	3.64%	226831	3.16%	76273	3.66%	3.48%	0.28%
Glycan#47	H5N5F1S2	1417.5411	2833.07	36.9	89212	1.85%	85850	1.19%	12786	0.61%	1.22%	0.62%
Glycan#48	H6N5S1	1279.9924	2557.97	37.241	14136	0.29%	29209	0.41%	8208	0.39%	0.36%	0.06%
Glycan#49	H9N2	1072.9035	2143.79	37.582	22451	0.47%	20289	0.28%	8449	0.41%	0.38%	0.09%
Glycan#50	H6N5S2	1425.538	2849.06	38.189	21810	0.45%	24284	0.34%	6676	0.32%	0.37%	0.07%
Glycan#51	H6N5S3	1571.084	3140.13	39.822	9229	0.19%	16799	0.23%	3034	0.15%	0.19%	0.04%
Glycan#52	H6N5F1S2	1498.568	2995.12	40.15	3347	0.07%	6077	0.08%	2471	0.12%	0.09%	0.03%
Glycan#53	H6N5S3	1571.0865	3140.15	41.134	112435	2.34%	107540	1.50%	24301	1.17%	1.67%	0.60%
Glycan#54	H6N5F1S2	1498.5671	2995.12	41.475	1749	0.04%	2205	0.03%	1545	0.07%	0.05%	0.02%
Glycan#55	H6N5S3	1571.0866	3140.16	42.49	75127	1.56%	71493	1.00%	24299	1.17%	1.24%	0.29%
Glycan#56	H6N5F1S3	1644.1149	3286.22	42.885	37513	0.78%	56522	0.79%	19036	0.91%	0.83%	0.08%
Glycan#57	H6N5F2S3	1145.1000([M+3H]3+)	3432.27	43.734	5688	0.12%	8353	0.12%	3911	0.19%	0.14%	0.04%
Glycan#58	H6N7F1S1	1556.0836	3110.15	45.02	14111	0.29%	8535	0.12%	2957	0.14%	0.18%	0.09%
Glycan#59	H6N7F1S1	1556.0834	3110.15	46.311	2376	0.05%	5203	0.07%	2743	0.13%	0.08%	0.04%
Glycan#60	H7N6F1S4	1315.1621([M+3H]3+)	3942.47	46.342	2418	0.05%	9624	0.13%	1968	0.09%	0.09%	0.04%
Glycan#61	H6N7F2S1	1629.1117	3256.21	46.785	6011	0.12%	8371	0.12%	1726	0.08%	0.11%	0.02%
Glycan#62	H7N6F1S4	1315.1621([M+3H]3+)	3942.47	47.173	4205	0.09%	10504	0.15%	2512	0.12%	0.12%	0.03%
Glycan#63	H7N6F2S4	1363.8442([M+3H]3+)	4088.51	47.44	1999	0.04%	6480	0.09%	1414	0.07%	0.07%	0.02%
Glycan#64	H7N6F1S4	1315.1621([M+3H]3+)	3942.47	48.024	2075	0.04%	6333	0.09%	1526	0.07%	0.07%	0.02%
Glycan#65	H7N6F2S4	1363.8442([M+3H]3+)	4088.51	48.299	1561	0.03%	5391	0.08%	1088	0.05%	0.05%	0.02%
Glycan#66	H7N6F3S4	1412.5293([M+3H]3+)	4234.57	49.02	1129	0.02%	3082	0.04%	1189	0.06%	0.04%	0.02%
	total ion count				4815060	100.00%	7184406	100.00%	2085470	100.00%	100.00%	

Table S4. Human lung cancer serum stage 3 samples N-glycan composition and totol ion counts based on LC-MS profile.

					HLCS3-1		HLCS3-2		HLCS3-3		HLCS3-4		HLCS3-5		STAGE 3 average	
Glycan#	composition	observed Mass [M+2H]2+	Calc. Mass	Retention	Abund	% abund	Abund	% abund	Abund	% abund	Abund	% abund	Abund	% abund	% abund	STDEV
Glycan#1	H3N5	891.3651	1780.7	20.689	35565	0.53%	42526	0.68%	37296	0.62%	55175	0.74%	33116	0.46%	0.61%	0.11%
Glycan#2	H3N4F1	862.8549	1723.7	20.885	697710	10.44%	653951	10.48%	547534	9.10%	1231086	16.61%	606494	8.37%	11.00%	3.26%
Glycan#3	H5N2	748.7996	1495.58	22.32	104415	1.56%	120354	1.93%	108611	1.81%	96704	1.30%	141998	1.96%	1.71%	0.28%
Glycan#4	H4N2	667.7729	1333.53	22.324	13860	0.21%	17734	0.28%	16831	0.28%	10709	0.14%	24950	0.34%	0.25%	0.08%
Glycan#5	H3N5F1	964.394	1926.77	22.355	146161	2.19%	146727	2.35%	125107	2.08%	166491	2.25%	135888	1.88%	2.15%	0.18%
Glycan#6	H4N4	870.852	1739.69	23.102	9889	0.15%	15934	0.26%	16512	0.27%	11631	0.16%	17181	0.24%	0.21%	0.06%
Glycan#7	H4N5	972.392	1942.77	24.293	18010	0.27%	21271	0.34%	17580	0.29%	20784	0.28%	14410	0.20%	0.28%	0.05%
Glycan#8	H5N6F1	1227.9447	2453.87	24.456	5684	0.09%	5143	0.08%	20342	0.34%	25327	0.34%	26741	0.37%	0.24%	0.15%
Glycan#9	H4N4F1	943.8816	1885.75	24.773	211309	3.16%	200628	3.22%	166646	2.77%	175780	2.37%	159224	2.20%	2.74%	0.46%
Glycan#10	H5N6F1	1227.9443	2453.87	25.083	10047	0.15%	9810	0.16%	24096	0.40%	38658	0.52%	29270	0.40%	0.33%	0.17%
Glycan#11	H5N6F1	1227.9445	2453.87	25.488	10369	0.16%	11748	0.19%	34238	0.57%	49683	0.67%	48517	0.67%	0.45%	0.26%
Glycan#12	H4N4F1	943.8813	1885.75	25.576	100167	1.50%	95173	1.53%	86067	1.43%	115946	1.56%	69570	0.96%	1.40%	0.25%
Glycan#13	H4N5F1	1045.4204	2088.83	25.701	103814	1.55%	111528	1.79%	90200	1.50%	79020	1.07%	95643	1.32%	1.45%	0.27%
Glycan#14	H6N2	829.8252	1657.64	26.28	21233	0.32%	25153	0.40%	9487	0.16%	14322	0.19%	19943	0.28%	0.27%	0.10%
Glycan#15	H4N3S1	914.8596	1827.7	26.374	50843	0.76%	43894	0.70%	64969	1.08%	84451	1.14%	83212	1.15%	0.97%	0.22%
Glycan#16	H4N5F1	1045.4197	2088.82	26.436	18197	0.27%	18558	0.30%	19451	0.32%	32080	0.43%	18310	0.25%	0.32%	0.07%
Glycan#17	H6N2	829.8253	1657.63	26.795	34346	0.51%	36978	0.59%	33914	0.56%	31393	0.42%	50033	0.69%	0.56%	0.10%
Glycan#18	H5N4	951.8779	1901.74	27.732	41716	0.62%	38531	0.62%	42914	0.71%	31872	0.43%	53432	0.74%	0.62%	0.12%
Glycan#19	H4N3F1S1	987.8889	1973.76	28.182	13639	0.20%	14497	0.23%	12131	0.20%	13567	0.18%	27920	0.39%	0.24%	0.08%
Glycan#20	H4N4S1	1016.3989	2030.78	28.392	41642	0.62%	44208	0.71%	70820	1.18%	97193	1.31%	80780	1.12%	0.99%	0.30%
Glycan#21	H4N5F1S1	1190.9667	2379.92	29.26	10105	0.15%	17028	0.27%	15538	0.26%	28519	0.38%	16420	0.23%	0.26%	0.08%
Glycan#22	H5N4F1	1024.9074	2047.8	29.29	92772	1.39%	104462	1.67%	88891	1.48%	59228	0.80%	92940	1.28%	1.32%	0.33%
Glycan#23	H5N5F1	1126.446	2250.88	29.626	36370	0.54%	45631	0.73%	32020	0.53%	31033	0.42%	36106	0.50%	0.55%	0.12%
Glycan#24	H5N3S1	995.8861	1989.76	29.694	40508	0.61%	53405	0.86%	42153	0.70%	59849	0.81%	48304	0.67%	0.73%	0.10%
Glycan#25	H4N5S1	1117.9387	2233.86	29.733	52240	0.78%	65385	1.05%	64656	1.07%	105166	1.42%	53634	0.74%	1.01%	0.27%
Glycan#26	H4N4F1S1	1089.4281	2176.84	29.94	35520	0.53%	34092	0.55%	35483	0.59%	45672	0.62%	28493	0.39%	0.54%	0.09%
Glycan#27	H7N2	910.8511	1819.69	31.013	6515	0.10%	7071	0.11%	5483	0.09%	6961	0.09%	6124	0.08%	0.10%	0.01%
Glycan#28	H4N5F1S1	1190.9684	2379.92	31.126	25503	0.38%	30190	0.48%	28640	0.48%	52928	0.71%	68828	0.95%	0.60%	0.23%
Glycan#29	H5N4S1	1097.4254	2192.84	31.35	88688	1.33%	64243	1.03%	57775	0.96%	72055	0.97%	77910	1.08%	1.07%	0.15%
Glycan#30	H5N4F1S1	1170.4535	2338.89	31.548	45167	0.68%	34942	0.56%	58604	0.97%	39978	0.54%	60566	0.84%	0.72%	0.19%
Glycan#31	H5N4S1	1097.4236	2192.83	31.81	665543	9.96%	632048	10.13%	484971	8.06%	640733	8.65%	687135	9.49%	9.26%	0.88%
Glycan#32	H5N6F3	1374.0383	2746.06	31.833	33045	0.49%	24363	0.39%	38672	0.64%	45004	0.61%	48955	0.68%	0.56%	0.12%
Glycan#33	H5N5S1	1198.965	2395.92	32.788	62632	0.94%	100476	1.61%	73566	1.22%	117260	1.58%	58154	0.80%	1.23%	0.37%
Glycan#34	H5N4F1S1	1170.4553	2338.9	33.196	137453	2.06%	173321	2.78%	137430	2.28%	158898	2.14%	183159	2.53%	2.36%	0.29%
Glycan#35	H3N6S1	1138.4108	2274.81	33.217	10527	0.16%	9075	0.15%	6332	0.11%	9701	0.13%	11609	0.16%	0.14%	0.02%
Glycan#36	H4N5F2S1	1263.9777	2525.94	33.322	19079	0.29%	13352	0.21%	29376	0.49%	36533	0.49%	41959	0.58%	0.41%	0.15%
Glycan#37	H6N3S1	1076.9116	2151.8	33.377	16211	0.24%	24822	0.40%	17794	0.30%	20025	0.27%	19991	0.28%	0.30%	0.06%
Glycan#38	H5N4F1S2	1316.0013	2629.9	33.469	54552	0.82%	48075	0.77%	70997	1.18%	45169	0.61%	78842	1.09%	0.89%	0.24%
Glycan#39	H5N4S2	1242.9738	2483.93	33.825	220648	3.30%	230644	3.70%	362068	6.02%	141264	1.91%	316220	4.37%	3.86%	1.51%
Glycan#40	H5N5F1S1	1271.9944	2541.97	33.918	130535	1.95%	180570	2.89%	103242	1.72%	131684	1.78%	155267	2.14%	2.10%	0.48%
Glycan#41	H5N4S2	1242.9733	2483.93	34.79	126844	1.90%	100511	1.61%	119066	1.98%	106109	1.43%	114537	1.58%	1.70%	0.23%
Glycan#42	H5N4F1S2	1316.0026	2629.99	34.972	28182	0.42%	31867	0.51%	37862	0.63%	25735	0.35%	33059	0.46%	0.47%	0.11%
Glycan#43	H5N4S2	1242.9711	2483.93	35.373	2020979	30.25%	1699732	27.25%	1888978	31.41%	2199838	29.69%	2345235	32.38%	30.19%	1.95%
Glycan#44	H6N5S1	1280.0163	2558.02	35.791	36916	0.55%	21936	0.35%	44838	0.75%	58138	0.78%	59753	0.83%	0.65%	0.20%
Glycan#45	H6N5S1	1280.0076	2558	36.295	27121	0.41%	20383	0.33%	21693	0.36%	28875	0.39%	30589	0.42%	0.38%	0.04%
Glycan#46	H5N4F1S2	1316.0028	2629.99	36.629	343690	5.14%	292118	4.68%	222403	3.70%	230898	3.12%	231098	3.19%	3.97%	0.91%
Glycan#47	H5N5F1S2	1417.5411	2833.07	36.9	73715	1.10%	135666	2.17%	83290	1.38%	132555	1.79%	63941	0.88%	1.47%	0.52%
Glycan#48	H6N5S1	1279.9924	2557.97	37.241	31559	0.47%	17336	0.28%	14957	0.25%	20337	0.27%	23774	0.33%	0.32%	0.09%
Glycan#49	H9N2	1072.9035	2143.79	37.582	19968	0.30%	18181	0.29%	16398	0.27%	18021	0.24%	28448	0.39%	0.30%	0.06%
Glycan#50	H6N5S2	1425.538	2849.06	38.189	33415	0.50%	37991	0.61%	22985	0.38%	22621	0.31%	35897	0.50%	0.46%	0.12%
Glycan#51	H6N5S3	1571.084	3140.13	39.822	10031	0.15%	15024	0.24%	15660	0.26%	6374	0.09%	10495	0.14%	0.18%	0.07%
Glycan#52	H6N5F1S2	1498.568	2995.12	40.15	23512	0.35%	3810	0.06%	5033	0.08%	11523	0.16%	14416	0.20%	0.17%	0.12%
Glycan#53	H6N5S3	1571.0865	3140.15	41.134	87196	1.30%	141444	2.27%	115758	1.92%	83007	1.12%	128797	1.78%	1.68%	0.47%
Glycan#54	H6N5F1S2	1498.5671	2995.12	41.475	8245	0.12%	1970	0.03%	1595	0.03%	6841	0.09%	5777	0.08%	0.07%	0.04%
Glycan#55	H6N5S3	1571.0866	3140.16	42.49	104780	1.57%	63637	1.02%	46315	0.77%	93565	1.26%	112542	1.55%	1.23%	0.34%
Glycan#56	H6N5F1S3	1644.1149	3286.22	42.885	110676	1.66%	23409	0.38%	32023	0.53%	75804	1.02%	99793	1.38%	0.99%	0.54%
Glycan#57	H6N5F2S3	1145.1000([M+3H]3+)	3432.27	43.734	23802	0.36%	4120	0.07%	6644	0.11%	10295	0.14%	14136	0.20%	0.17%	0.11%
Glycan#58	H6N7F1S1	1556.0836	3110.15	45.02	8631	0.13%	20728	0.33%	2170	0.04%	3024	0.04%	4471	0.06%	0.12%	0.12%
Glycan#59	H6N7F1S1	1556.0834	3110.15	46.311	6410	0.10%	7004	0.11%	1213	0.02%	3498	0.05%	2534	0.03%	0.06%	0.04%
Glycan#60	H7N6F1S4	1315.1621([M+3H]3+)	3942.47	46.342	11197	0.17%	3498	0.06%	7055	0.12%	6383	0.09%	11443	0.16%	0.12%	0.05%
Glycan#61	H6N7F2S1	1629.1117	3256.21	46.785	17545	0.26%	4572	0.07%	787	0.01%	2173	0.03%	2131	0.03%	0.08%	0.10%
Glycan#62	H7N6F1S4	1315.1621([M+3H]3+)	3942.47	47.173	17930	0.27%	2609	0.04%	4590	0.08%	17321	0.23%	13952	0.19%	0.16%	0.10%
Glycan#63	H7N6F2S4	1363.8442([M+3H]3+)	4088.51	47.44	11378	0.17%	1373	0.02%	1600	0.03%	4323	0.06%	9978	0.14%	0.08%	0.07%
Glycan#64	H7N6F1S4	1315.1621([M+3H]3+)	3942.47	48.024	7933	0.12%	908	0.01%	1680	0.03%	6549	0.09%	7722	0.11%	0.07%	0.05%
Glycan#65	H7N6F2S4	1363.8442([M+3H]3+)	4088.51	48.299	12404	0.19%	650	0.01%	994	0.02%	4394	0.06%	7163	0.10%	0.07%	0.07%
Glycan#66	H7N6F3S4	1412.5293([M+3H]3+)	4234.57	49.02	5553	0.08%	613	0.01%	823	0.01%	2841	0.04%	3255	0.04%	0.04%	0.03%
	total ion count				6681841	100.00%	6238631	100.00%	6014847	100.00%	7410574	100.00%	7242184	100.00%	100.00%	

Table S5. Human lung cancer serum stage 4 samples N-glycan composition and totol ion counts based on LC-MS profile.

					HLCS4-1		HLCS4-2		HLCS4-3		HLCS4-4		HLCS4-5		HLCS4-6		STAGE 4 average	
Glycan#	composition	observed Mass [M+2H]2+	Calc. Mass	Retention	Abund	% abund	Abund	% abund	Abund	% abund	Abund	% abund	Abund	% abund	Abund	% abund	% abund	STDEV
Glycan#1	H3N5	891.3651	1780.7	20.689	59270	0.85%	70330	0.91%	13005	0.49%	14140	0.54%	150388	1.19%	28463	0.37%	0.73%	0.31%
Glycan#2	H3N4F1	862.8549	1723.7	20.885	414885	5.95%	979846	12.71%	116188	4.41%	161247	6.18%	1859040	14.66%	445619	5.77%	8.28%	4.28%
Glycan#3	H5N2	748.7996	1495.58	22.32	127796	1.83%	108303	1.40%	42979	1.63%	45013	1.72%	204309	1.61%	142453	1.85%	1.68%	0.16%
Glycan#4	H4N2	667.7729	1333.53	22.324	18918	0.27%	14870	0.19%	7202	0.27%	5636	0.22%	35888	0.28%	22322	0.29%	0.25%	0.04%
Glycan#5	H3N5F1	964.394	1926.77	22.355	88196	1.27%	164462	2.13%	38589	1.46%	49585	1.90%	409284	3.23%	96550	1.25%	1.87%	0.75%
Glycan#6	H4N4	870.852	1739.69	23.102	19761	0.28%	7544	0.10%	6558	0.25%	13411	0.51%	19081	0.15%	12365	0.16%	0.24%	0.15%
Glycan#7	H4N5	972.392	1942.77	24.293	34592	0.50%	30134	0.39%	7170	0.27%	7897	0.30%	66807	0.53%	16364	0.21%	0.37%	0.13%
Glycan#8	H5N6F1	1227.9447	2453.87	24.456	4318	0.06%	4243	0.06%	13658	0.52%	9212	0.35%	15892	0.13%	30423	0.39%	0.25%	0.20%
Glycan#9	H4N4F1	943.8816	1885.75	24.773	188879	2.71%	376289	4.88%	45097	1.71%	57255	2.19%	656532	5.18%	171663	2.22%	3.15%	1.49%
Glycan#10	H5N6F1	1227.9443	2453.87	25.083	11126	0.16%	12256	0.16%	21967	0.83%	20750	0.79%	23571	0.19%	34024	0.44%	0.43%	0.32%
Glycan#11	H5N6F1	1227.9445	2453.87	25.488	11579	0.17%	13764	0.18%	33195	1.26%	27757	1.06%	27881	0.22%	42360	0.55%	0.57%	0.48%
Glycan#12	H4N4F1	943.8813	1885.75	25.576	101771	1.46%	194680	2.52%	34462	1.31%	38442	1.47%	302522	2.39%	117421	1.52%	1.78%	0.53%
Glycan#13	H4N5F1	1045.4204	2088.83	25.701	92929	1.33%	138649	1.80%	26963	1.02%	31923	1.22%	193053	1.52%	84654	1.10%	1.33%	0.29%
Glycan#14	H6N2	829.8252	1657.64	26.28	20455	0.29%	27929	0.36%	5565	0.21%	5394	0.21%	26196	0.21%	14037	0.18%	0.24%	0.07%
Glycan#15	H4N3S1	914.8596	1827.7	26.374	63679	0.91%	50204	0.65%	32320	1.23%	32219	1.23%	109576	0.86%	79823	1.03%	0.99%	0.23%
Glycan#16	H4N5F1	1045.4197	2088.82	26.436	18389	0.26%	33836	0.44%	8159	0.31%	6980	0.27%	55644	0.44%	19634	0.25%	0.33%	0.09%
Glycan#17	H6N2	829.8253	1657.63	26.795	35140	0.50%	49914	0.65%	16899	0.64%	14537	0.56%	59587	0.47%	55272	0.72%	0.59%	0.09%
Glycan#18	H5N4	951.8779	1901.74	27.732	63562	0.91%	33897	0.44%	17972	0.68%	18878	0.72%	82959	0.65%	64177	0.83%	0.71%	0.16%
Glycan#19	H4N3F1S1	987.8889	1973.76	28.182	13549	0.19%	35524	0.46%	7763	0.29%	9642	0.37%	21603	0.17%	13835	0.18%	0.28%	0.12%
Glycan#20	H4N4S1	1016.3989	2030.78	28.392	72693	1.04%	52179	0.68%	36260	1.38%	35282	1.35%	148054	1.17%	72827	0.94%	1.09%	0.26%
Glycan#21	H4N5F1S1	1190.9667	2379.92	29.26	18941	0.27%	25774	0.33%	4953	0.19%	5254	0.20%	45739	0.36%	7978	0.10%	0.24%	0.10%
Glycan#22	H5N4F1	1024.9074	2047.8	29.29	120543	1.73%	163593	2.12%	37756	1.43%	41821	1.60%	221803	1.75%	122631	1.59%	1.70%	0.23%
Glycan#23	H5N5F1	1126.446	2250.88	29.626	45711	0.66%	67210	0.87%	13823	0.52%	17119	0.66%	84248	0.66%	39579	0.51%	0.65%	0.13%
Glycan#24	H5N3S1	995.8861	1989.76	29.694	65607	0.94%	46727	0.61%	18475	0.70%	13674	0.52%	109670	0.86%	65050	0.84%	0.75%	0.16%
Glycan#25	H4N5S1	1117.9387	2233.86	29.733	149359	2.14%	102008	1.32%	20183	0.77%	21459	0.82%	365825	2.89%	56678	0.73%	1.45%	0.89%
Glycan#26	H4N4F1S1	1089.4281	2176.84	29.94	46759	0.67%	67803	0.88%	15427	0.59%	17113	0.66%	79581	0.63%	33557	0.43%	0.64%	0.14%
Glycan#27	H7N2	910.8511	1819.69	31.013	5425	0.08%	6506	0.08%	3285	0.12%	4962	0.19%	12249	0.10%	6894	0.09%	0.11%	0.04%
Glycan#28	H4N5F1S1	1190.9684	2379.92	31.126	25310	0.36%	49342	0.64%	8533	0.32%	16791	0.64%	89453	0.71%	20014	0.26%	0.49%	0.19%
Glycan#29	H5N4S1	1097.4254	2192.84	31.35	77662	1.11%	83453	1.08%	30898	1.17%	23252	0.89%	103728	0.82%	94509	1.22%	1.05%	0.16%
Glycan#30	H5N4F1S1	1170.4535	2338.89	31.548	52643	0.76%	33900	0.44%	31252	1.19%	25649	0.98%	96519	0.76%	71106	0.92%	0.84%	0.25%
Glycan#31	H5N4S1	1097.4236	2192.83	31.81	856122	12.28%	616329	7.99%	136523	5.18%	180273	6.91%	1149170	9.06%	848882	11.00%	8.74%	2.62%
Glycan#32	H5N6F3	1374.0383	2746.06	31.833	25915	0.37%	29917	0.39%	18985	0.72%	16134	0.62%	36113	0.28%	54246	0.70%	0.51%	0.19%
Glycan#33	H5N5S1	1198.965	2395.92	32.788	198368	2.85%	110316	1.43%	22486	0.85%	26577	1.02%	377676	2.98%	83244	1.08%	1.70%	0.96%
Glycan#34	H5N4F1S1	1170.4553	2338.9	33.196	220195	3.16%	312687	4.05%	81629	3.10%	82893	3.18%	372960	2.94%	190804	2.47%	3.15%	0.51%
Glycan#35	H3N6S1	1138.4108	2274.81	33.217	15618	0.22%	8964	0.12%	3330	0.13%	5944	0.23%	14038	0.11%	13521	0.18%	0.16%	0.05%
Glycan#36	H4N5F2S1	1263.9777	2525.94	33.322	16121	0.23%	14715	0.19%	13402	0.51%	11756	0.45%	25767	0.20%	41818	0.54%	0.35%	0.16%
Glycan#37	H6N3S1	1076.9116	2151.8	33.377	36230	0.52%	17910	0.23%	8611	0.33%	6537	0.25%	46182	0.36%	24872	0.32%	0.34%	0.10%
Glycan#38	H5N4F1S2	1316.0013	2629.9	33.469	67709	0.97%	38980	0.51%	38954	1.48%	30663	1.17%	107289	0.85%	76146	0.99%	0.99%	0.33%
Glycan#39	H5N4S2	1242.9738	2483.93	33.825	259331	3.72%	148790	1.93%	63217	2.40%	65885	2.52%	164507	1.30%	350930	4.55%	2.74%	1.19%
Glycan#40	H5N5F1S1	1271.9944	2541.97	33.918	166525	2.39%	230670	2.99%	50739	1.93%	69528	2.66%	371756	2.93%	141490	1.83%	2.46%	0.50%
Glycan#41	H5N4S2	1242.9733	2483.93	34.79	92086	1.32%	110091	1.43%	50282	1.91%	76982	2.95%	152223	1.20%	147967	1.92%	1.79%	0.64%
Glycan#42	H5N4F1S2	1316.0026	2629.99	34.972	36451	0.52%	23801	0.31%	20581	0.78%	13645	0.52%	53417	0.42%	41821	0.54%	0.52%	0.16%
Glycan#43	H5N4S2	1242.9711	2483.93	35.373	1824285	26.17%	1905358	24.71%	1042117	39.55%	931170	35.67%	2500284	19.72%	2484863	32.20%	29.67%	7.43%
Glycan#44	H6N5S1	1280.0163	2558.02	35.791	32772	0.47%	36752	0.48%	38509	1.46%	21090	0.81%	32561	0.26%	65404	0.85%	0.72%	0.43%
Glycan#45	H6N5S1	1280.0076	2558	36.295	30138	0.43%	20196	0.26%	12897	0.49%	6187	0.24%	21520	0.17%	41951	0.54%	0.36%	0.15%
Glycan#46	H5N4F1S2	1316.0028	2629.99	36.629	376350	5.40%	396888	5.15%	129735	4.92%	125854	4.82%	795955	6.28%	393904	5.10%	5.28%	0.53%
Glycan#47	H5N5F1S2	1417.5411	2833.07	36.9	184182	2.64%	184048	2.39%	27405	1.04%	28146	1.08%	347307	2.74%	82011	1.06%	1.82%	0.85%
Glycan#48	H6N5S1	1279.9924	2557.97	37.241	25307	0.36%	19325	0.25%	5041	0.19%	5917	0.23%	13076	0.10%	30693	0.40%	0.26%	0.11%
Glycan#49	H9N2	1072.9035	2143.79	37.582	18404	0.26%	15911	0.21%	9313	0.35%	8028	0.31%	31572	0.25%	31722	0.41%	0.30%	0.07%
Glycan#50	H6N5S2	1425.538	2849.06	38.189	45375	0.65%	14219	0.18%	6154	0.23%	6589	0.25%	19956	0.16%	51486	0.67%	0.36%	0.24%
Glycan#51	H6N5S3	1571.084	3140.13	39.822	18556	0.27%	4075	0.05%	3328	0.13%	2201	0.08%	5297	0.04%	17841	0.23%	0.13%	0.09%
Glycan#52	H6N5F1S2	1498.568	2995.12	40.15	7674	0.11%	17391	0.23%	2417	0.09%	6061	0.23%	11814	0.09%	13758	0.18%	0.16%	0.07%
Glycan#53	H6N5S3	1571.0865	3140.15	41.134	142401	2.04%	51213	0.66%	43081	1.63%	15124	0.58%	72971	0.58%	157457	2.04%	1.26%	0.73%
Glycan#54	H6N5F1S2	1498.5671	2995.12	41.475	2968	0.04%	12638	0.16%	2300	0.09%	2093	0.08%	7765	0.06%	4669	0.06%	0.08%	0.04%
Glycan#55	H6N5S3	1571.0866	3140.16	42.49	77415	1.11%	96817	1.26%	36019	1.37%	16702	0.64%	96056	0.76%	109534	1.42%	1.09%	0.32%
Glycan#56	H6N5F1S3	1644.1149	3286.22	42.885	49851	0.72%	97243	1.26%	26357	1.00%	27892	1.07%	93997	0.74%	77753	1.01%	0.97%	0.21%
Glycan#57	H6N5F2S3	1145.1000([M+3H]3+)	3432.27	43.734	9588	0.14%	20260	0.26%	6494	0.25%	7447	0.29%	35975	0.28%	18037	0.23%	0.24%	0.05%
Glycan#58	H6N7F1S1	1556.0836	3110.15	45.02	16677	0.24%	5057	0.07%	2285	0.09%	1088	0.04%	1151	0.01%	3729	0.05%	0.08%	0.08%
Glycan#59	H6N7F1S1	1556.0834	3110.15	46.311	5744	0.08%	10868	0.14%	1974	0.07%	1055	0.04%	2046	0.02%	3055	0.04%	0.07%	0.04%
Glycan#60	H7N6F1S4	1315.1621([M+3H]3+)	3942.47	46.342	8758	0.13%	8576	0.11%	2754	0.10%	2112	0.08%	5529	0.04%	7231	0.09%	0.09%	0.03%
Glycan#61	H6N7F2S1	1629.1117	3256.21	46.785	7616	0.11%	20918	0.27%	1241	0.05%	2306	0.09%	1473	0.01%	2337	0.03%	0.09%	0.09%
Glycan#62	H7N6F1S4	1315.1621([M+3H]3+)	3942.47	47.173	10720	0.15%	15021	0.19%	3167	0.12%	3856	0.15%	8801	0.07%	8717	0.11%	0.13%	0.04%
Glycan#63	H7N6F2S4	1363.8442([M+3H]3+)	4088.51	47.44	5011	0.07%	12097	0.16%	2136	0.08%	3725	0.14%	6824	0.05%	3789	0.05%	0.09%	0.05%
Glycan#64	H7N6F1S4	1315.1621([M+3H]3+)	3942.47	48.024	3234	0.05%	8786	0.11%	1578	0.06%	1926	0.07%	5895	0.05%	4534	0.06%	0.07%	0.03%
Glycan#65	H7N6F2S4	1363.8442([M+3H]3+)	4088.51	48.299	3869	0.06%	17027	0.22%	2335	0.09%	2272	0.09%	8778	0.07%	3428	0.04%	0.09%	0.06%
Glycan#66	H7N6F3S4	1412.5293([M+3H]3+)	4234.57	49.02	2618	0.04%	12418	0.16%	1139	0.04%	2415	0.09%	4612	0.04%	1944	0.03%	0.07%	0.05%
	total ion count				6969631	100.00%	7711441	100.00%	2635071	100.00%	2610367	100.00%	12678995	100.00%	7717870	100.00%	100.00%	

Table S6. The comparison of ORNG method with current glycan release methods for N-glycans.

N-glycan release				
**Method**	**ORNG**	**N-Glycanase**	**Hydrozinolysis**	**Ammounium Salts**
**Structural integrity**	High	High	Medium (De-N/O-acetylation, desialylation)	Medium (De-O-acetylation)
**Structural limitation**	Not known, N/O-Ac and O-sulfates are stable	PNGase F does not work with Core α-3 fucose	N/O-Acetyl	O-Acetyl
**MS analysis**	Easy	Easy	Medium	Medium
**Operation**	Easy and fast	Easy but lengthier with trypsin digestion pretreatment	Medium; high temperature and re-N-acetylation required	Lengthy, volatile salts in sealed container with heating
**Economy**	Extremely cheap	Expensive	Medium	Medium to cheap
**Reagents**	Mild and safe	Mild	Highly toxic	Potentially caustic and explosive
**Scale**	Medium to high (kilogram tissues possible)	Low to medium	Low to medium	Low to medium

IV. Mass Spectra

Figure S15. MS profile (1 of 2) generated by N-glycan oxidative release and specific labeling protocol of human IgG.

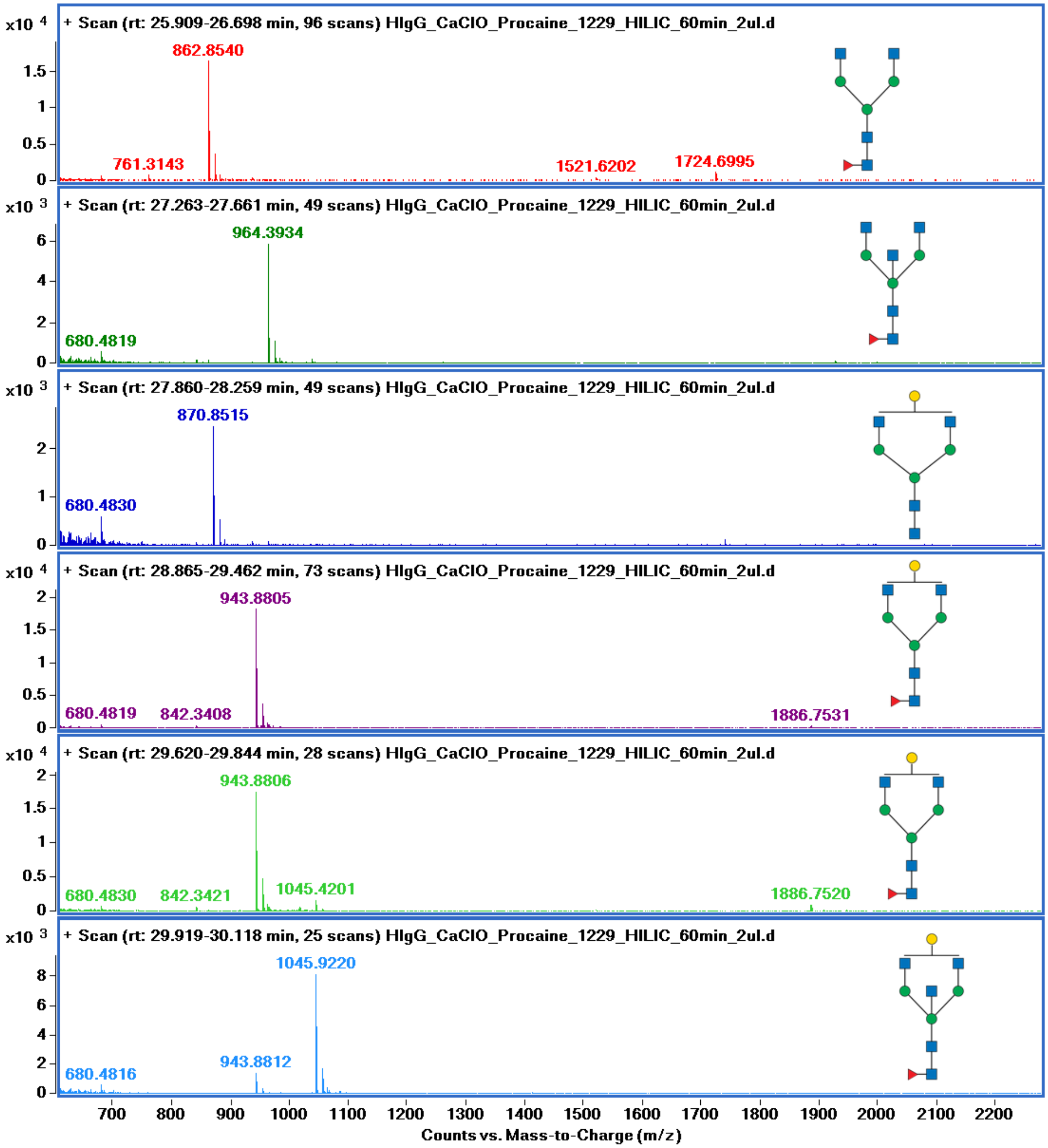

Figure S16. MS profile (2 of 2) generated by N-glycan oxidative release and specific labeling protocol of human IgG

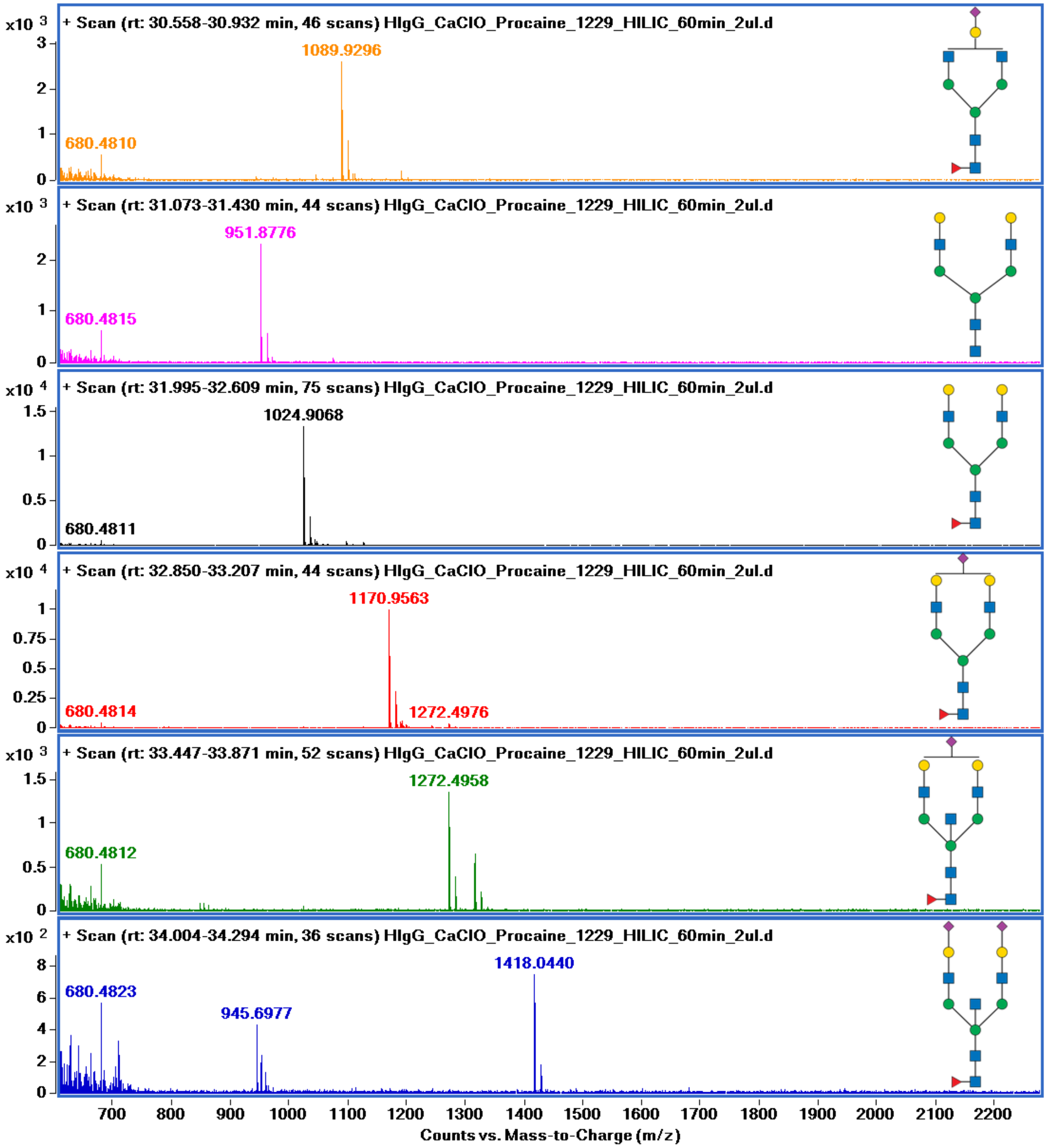

Figure S17. MS profile (1 of 2) generated by N-glycan oxidative release and specific labeling protocol of Bovine IgG.

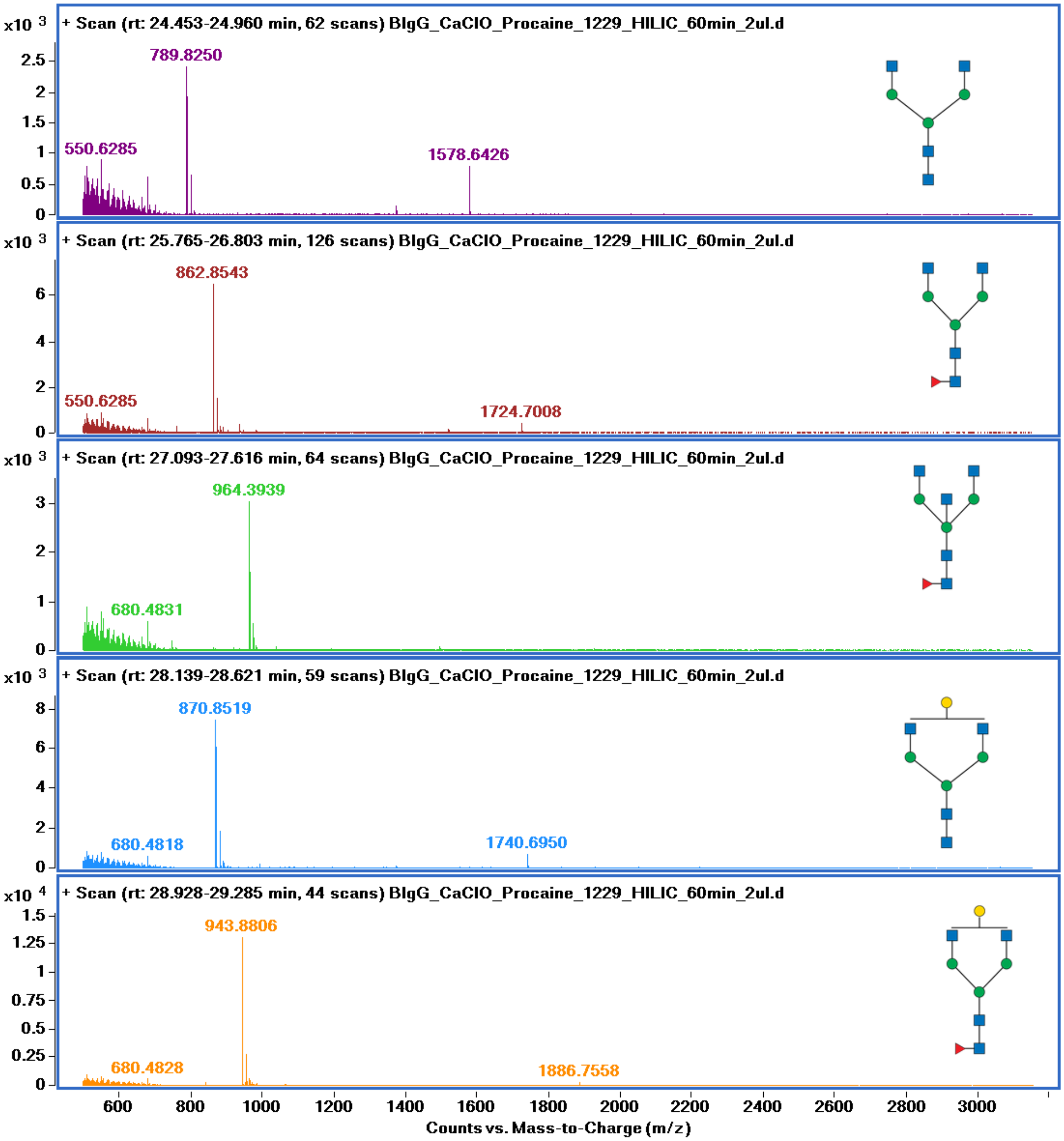

Figure S18. MS profile (2 of 2) generated by N-glycan oxidative release and specific labeling protocol of Bovine IgG

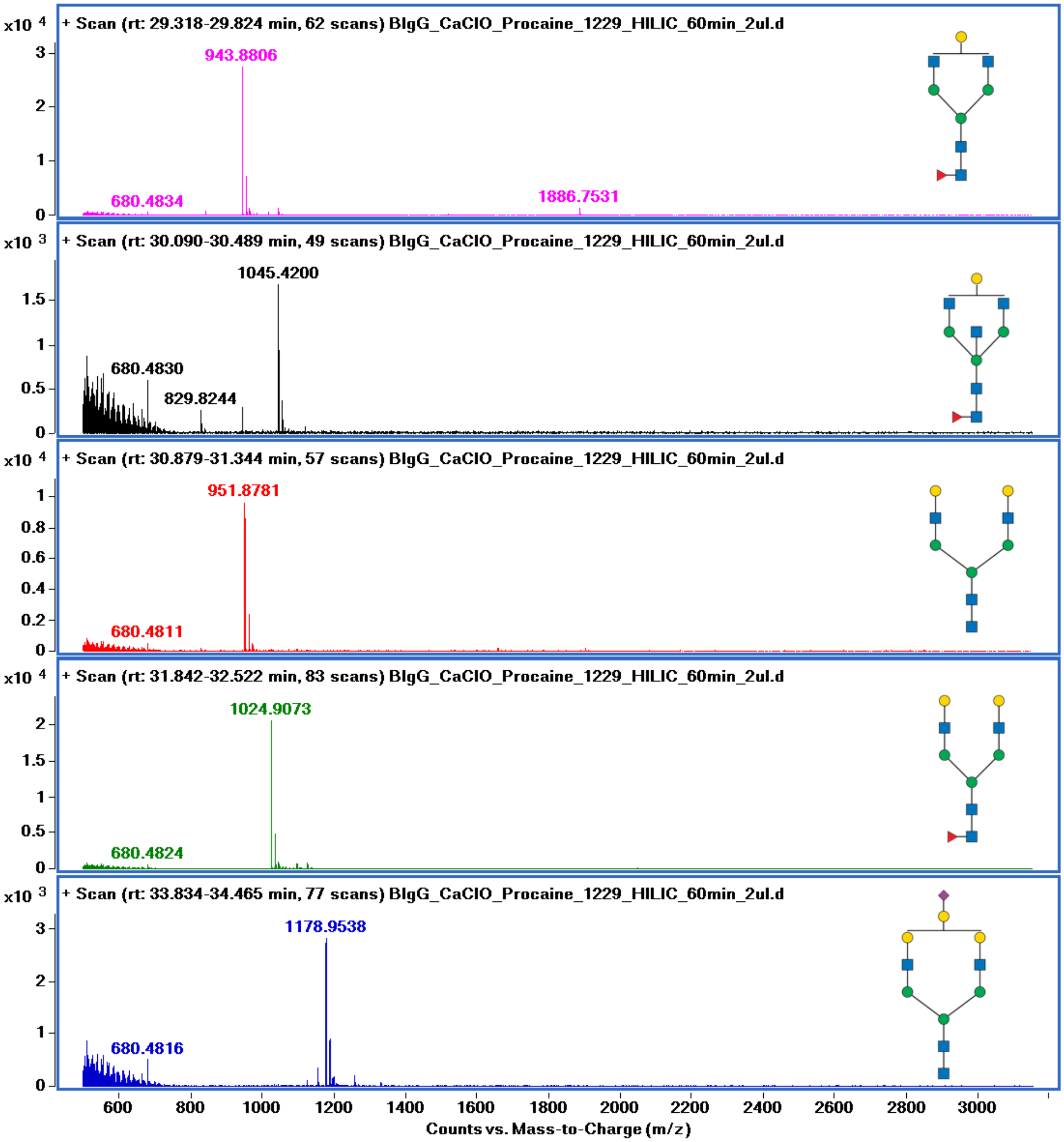

Figure S19. MS profile (1 of 2) generated by N-glycan oxidative release and specific labeling protocol of Fetuin

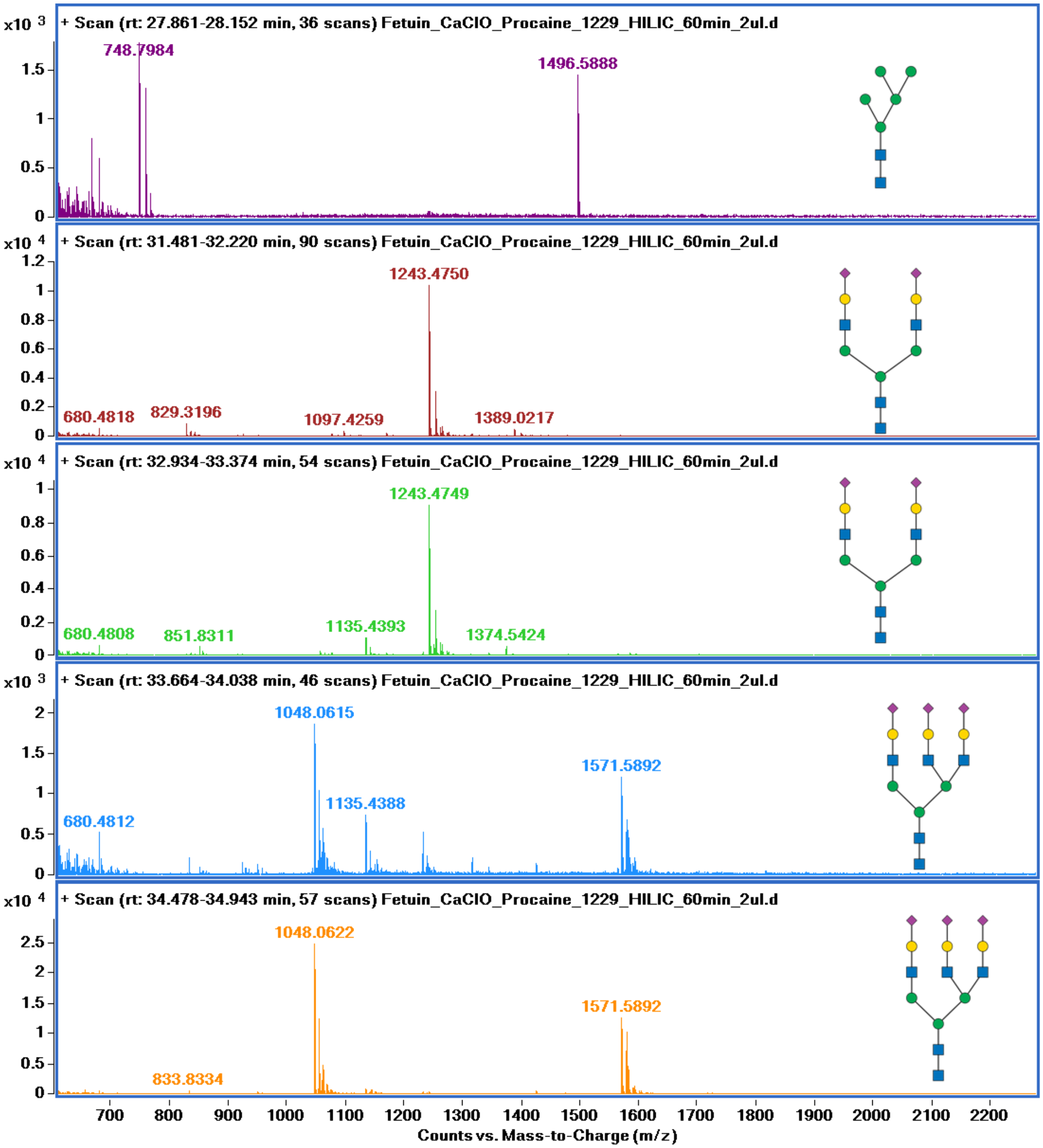

Figure S20. MS profile (2 of 2) generated by N-glycan oxidative release and specific labeling protocol of Fetuin

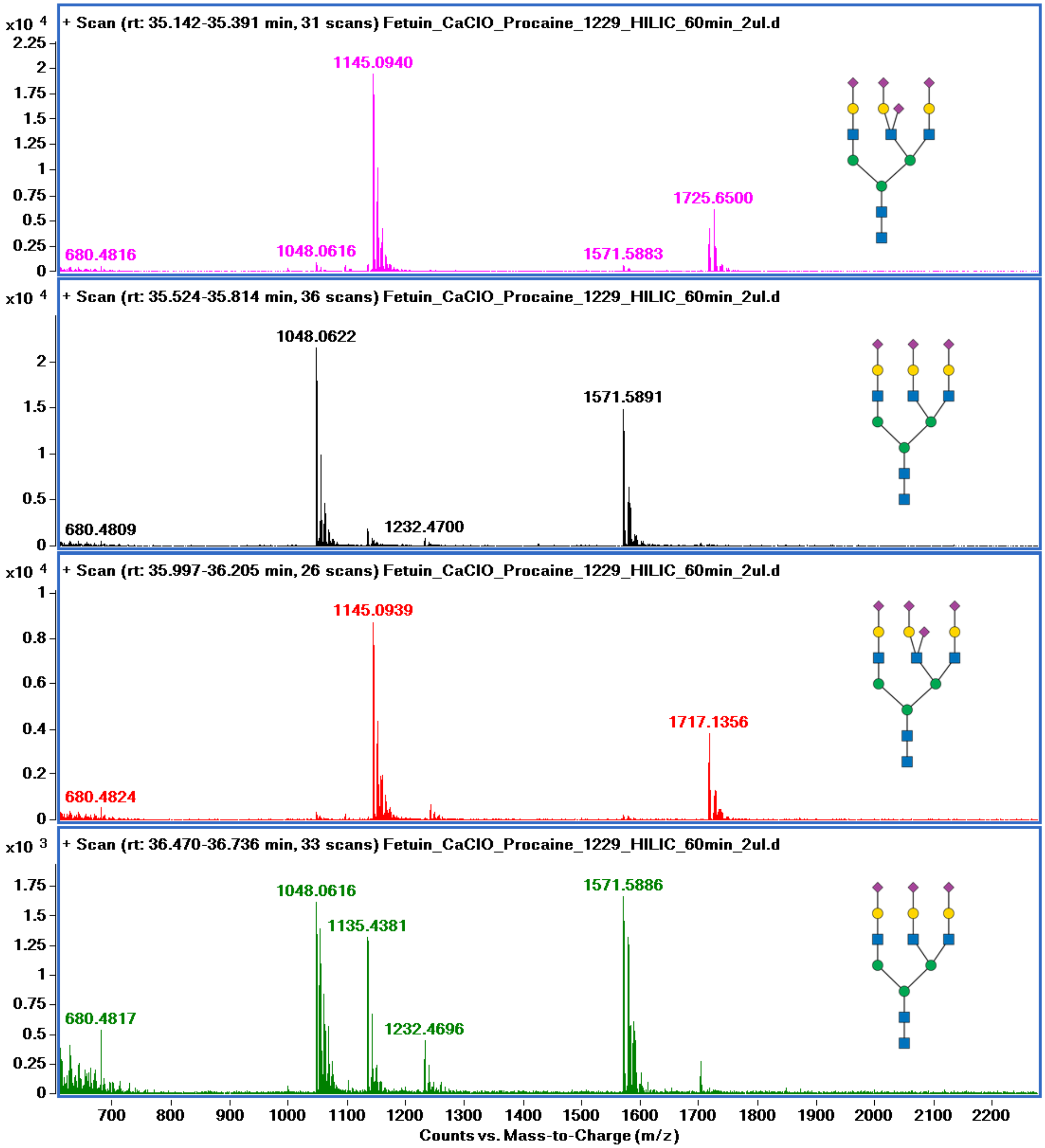

Figure S21. MS profile generated by N-glycan oxidative release and specific labeling protocol of RNase B

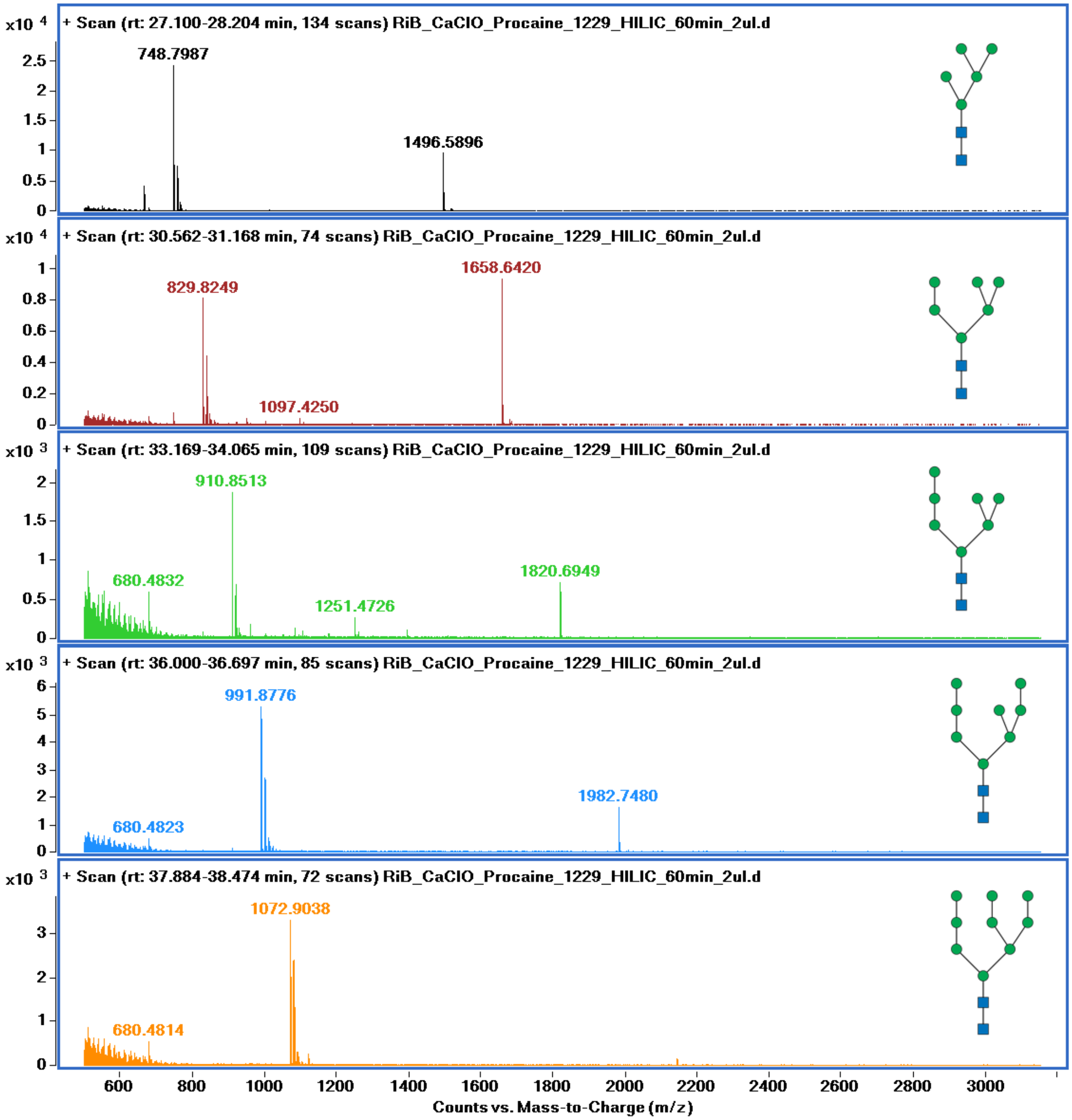

Figure S22. MS profile (1 of 2) generated by N-glycan oxidative release and specific labeling protocol of porcine plasma

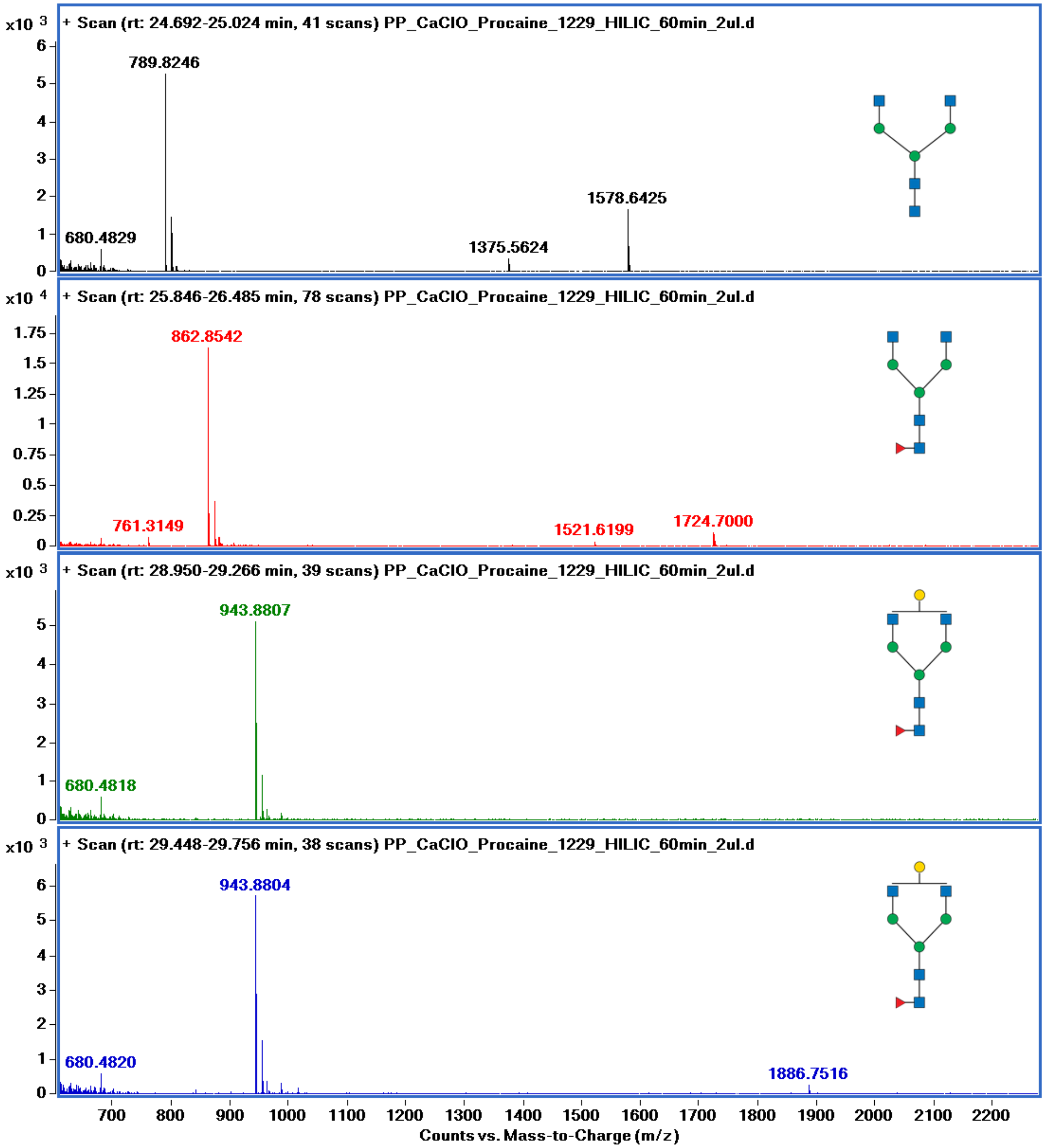

Figure S23. MS profile (2 of 2) generated by N-glycan oxidative release and specific labeling protocol of porcine plasma

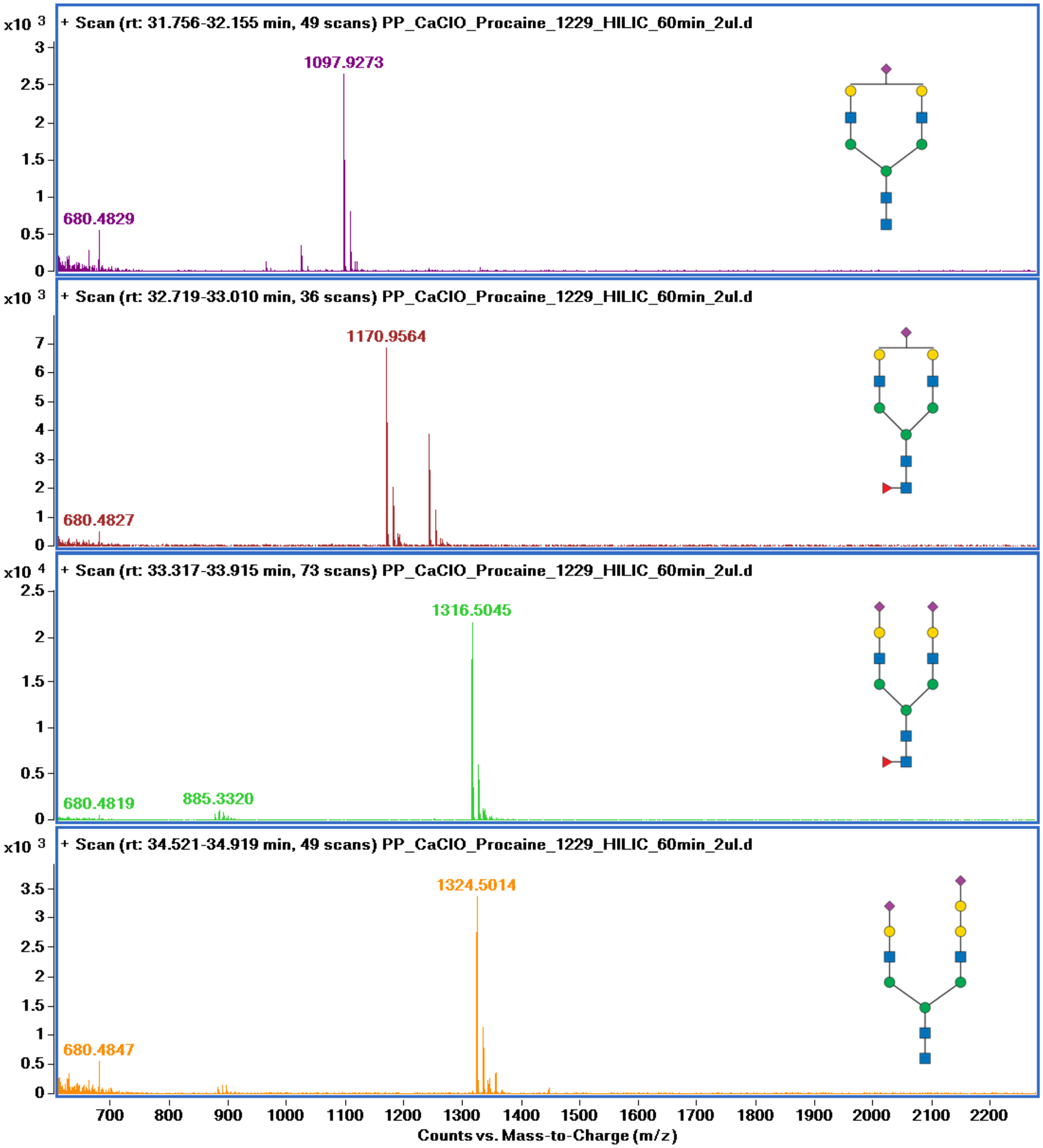

Figure S24. MS profile (1 of 2) generated by N-glycan oxidative release and specific labeling protocol of delipidated egg yolk

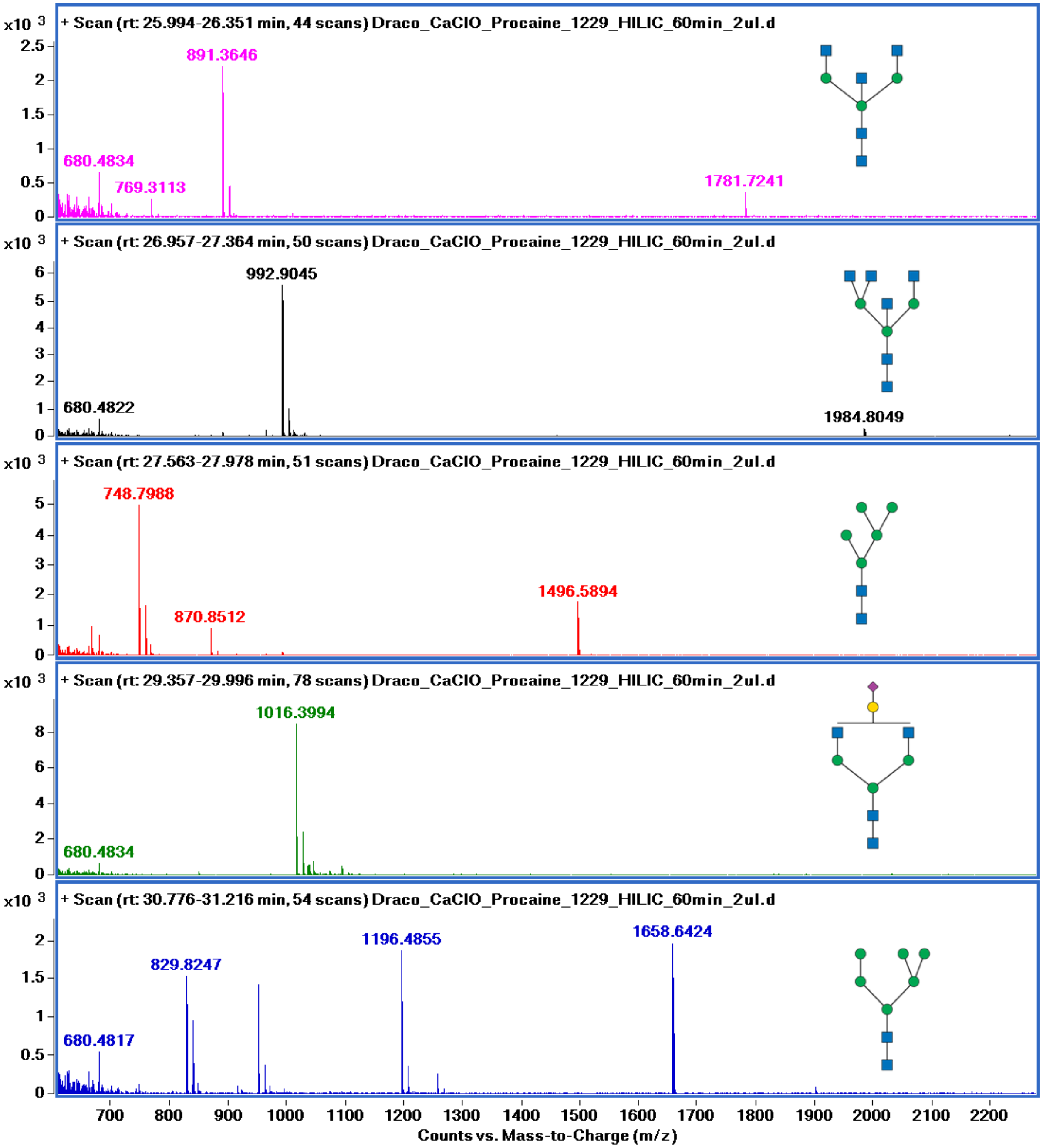

Figure S25. MS profile (2 of 2) generated by N-glycan oxidative release and specific labeling protocol of delipidated egg yolk

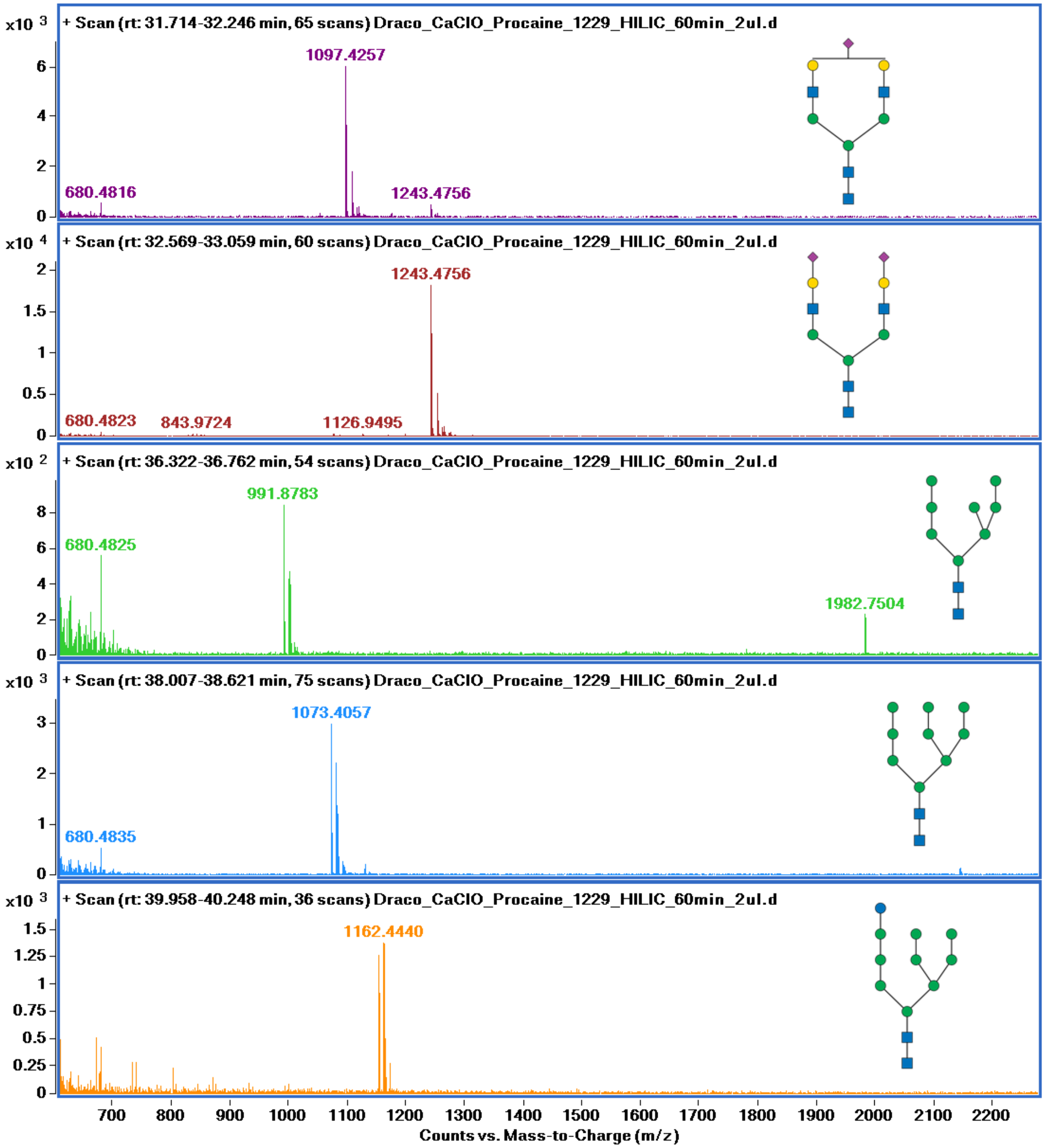

Figure S26. MS profile generated by N-glycan oxidative release and specific labeling protocol of soy protein

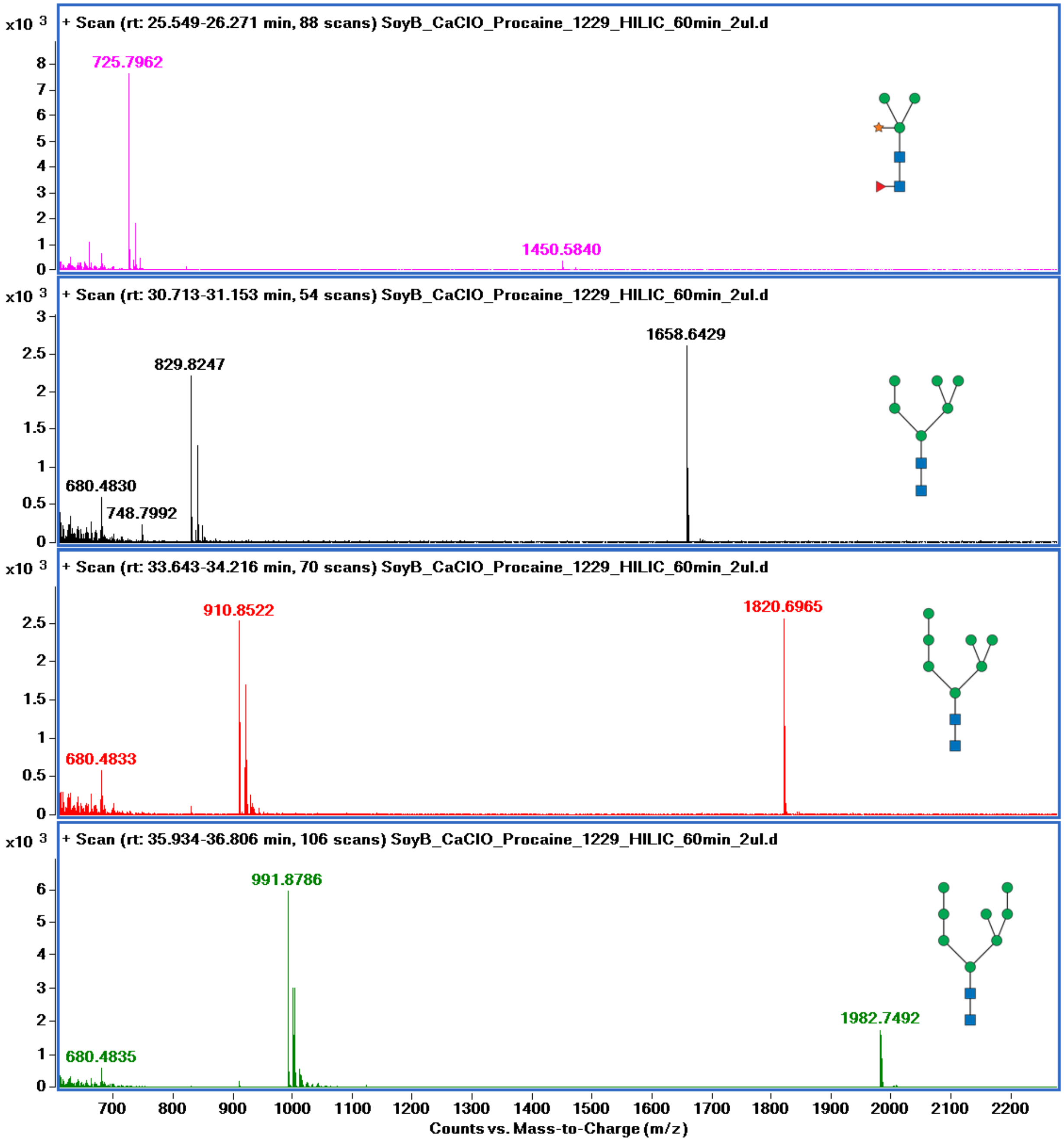

Figure S27. MS profile generated by N-glycan oxidative release and specific labeling protocol of soybean

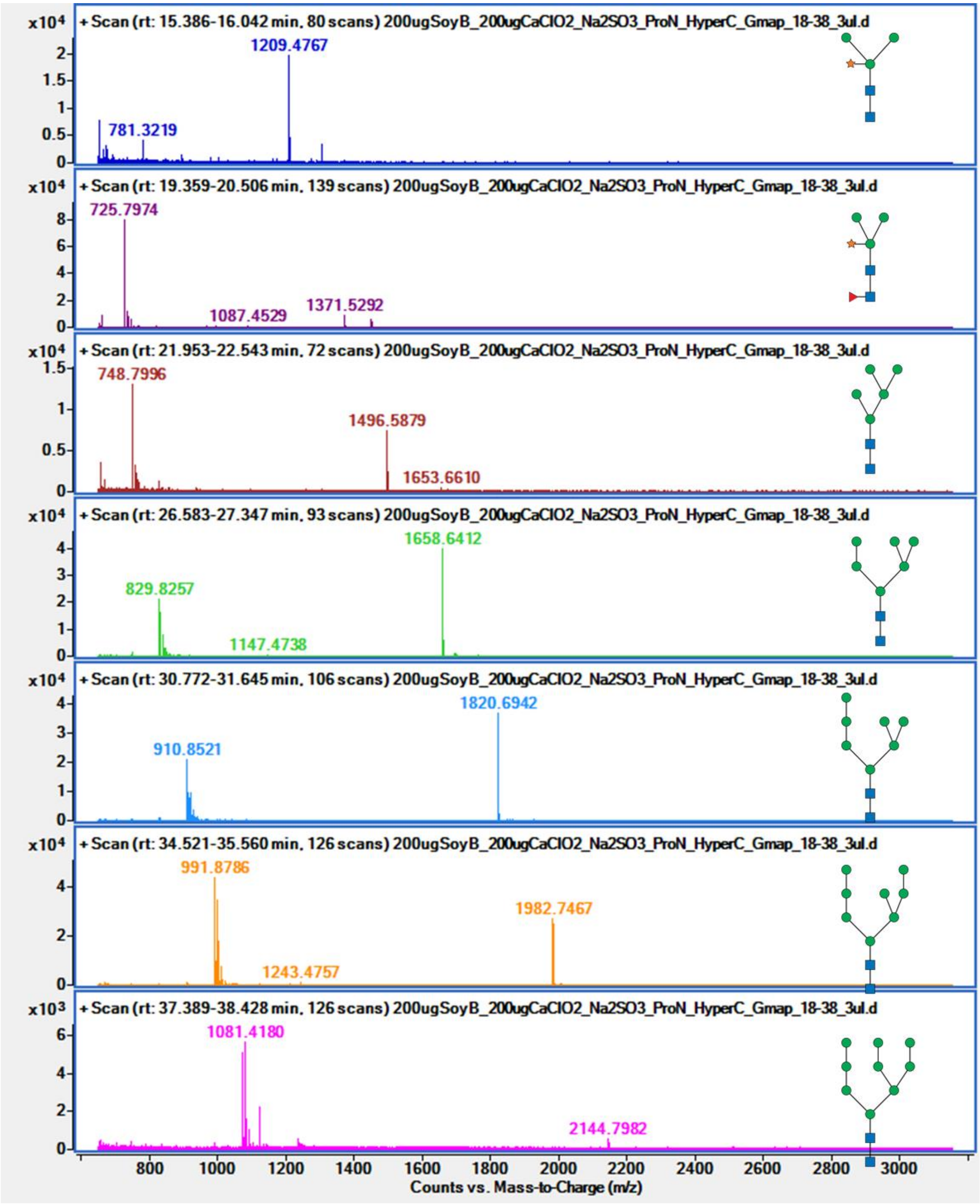

Figure S28. MS profile generated by N-glycan oxidative release and specific labeling protocol of black bean

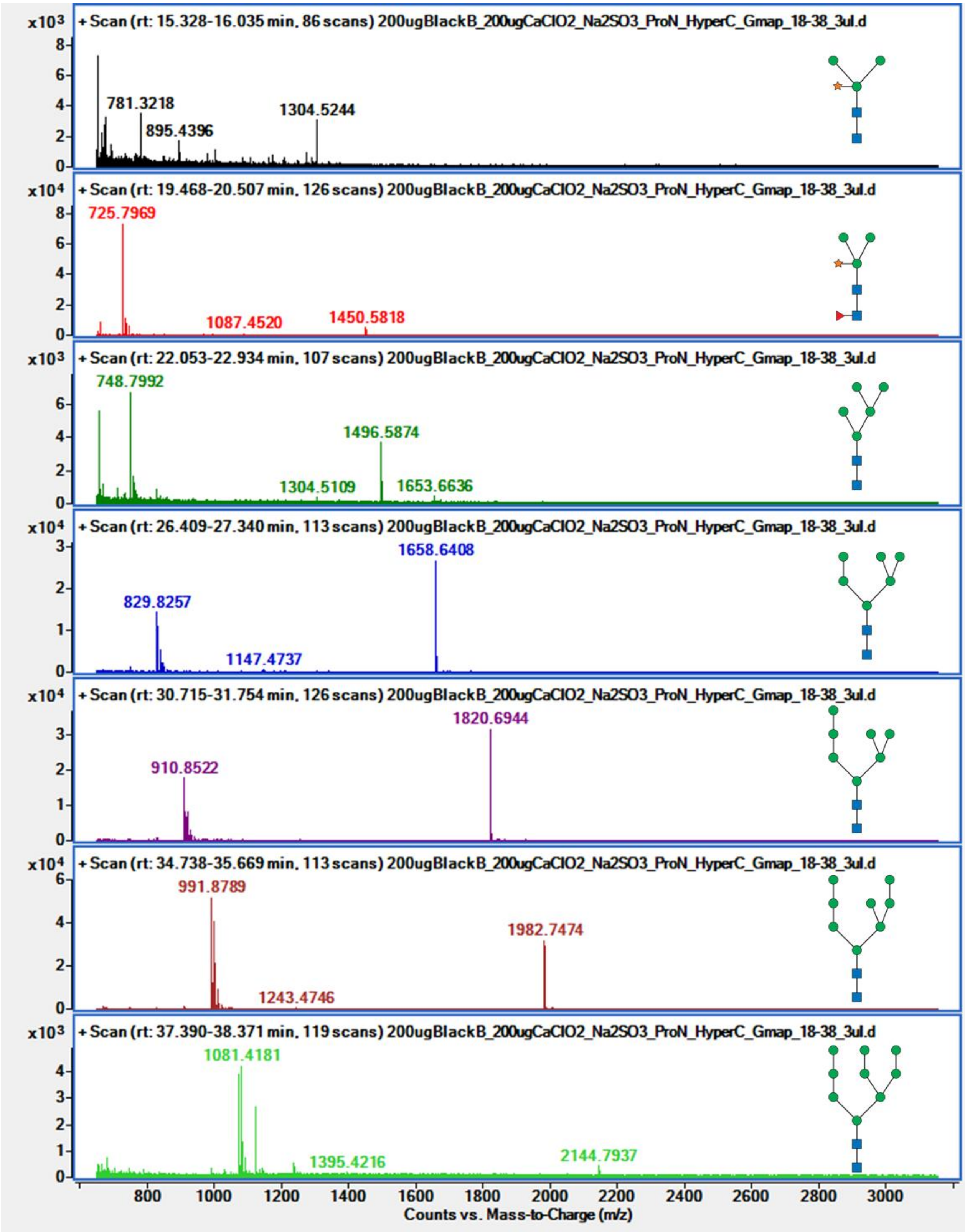

Figure S29. MS profile generated by N-glycan oxidative release and specific labeling protocol of red bean

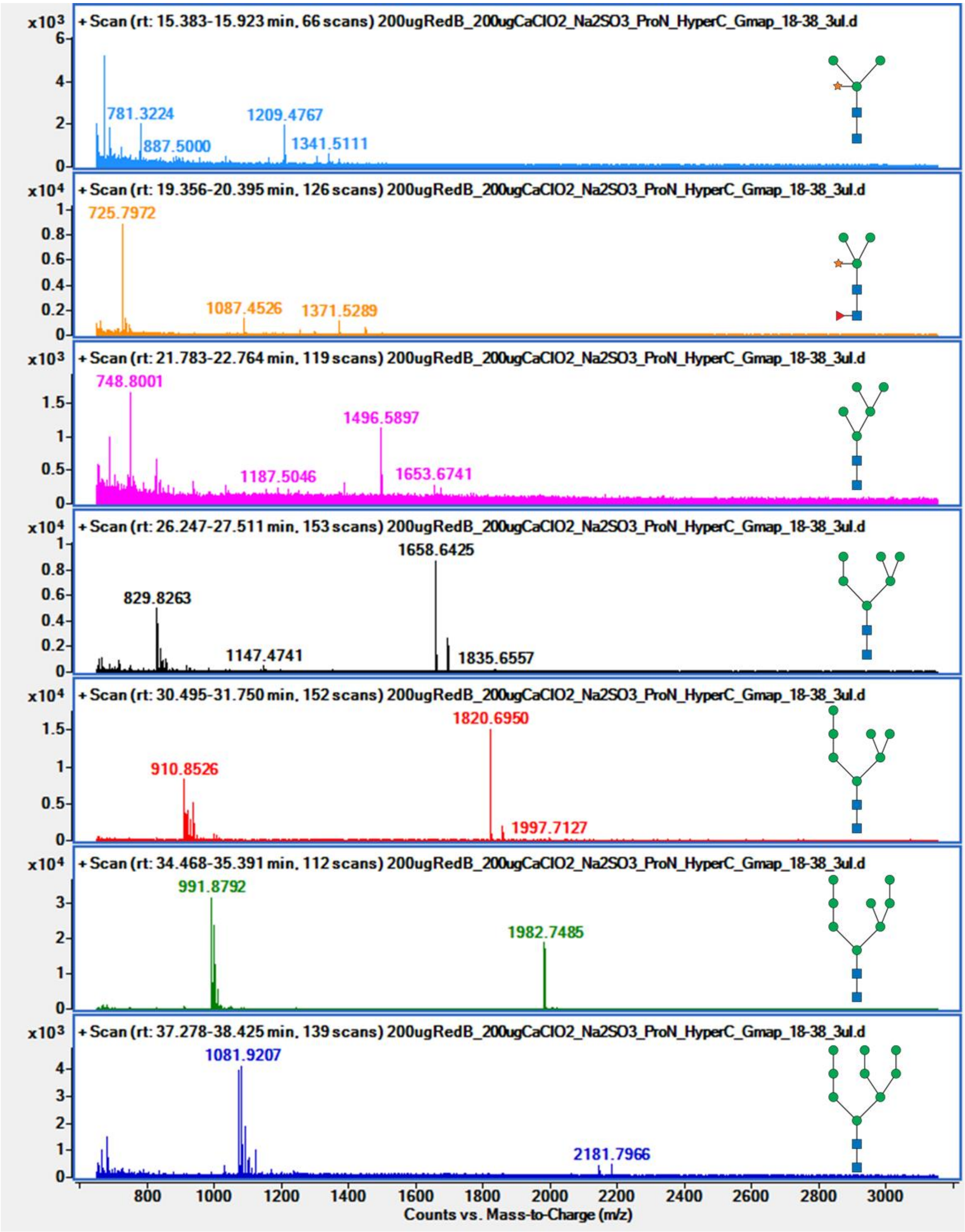

Figure S30. MS profile generated by N-glycan oxidative release and specific labeling protocol of dark red kidney bean

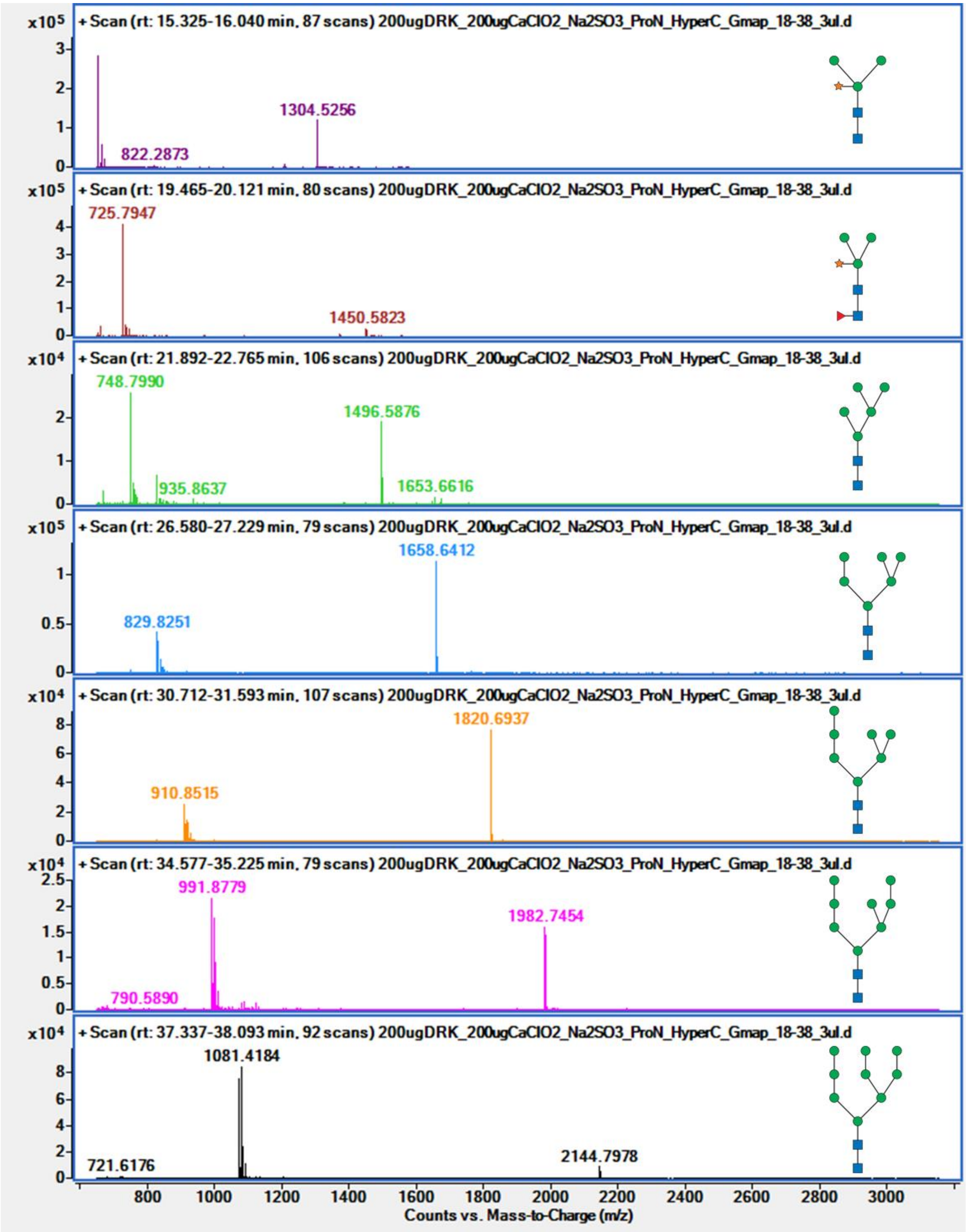

Figure S31. MS profile generated by N-glycan oxidative release and specific labeling protocol of great northern bean

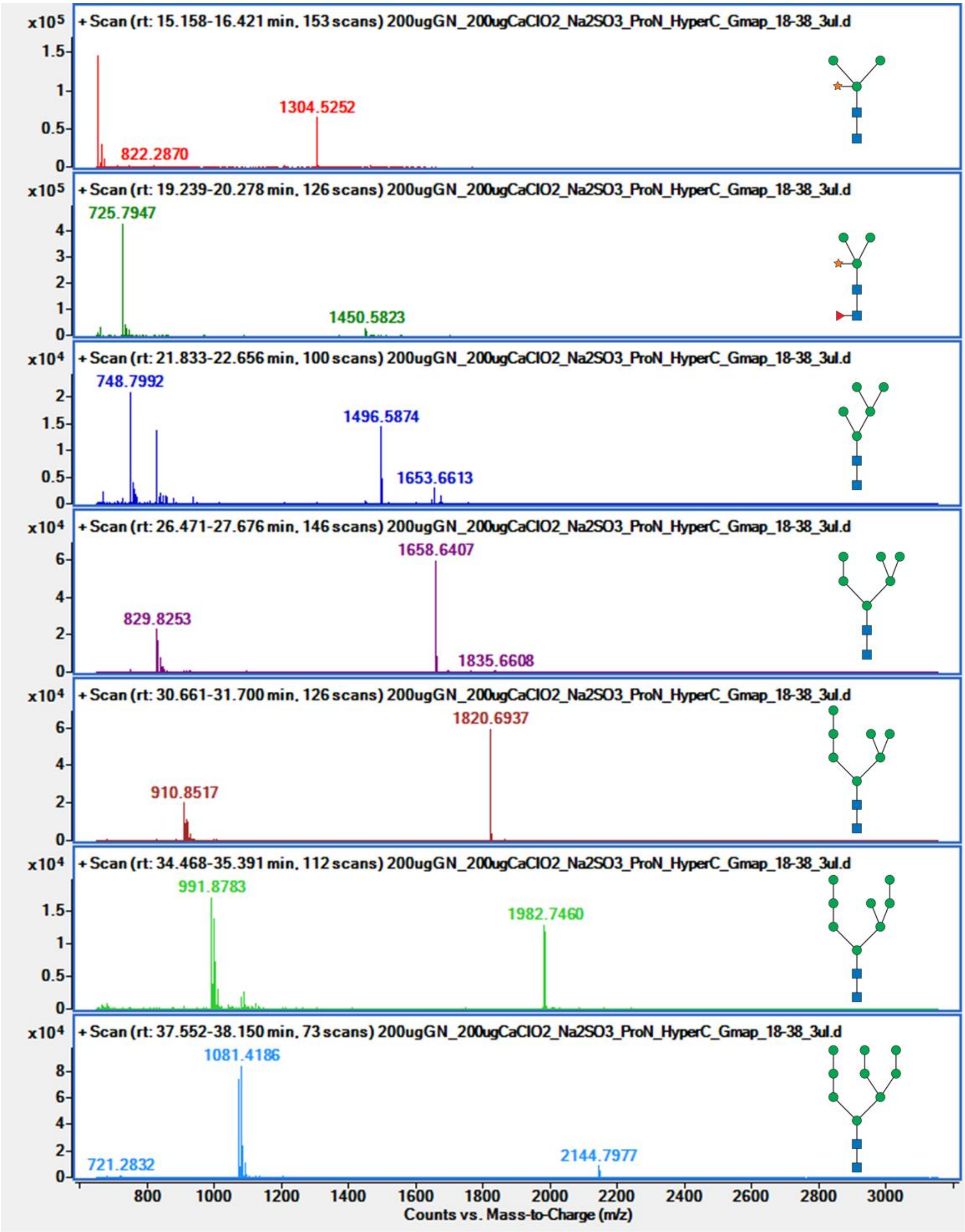

Figure S32. MS profile generated by N-glycan oxidative release and specific labeling protocol of white bean

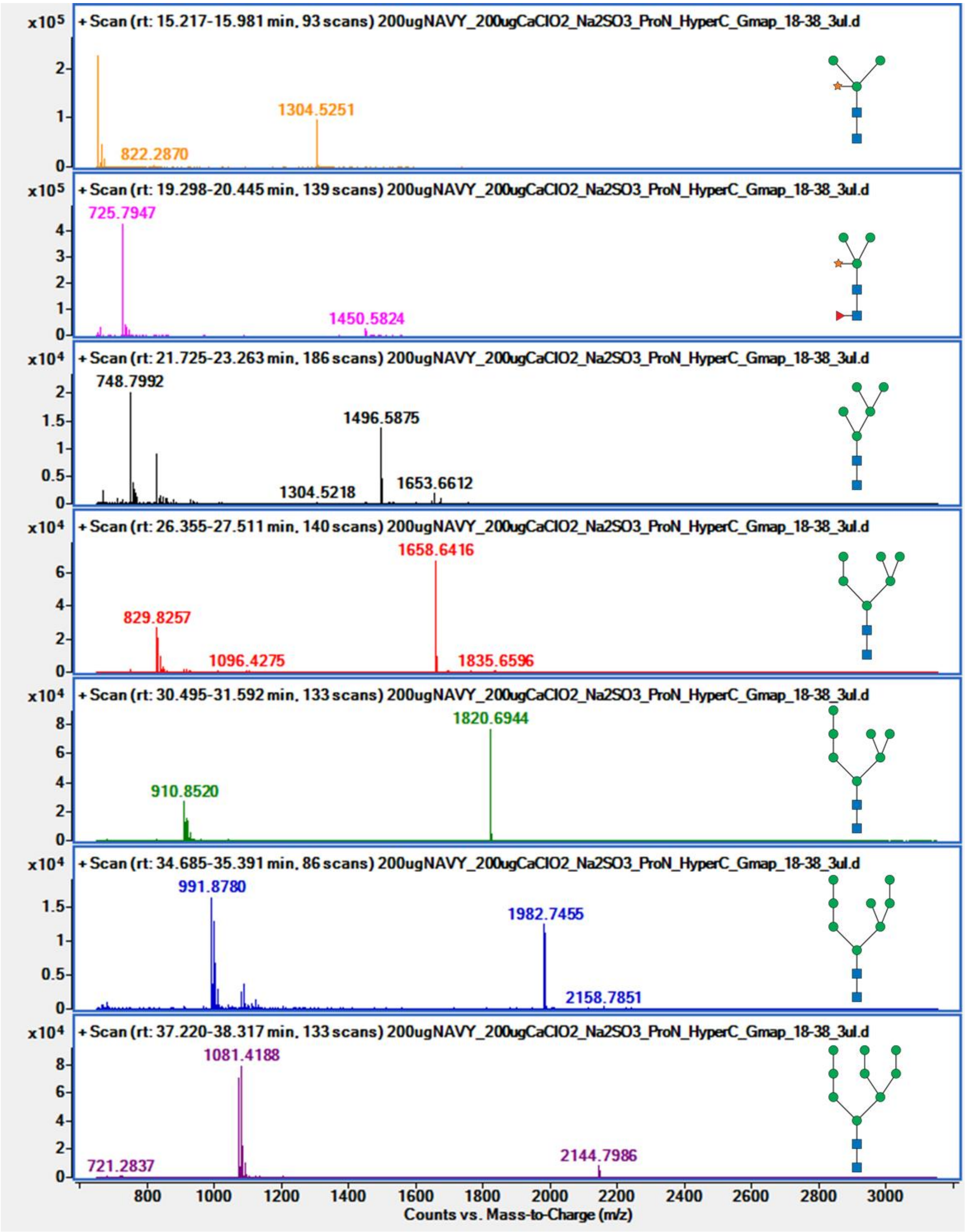

Figure S33. MS profile generated by N-glycan oxidative release and specific labeling protocol of pinto bean

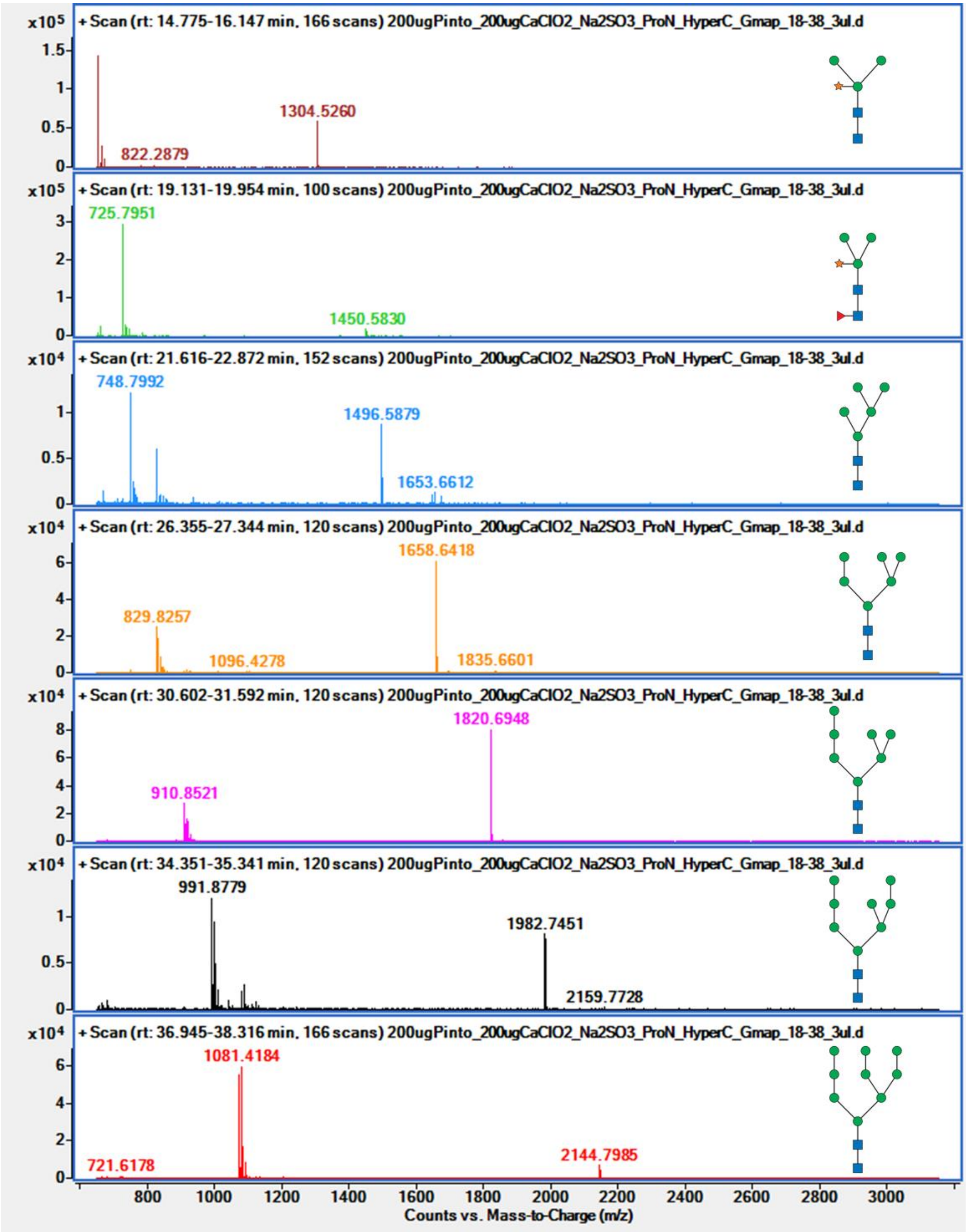

V. NMR Spectra

Figure S34. ^{1}H NMR (600 MHz, D_2O) of Man$_9$GlcNAc$_2$-DCA (**6**)

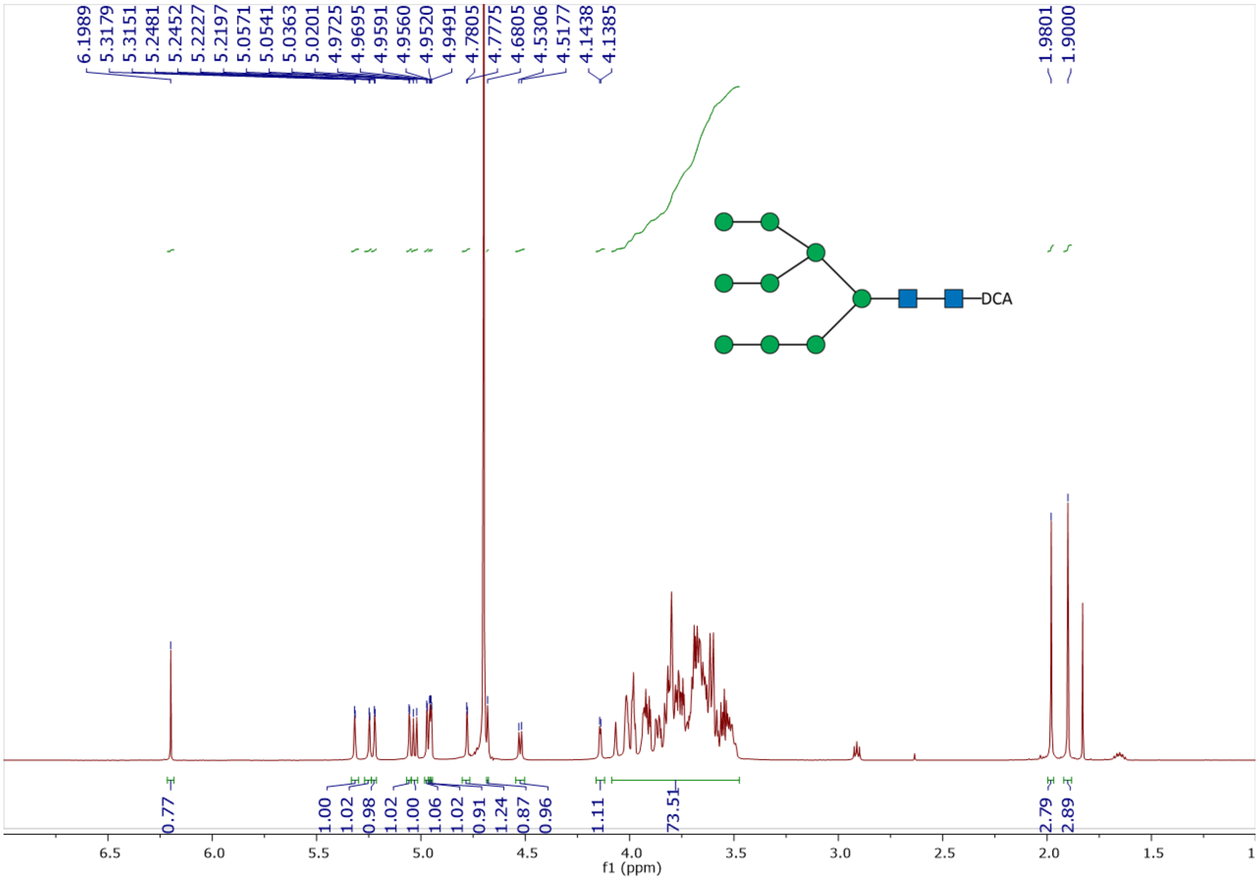

Figure S35. ^{13}C NMR (151 MHz, D_2O) of Man$_9$GlcNAc$_2$-DCA (**6**)

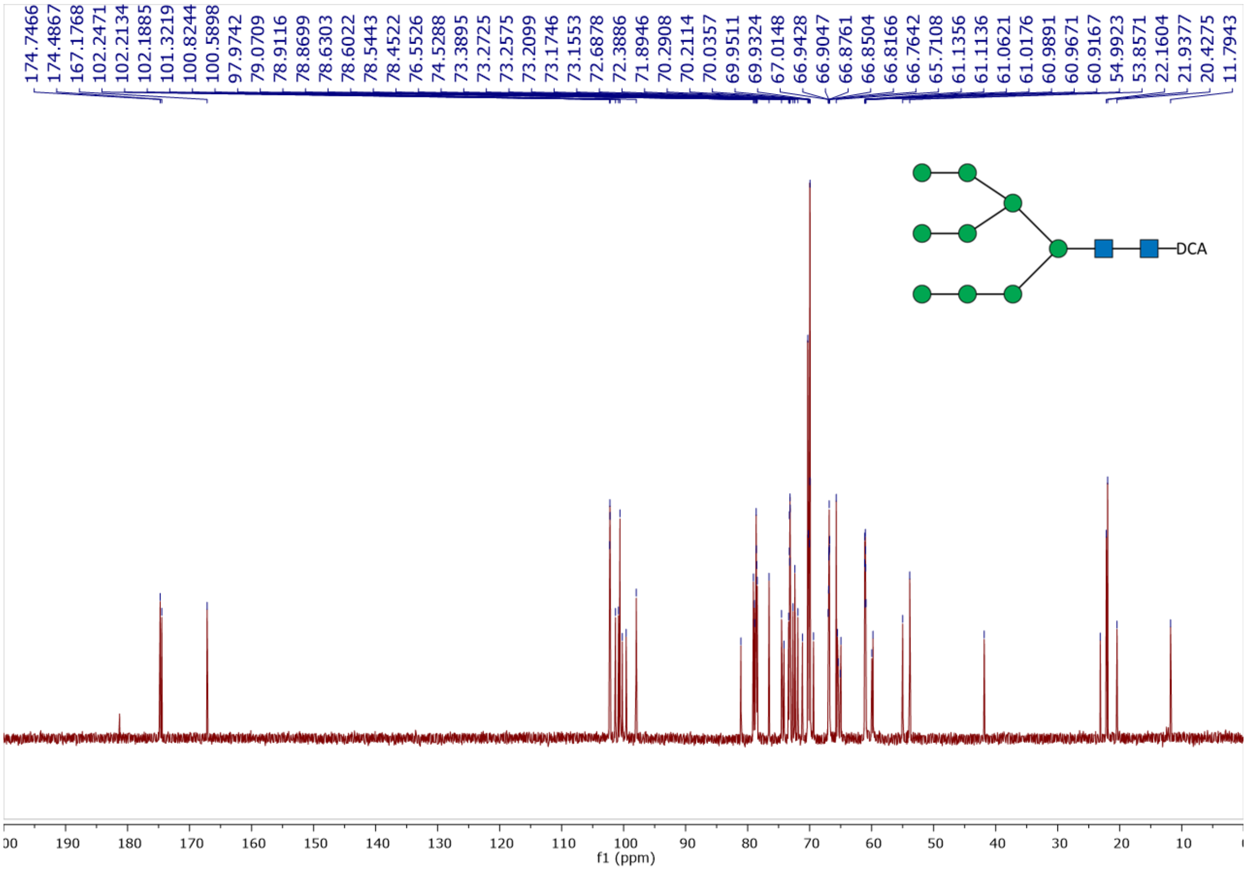

Figure S36. HSQC NMR (600 MHz, D_2O) of Man$_9$GlcNAc$_2$-DCA (**6**)

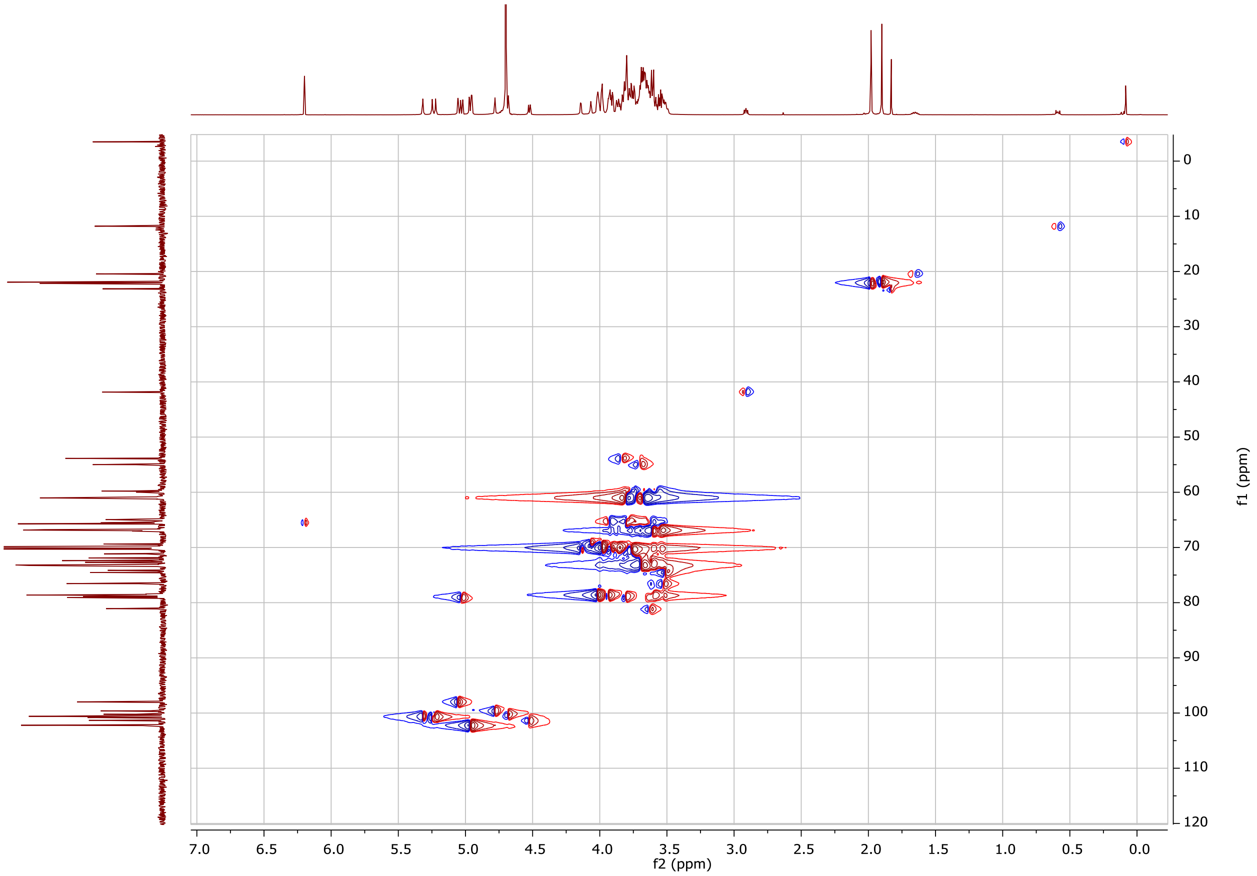

Figure S37. ^{1}H NMR (600 MHz, D_2O) of compound **10**

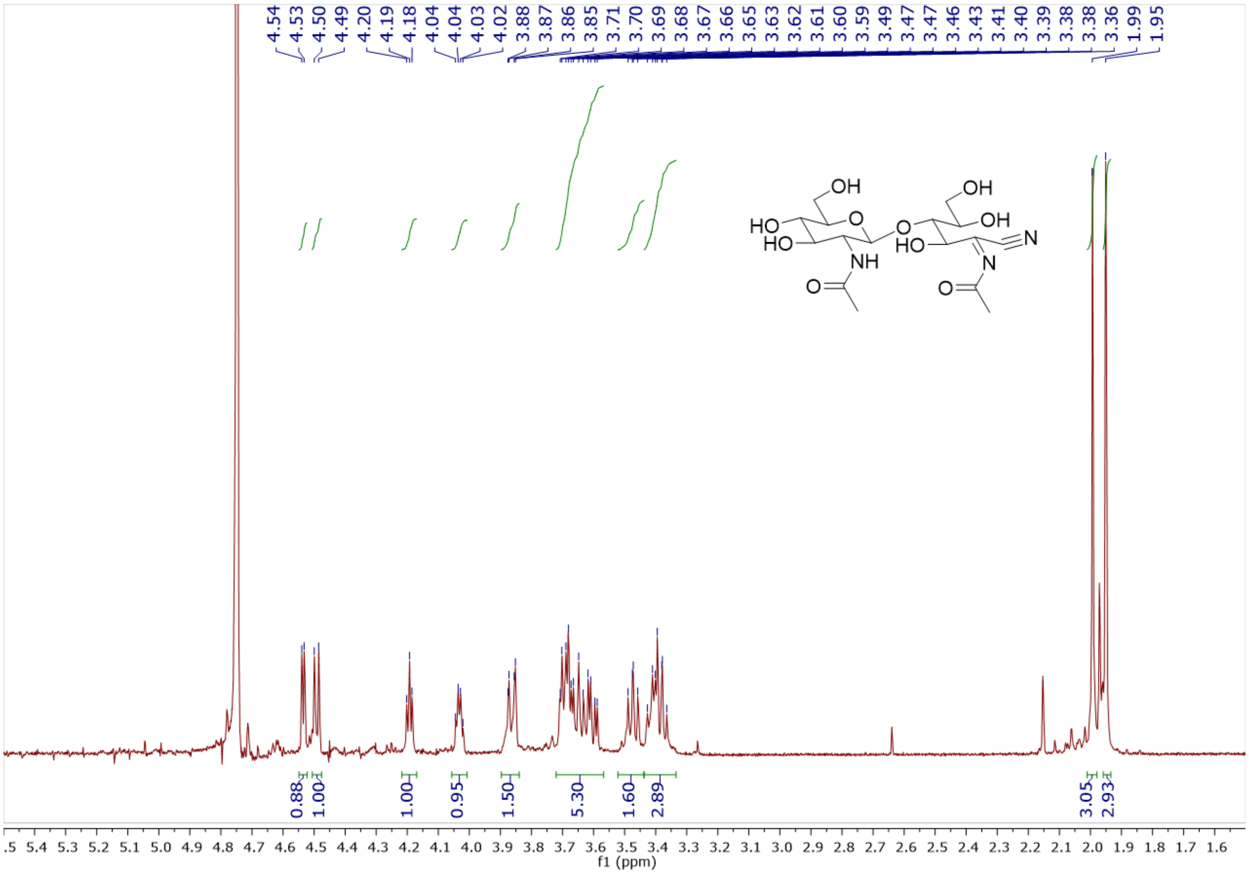

Figure S38. ^{13}C NMR (151 MHz, D_2O) of compound **10**

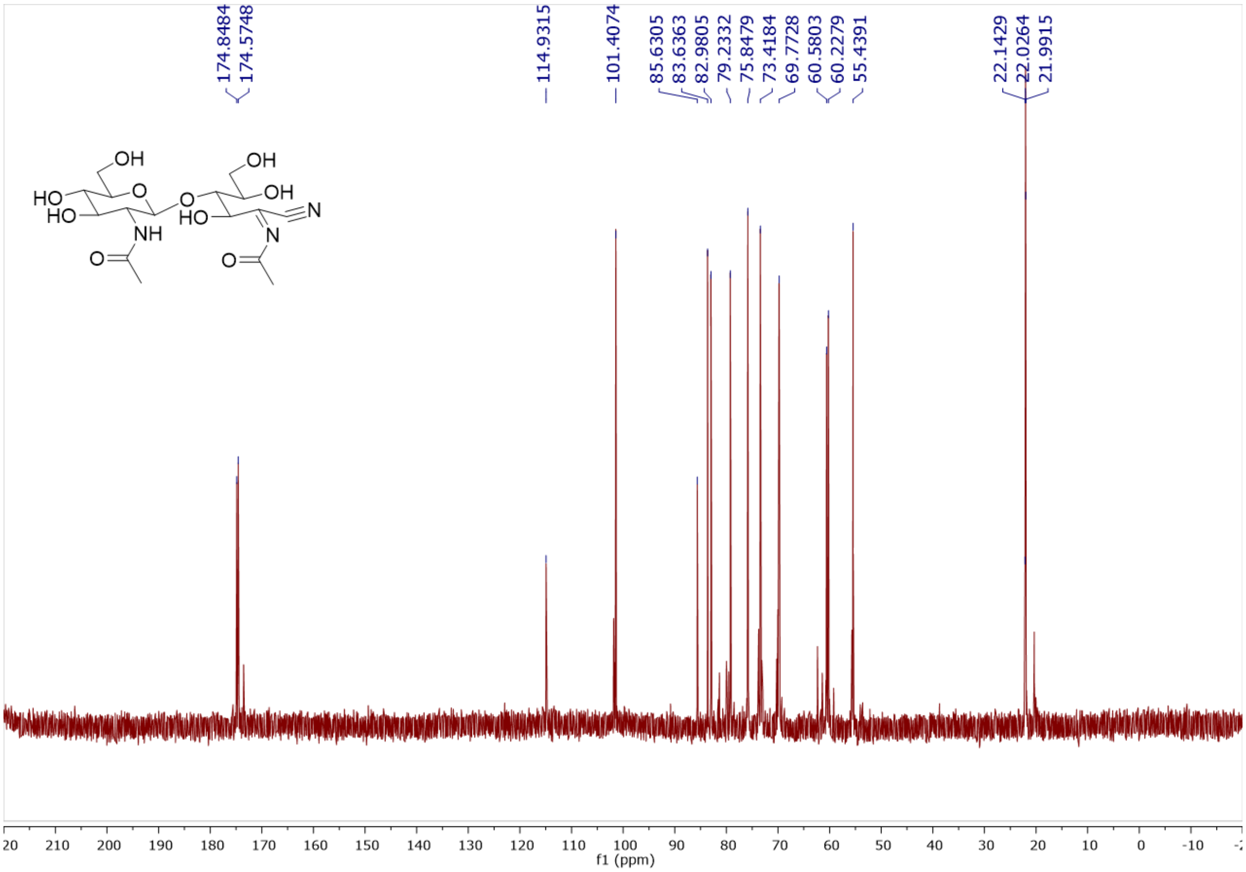

Figure S39. HSQC NMR (600 MHz, D_2O) of compound **10**

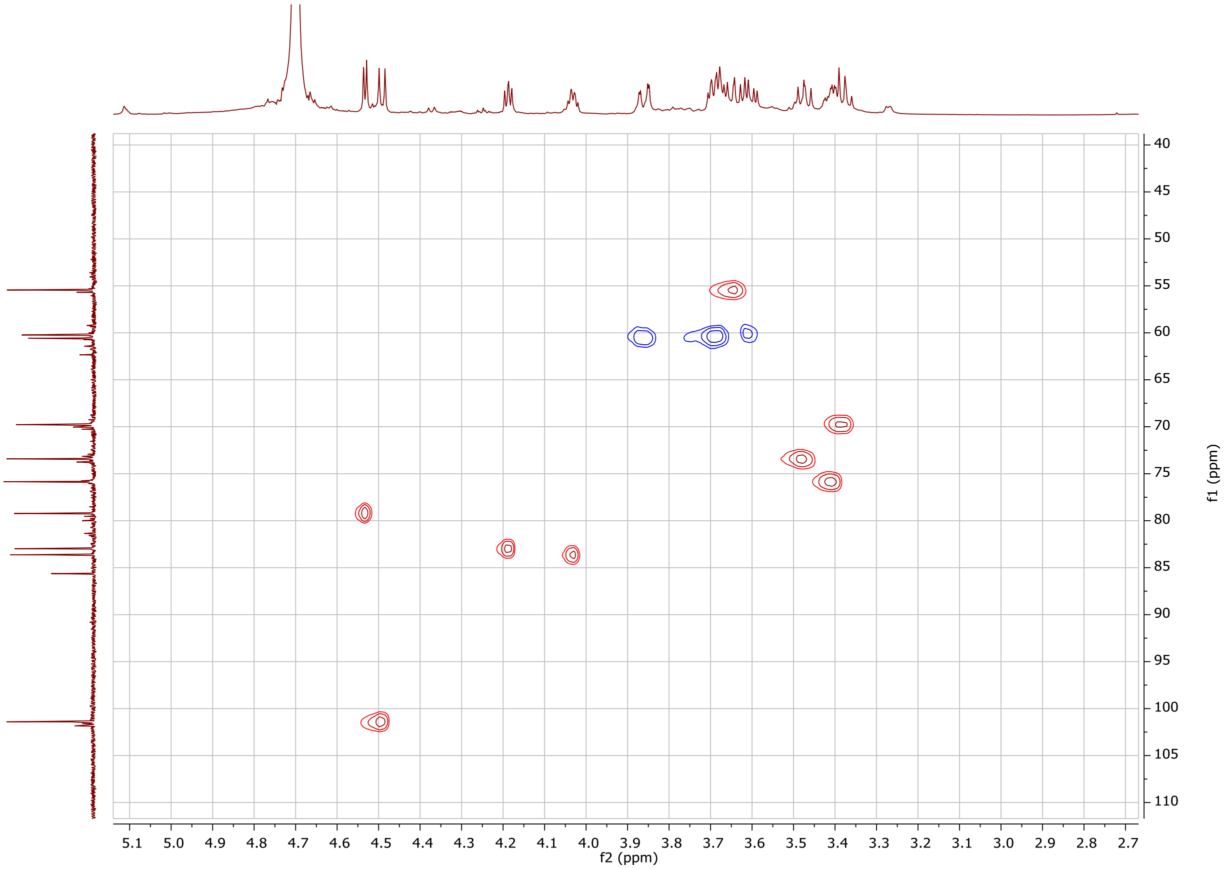

Figure S40. COSY NMR (600 MHz, D_2O) of compound **10**

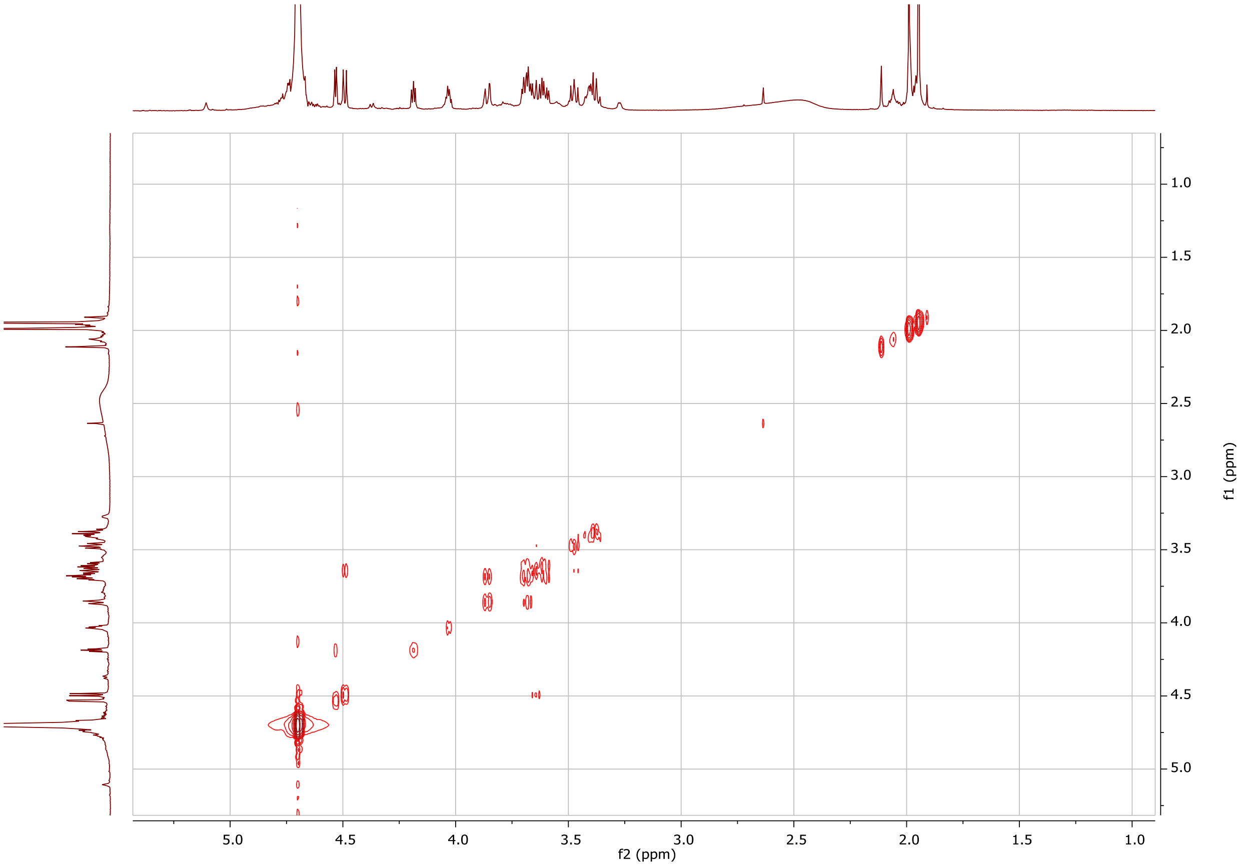

Supplement: Supplementary file 1 — ac4c03246_si_001.pdf [file ac4c03246_si_001.pdf]
